# A counteranion triggered arylation strategy using diaryliodonium fluorides†
†Electronic supplementary information (ESI) available: Experimental data for all new compounds and procedures. See DOI: 10.1039/c4sc02856b



**DOI:** 10.1039/c4sc02856b

**Published:** 2014-11-12

**Authors:** L. Chan, A. McNally, Q. Y. Toh, A. Mendoza, M. J. Gaunt

**Affiliations:** a Department of Chemistry , University of Cambridge , Lensfield Road , Cambridge CB2 1EW , UK . Email: mjg32@cam.ac.uk ; Tel: +44 1223 336318

## Abstract

A mild and transition metal-free counteranion triggered arylation strategy has been developed using diaryliodonium fluorides. The fluoride counteranion within the hypervalent iodonium species displays unusual reactivity that activates a phenolic O–H bond leading to electrophilic *O*-arylation. A wide range of phenols and diaryliodonium salts are compatible with this transformation under remarkably mild conditions. Furthermore, we pre-empt the wider implications of this strategy by demonstrating the compatibility of the arylation tactic with latent carbon nucleophiles.

## Abstract 2

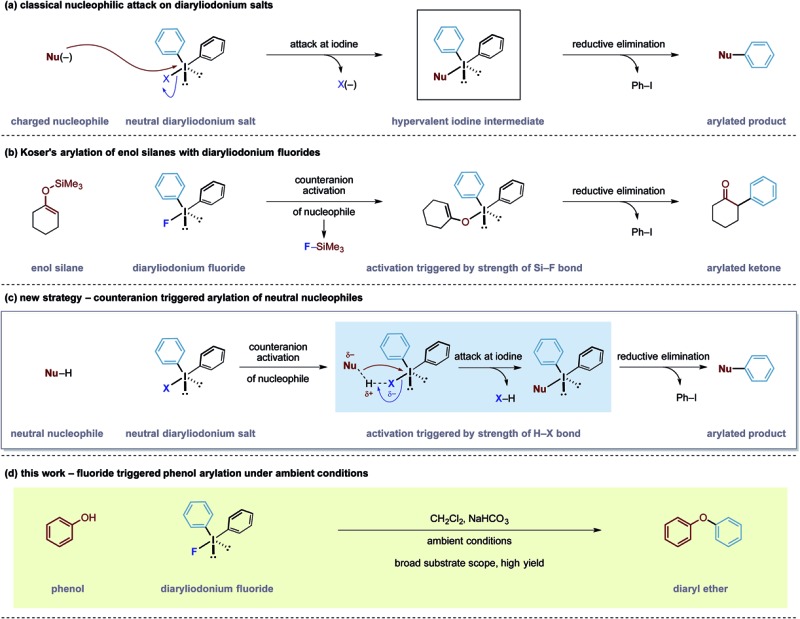
A mild and transition metal-free counteranion triggered arylation strategy has been developed using diaryliodonium fluorides.

## 

Counterion effects can significantly influence the course of a chemical reaction and the ability to control and manipulate these non-covalent interactions represents a non-classical approach to develop new chemical transformations.^1^ Of the many types of counteranion species, fluorides are a unique class of anions that exist in a dichotomy between high stability, due to the electronegativity of the fluorine nucleus, and high reactivity arising from enhanced basicity and nucleophilicity.^2^ Furthermore, reactions can be initiated based on the ability of fluorides to form very strong bonds to molecules containing acidic hydrogen motifs and other atoms such as boron and silicon.^3^

Our group has a long-standing interest in exploiting the reactivity of diaryliodonium salts.^4^ These hypervalent iodine reagents display a T-shaped arrangement of aryl groups about an iodine(iii) atom that can be paired with a variety of counteranions. In both metal-mediated and metal-free reactions of iodine(iii) salts, the counteranion has been shown to have a dramatic effect on the reactivity and selectivity of a given reaction pathway.^5^ Despite the existence of detailed solid state structures, a correlation between the reactivity of diaryliodonium salts and the nature of their counteranion has been difficult to establish.^6^ Despite this knowledge gap, the generally accepted principle is that the counteranion functions as a leaving group for electrophilic aryl species (Scheme 1a). Over the last 6 years, observations made in our laboratories led us to speculate that the counteranions within diaryliodonium salts may sometimes function as more than just a leaving group. As a result, we became interested in bespoke reagents that may build in an additional layer of reactivity into the counteranion unit of these salts, thereby enabling new transformations.

**Scheme 1:**
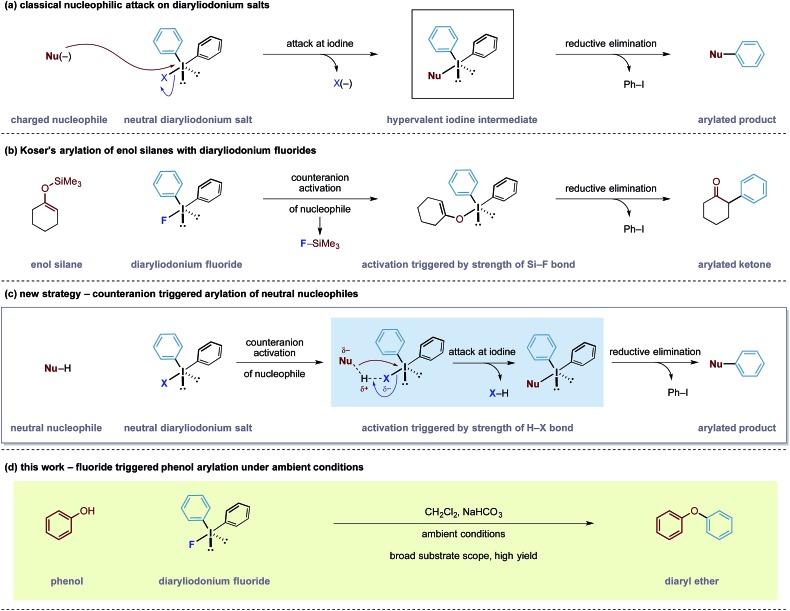
Outline of the counteranion triggered arylation strategy.

In 1991, Koser reported that the reaction between silyl enol ethers and diphenyliodonium fluoride formed α-phenyl ketones.^6^ The reaction does not require any external reagents and is proposed to proceed through an initial salt metathesis that results in Si–F bond formation and an iodonium enolate (Scheme 1b).^7^ Subsequent *O*- to *C*-rearrangement and aryl transfer forms the C–C_aryl_ bond. Despite the apparent simplicity of this process, there have been very few reports employing diaryliodonium fluorides in this manner.^8^ Based on this mechanistic pathway, where the formation of a strong Si–F bond is a key driving force, we were intrigued whether the creation of other strong bonds to fluorine could be used to design useful transformations. Examining bond enthalpy data reveals that forming a H–F bond is highly favourable and should fit this criteria (H–F 135 kcal mol^–1^ *vs.* Si–F 138 kcal mol^–1^).^9^ We envisioned that the fluoride counteranion could function as a base and activate a less reactive nucleophile towards electrophilic arylation (Scheme 1c).

In order to test this hypothesis, we selected the *O*-arylation of phenols as a model reaction. Olofsson and others have shown that this reaction is feasible using diaryliodonium salts providing that the reaction media is sufficiently basic to form reactive phenoxide ions.^10^ These oxy-anions are capable of direct attack at the iodine(iii) centre (Scheme 1a) and a ligand transfer completes the phenol arylation process. Instead, we speculated that the fluoride group within diaryliodonium fluoride may be able to activate the O–H bond through formation of a stable H–F bond, rendering the phenol more nucleophilic and allowing it to undergo simultaneous attack onto the iodine(iii) centre, as shown in Scheme 1c.^11^ An advantage of such an activation strategy is that it would obviate the need for a strongly basic reaction media that may preclude some transformations with sensitive functionality. Herein, we describe the successful realization of this design principle and report an electrophilic arylation strategy with diaryliodonium fluorides for phenols as well as carbon pronucleophiles. The fluoride counteranion activates the nucleophile resulting in a transition metal free arylation process that proceeds under remarkably mild conditions (Scheme 1d).^12^

Our initial experiments to validate the concept of the fluoride counteranion-triggered reactivity are shown in Table 1. A control reaction between phenol and diphenyliodonium triflate (**2a**) in dichloromethane at room temperature resulted in no reaction (entry 1), presumably due to the limited capacity of the phenolic oxygen to act as a nucleophile. However, when diphenyliodonium fluoride was used, we observed formation of diphenyl ether in 18% yield (entry 2). Despite the low yield of this initial reaction, we were encouraged by the fact that the product was formed under these conditions, thereby confirming that our initial hypothesis was valid. We believed that the low yield could be accounted for by the accumulation of HF that might prevent the reaction from proceeding to completion; we observed no further product formation or starting material consumption after reaction for one hour. Addition of a mild base to neutralise the HF generated should therefore allow the reaction to proceed. A screen of mild bases (entries 3–7) revealed that the addition of solid sodium bicarbonate to the reaction mixture led to improvements in both starting material consumption and product formation giving a yield of 95% after reaction for 30 minutes in dichloromethane solution (entry 3).

**Table 1:** Reaction optimisation^*a*^

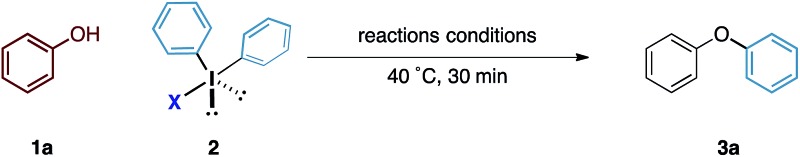				
Entry	**X**	Base	Solvent	Yield^*b*^ (%)
1	OTf (**2a**)	—	CH_2_Cl_2_	0
2	F (**2b**)	—	CH_2_Cl_2_	18
3	F	NaHCO_3_	CH_2_Cl_2_	95
4	F	KHCO_3_	CH_2_Cl_2_	32
5	F	Na_2_CO_3_	CH_2_Cl_2_	84
6	F	K_2_CO_3_	CH_2_Cl_2_	47
7	F	Cs_2_CO_3_	CH_2_Cl_2_	92
8	F	NaHCO_3_	PhMe	42
9	F	NaHCO_3_	MeCN	90
10	F	NaHCO_3_	THF	50
11	F	NaHCO_3_	MeOH	15
12	OTf (**2a**)	NaHCO_3_	CH_2_Cl_2_	0

^*a*^ **1** equiv. of **1a**, 1.2 equiv. **2**, 2.4 equiv. base, 0.05 M concentration.

^*b*^GC yield with triphenylmethane as the internal standard.

A survey of the reaction parameters showed that although reaction in dichloromethane proved the most efficient, a range of polar and non-polar solvents also worked, in some cases giving comparable yields. Significantly, no reaction was observed where diphenyliodonium triflate was employed even in the presence of sodium bicarbonate (entry 12), further confirming the unique reactivity of the diaryliodonium fluoride system.

With an optimal set of reaction conditions in hand, we next examined the scope of the new process using a range of substituted phenols (Table 2). At 40 °C, the reaction time varied from 20 minutes to 6 hours across the different substrates and good to excellent isolated yields were obtained (**3a–r**). Phenols with electron-withdrawing substituents worked equally well as those with electron-donating substituents. Interestingly, **3e** was formed selectively without observation of arylation at the benzylic alcohol, giving the desired product in good yield. *Ortho*-substituted phenols proceeded smoothly under the standard reaction conditions (**3f–i**, **k**) as exemplified by the successful arylation of the hindered 1,1′-bi-2-naphthol (**3m**) in excellent yield. A substrate displaying an electron rich heterocycle also gave the desired diaryl ether **3n** in almost quantitative yield demonstrating the preferential reactivity of the phenol over other nucleophilic moieties.^13^ Phenols containing Lewis basic heterocycles, such as hydroxypyridines and quinolines, were also compatible with this transformation, giving moderate to good yields (**3o–r**).

**Table 2:** Scope of phenyl component^*a*^

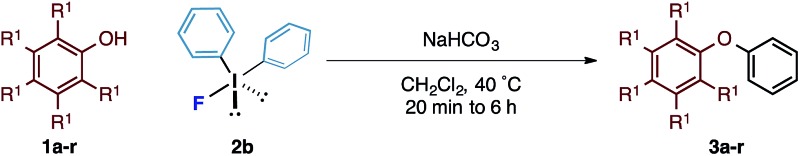
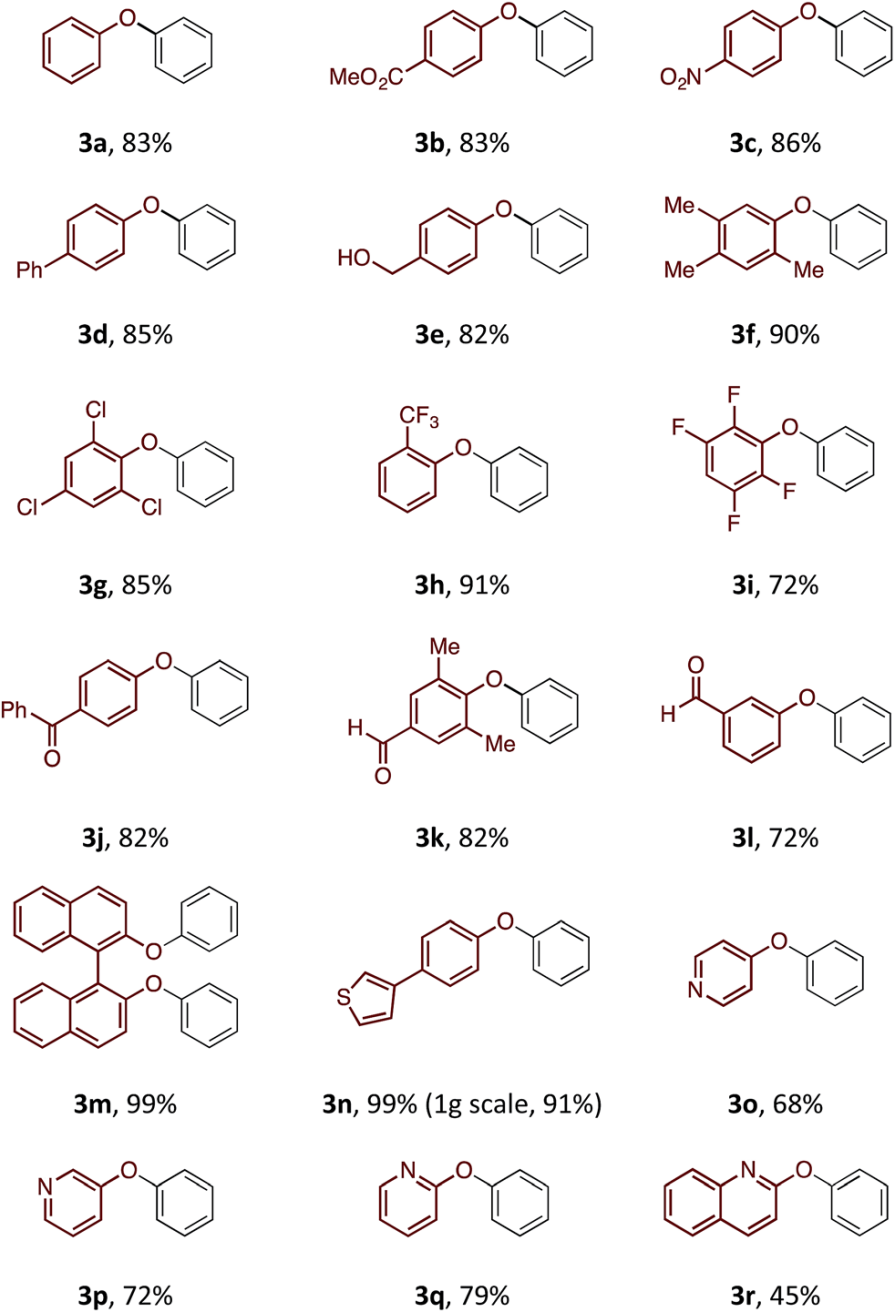

^*a*^ **1** equiv. of **1**, 1.2 equiv. **2b**, 2.4 equiv. NaHCO_3_, 0.05 M concentration. Yields are quoted after isolation and purification *via* silica gel chromatography.

Although the synthesis of diaryliodonium fluorides is relatively trivial using standard literature procedures,^7,8^ we questioned whether we could directly generate the fluoride counterparts *in situ* from more readily available diaryliodonium triflates (and tetrafluoroborates) and an external fluoride additive in the reaction. This would enable the transfer of a wide range of aryl groups from the more readily available, and commercial diaryliodonium triflates. Therefore, we tested whether our *O*-arylation process could be initiated by the *in situ* combination of diphenyliodonium triflate and one equivalent of tetrabutyl ammonium fluoride (TBAF). We were pleased to find that a range of substituted diaryliodonium salts worked well in the reaction to give a range of diaryl ethers in good to excellent yield (Table 3). Neither electron-rich nor electron-deficient salts posed a problem. Steric hinderance imparted from the *ortho*-substituent in the corresponding diaryliodonium salts did not pose any difficulties. In line with the previously observed selectivity of electronically unsymmetric diaryliodonium salts (4-NO_2_–C_6_H_4_ *vs.* C_6_H_5_) under transition metal free conditions, exclusive transfer of the electron-deficient aryl group was observed to form **3c**.^7,8,14^

**Table 3:** Scope of aryl transfer^*a*^

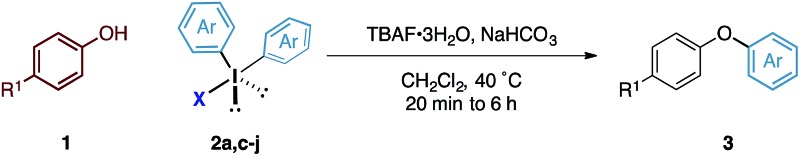
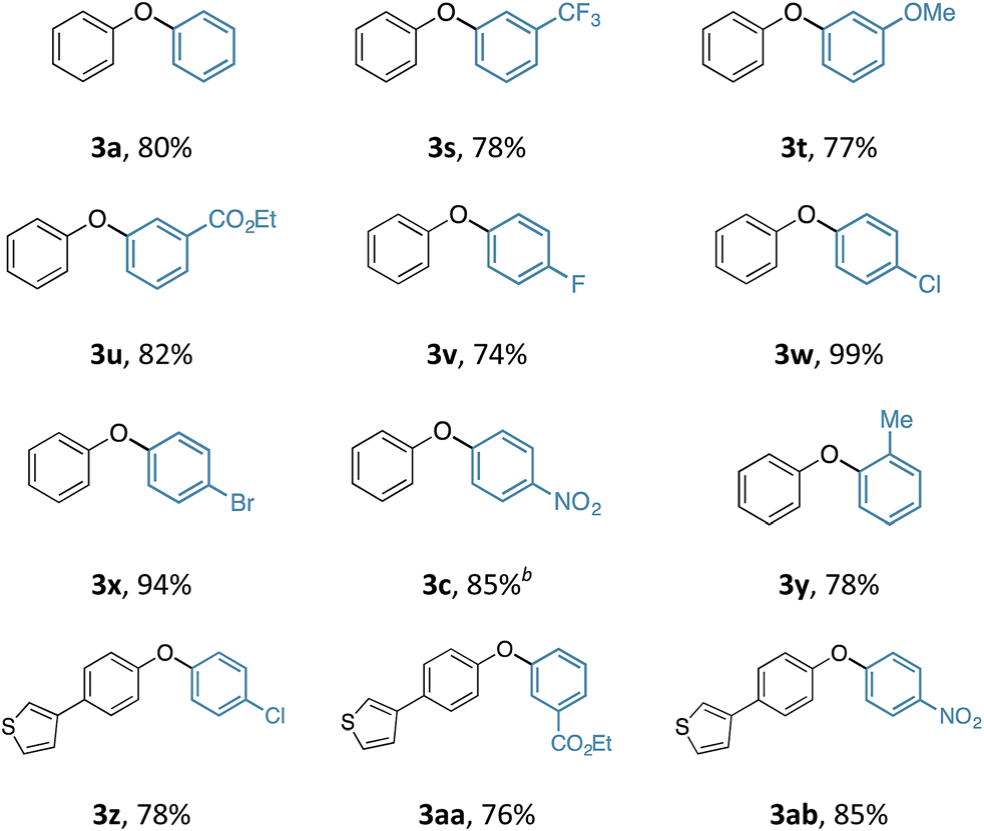

^*a*^ **1** equiv. of **1**, 1.2 equiv. **2**, 1.2 equiv. TBAF·3H_2_O, 2.4 equiv. NaHCO_3_, 0.05 M concentration.

^*b*^Reaction with [(4-NO_2_–C_6_H_4_)–I–(C_6_H_5_)]OTf. All yields are quoted after isolation and purification *via* silica gel chromatography.

Under our previously reported copper-catalyzed arylation tactic, we had shown that estrone underwent *ortho*-arylation of the resident phenolic functionality (Scheme 2a).^15^ When we tested estrone under the reactions conditions using diphenyliodonium fluoride we observed exclusive *O*-arylation of the steroid **10** in excellent yield (Scheme 2b), thereby demonstrating the orthogonal reactivity of the diaryliodonium fluorides compared to other more conventional iodonium species.

**Scheme 2:**
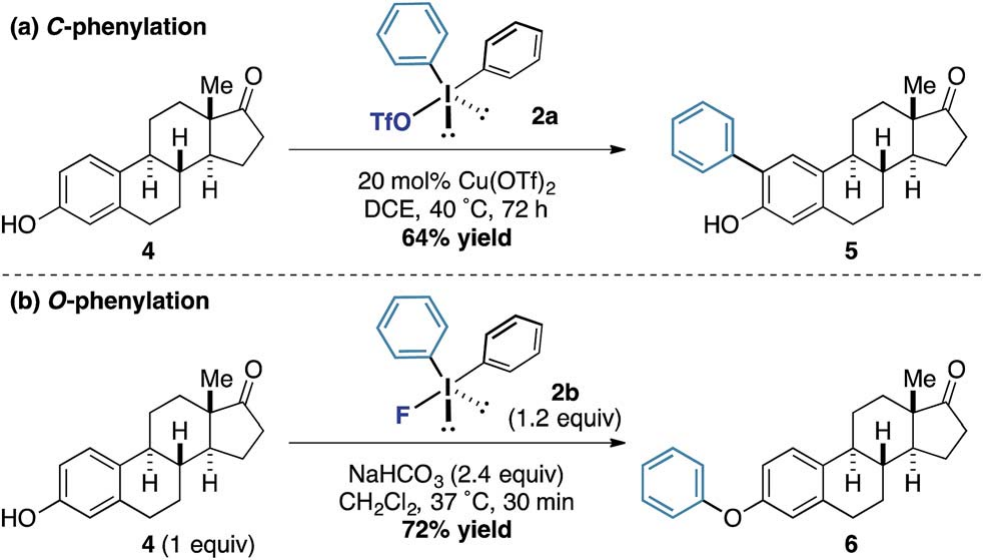
Chemoselective phenylations of estrone.

l-Tyrosine derivatives and small peptides containing this amino acid were also responsive to the new arylation process. Protected tyrosine derivative **7** was submitted to the standard reaction conditions and an excellent yield of the arylated product **8** was obtained after only 30 minutes without loss of enantiomeric purity (Scheme 3a). A tripeptide **9** also worked well and phenol motif undergoes arylation without interference from other functionalities to form the product **10** in 84% yield (the reaction proceeded in under 2 hours and even at reaction concentrations as low as 1 mM, Scheme 3b). Taken together, these results offer the potential for bioorothogonal arylative functionalization strategies.^16^

**Scheme 3:**
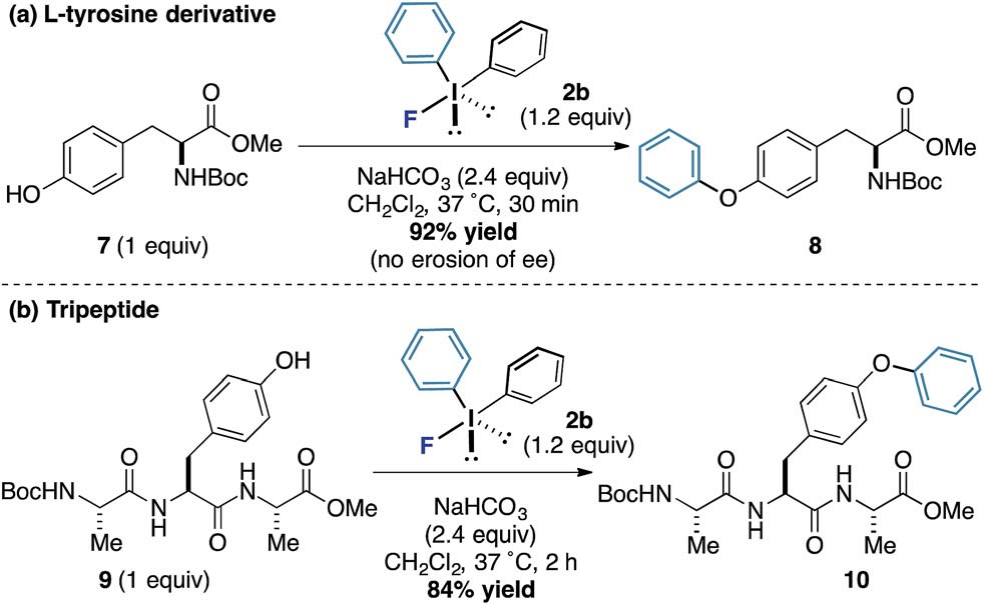
Phenylation of tyrosine derivative and a tripeptide.

Finally, we assessed the broader potential of the diaryliodonium fluorides in other systems. Considering the p*K*_a_ of the average phenol in water is around 10, we investigated acidic carbon centred pronucleophiles with p*K*_a_'s in the same range as phenols (Scheme 4a). We found that the latently nucleophilic α-cyano, α-phenyl esters (**11**) underwent *C*-phenylation in excellent yield under identical reaction conditions to the phenol system to form the all carbon quaternary centred ester **12**.^17^ Additionally, the diaryliodonium triflate – TBAF combination also enabled the transfer of substituted aryl derivatives (to **13**) in good yields. To further explore the reactivity of the counteranion effect, we were also able to demonstrate related reactions using the boron containing pronucleophiles by exploiting the strength of the B–F bonds (Scheme 4b). Under similar conditions, a metal-free cross coupling to form biphenyl **15** could be affected through the combination with triphenyl borane **14** and diphenyliodonium fluoride using no additional reagents other than reaction solvent. Similarly, the combination of diaryliodonium triflate and TBAF with the triphenyl borane provided the unsymmetrical biaryl **16** in good yield. While we acknowledge the latter transformations to form biaryls are inefficient in terms of ‘aryl economy’, the underpinning mechanistic pathway provides an interesting conceptual strategy to the formation of this fundamental carbon framework.

**Scheme 4:**
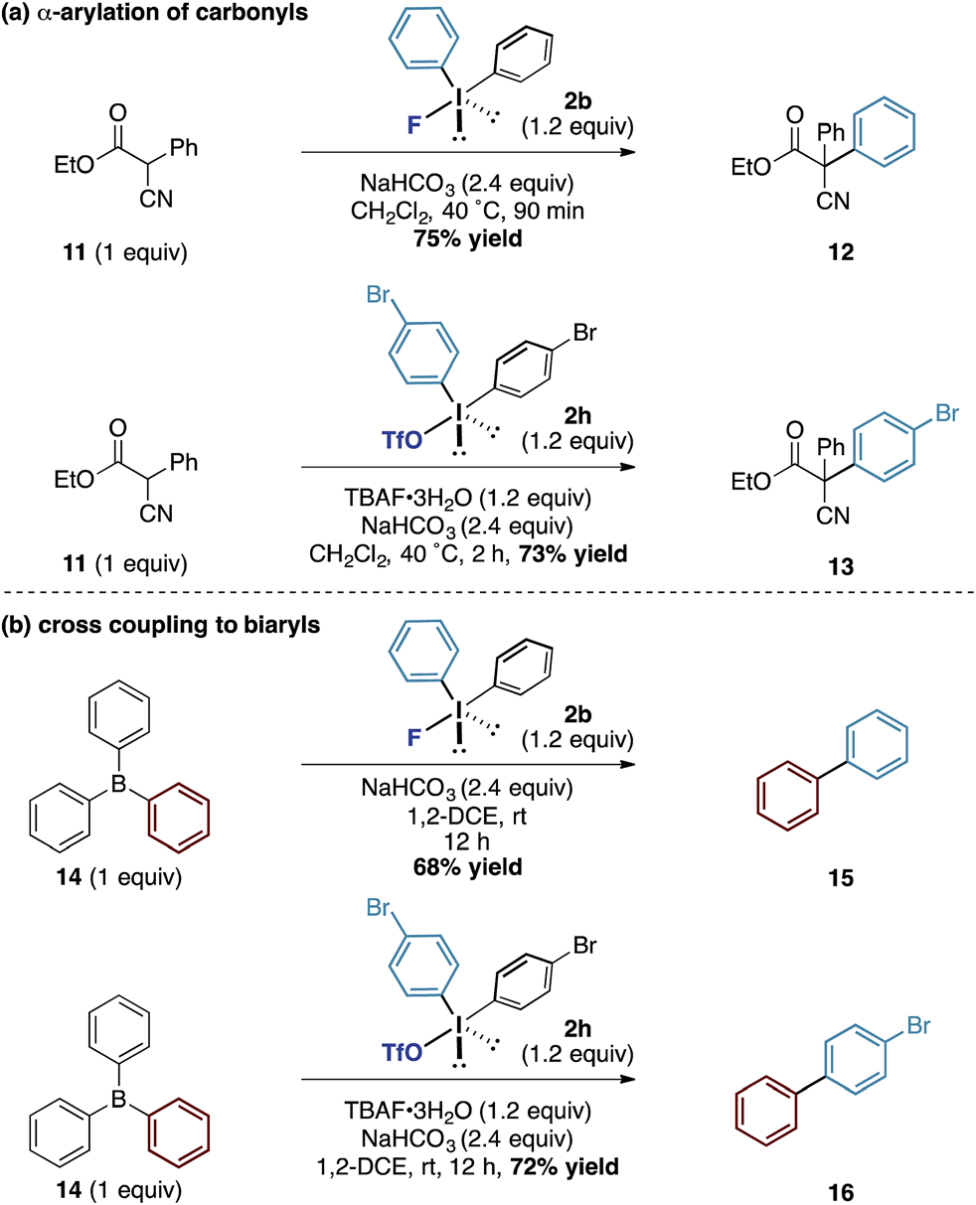
α-Arylation of carbonyl compounds.

## Conclusions

In summary, we have developed a counteranion controlled strategy for transition metal free electrophilic arylation with diaryliodonium fluorides. The activation process is demonstrated *via* the arylation of phenols to form diaryl ethers under ambient conditions. This transformation is tolerant to a wide range of functional groups on both the phenol building blocks and diaryliodonium reagents. We believe that the reaction is initiated by hydrogen bonding between the fluoride counterion and the phenolic O–H, which enhances the nucleophilicity of the phenol and triggers the attack at the iodine(iii) centre displacing the fluoride as a leaving group. Subsequent ligand coupling from the iodine atom generates the aryl ether. During our exploration of the reactivity of these diaryliodonium fluorides, we have identified that the reaction has some potential as a bioorthogonal arylation process and that it can be expanded to accommodate carbon pronucleophiles leading to complex aryl products. Current studies are focused on these lead results and a clearer mechanistic understanding the activation process; these results will be reported in due course.
