# Supplementary figures and images for: A counteranion triggered arylation strategy using diaryliodonium fluorides
Source: Chem Sci. 2014 Nov 12;6(2):1277–81. doi: 10.1039/c4sc02856b (PMC5811167; doi:10.1039/c4sc02856b)

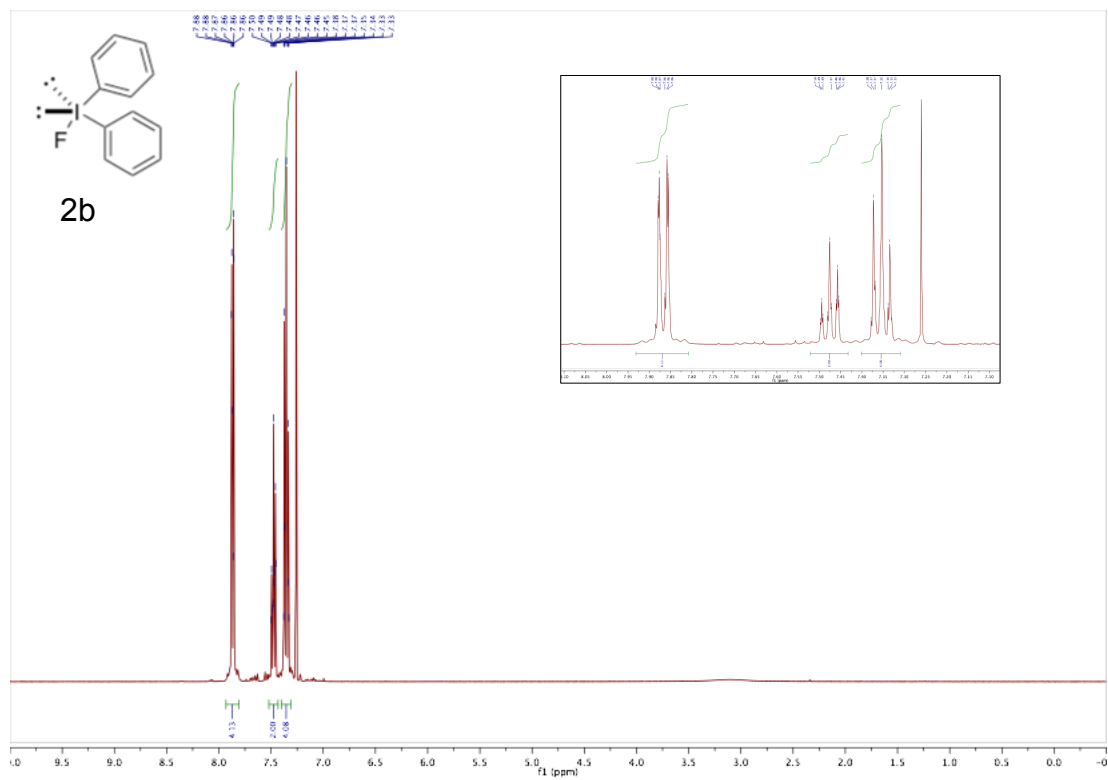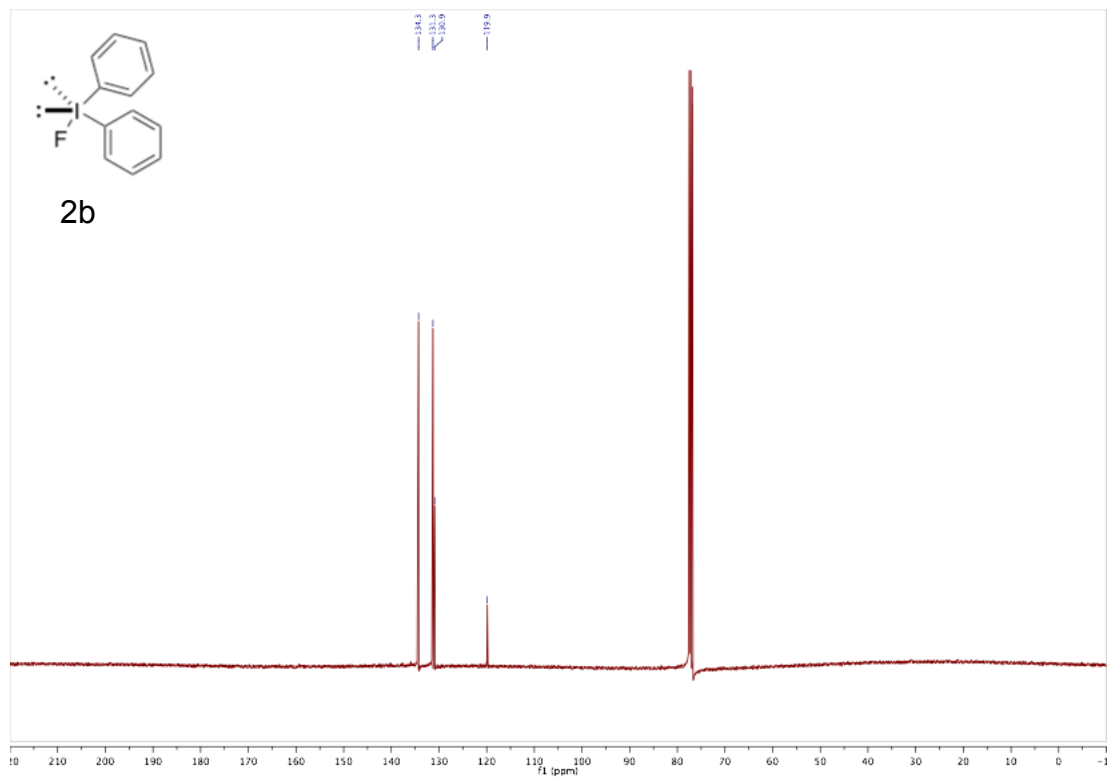

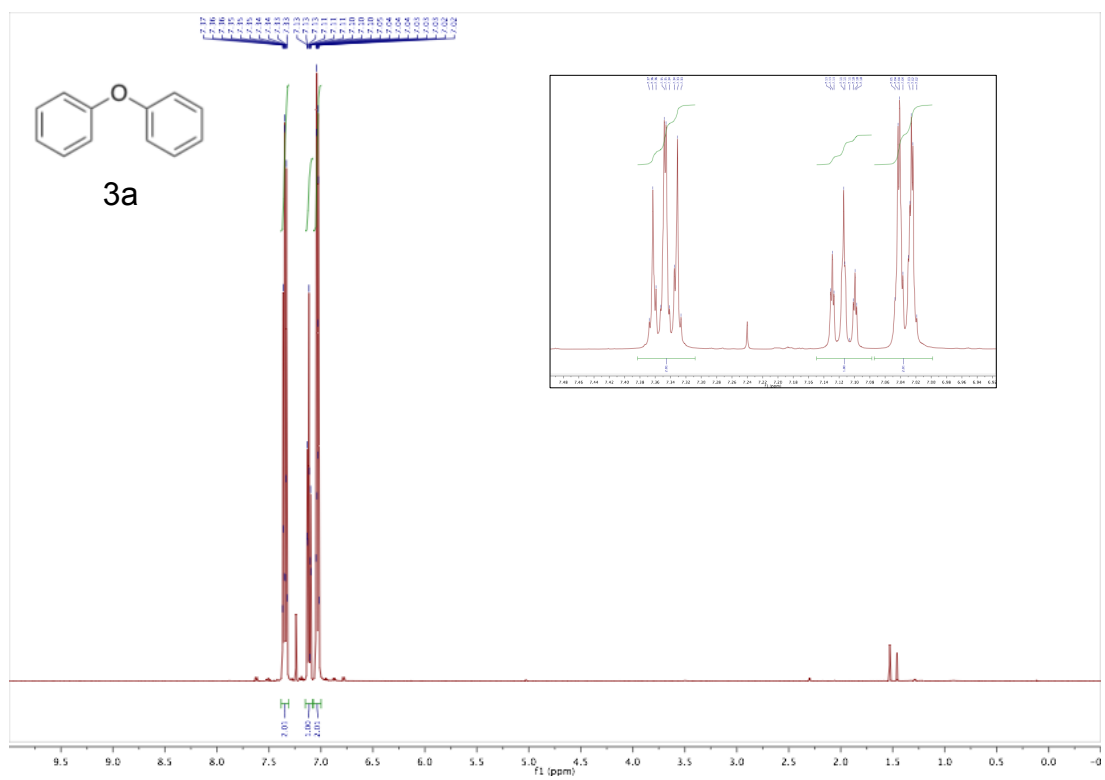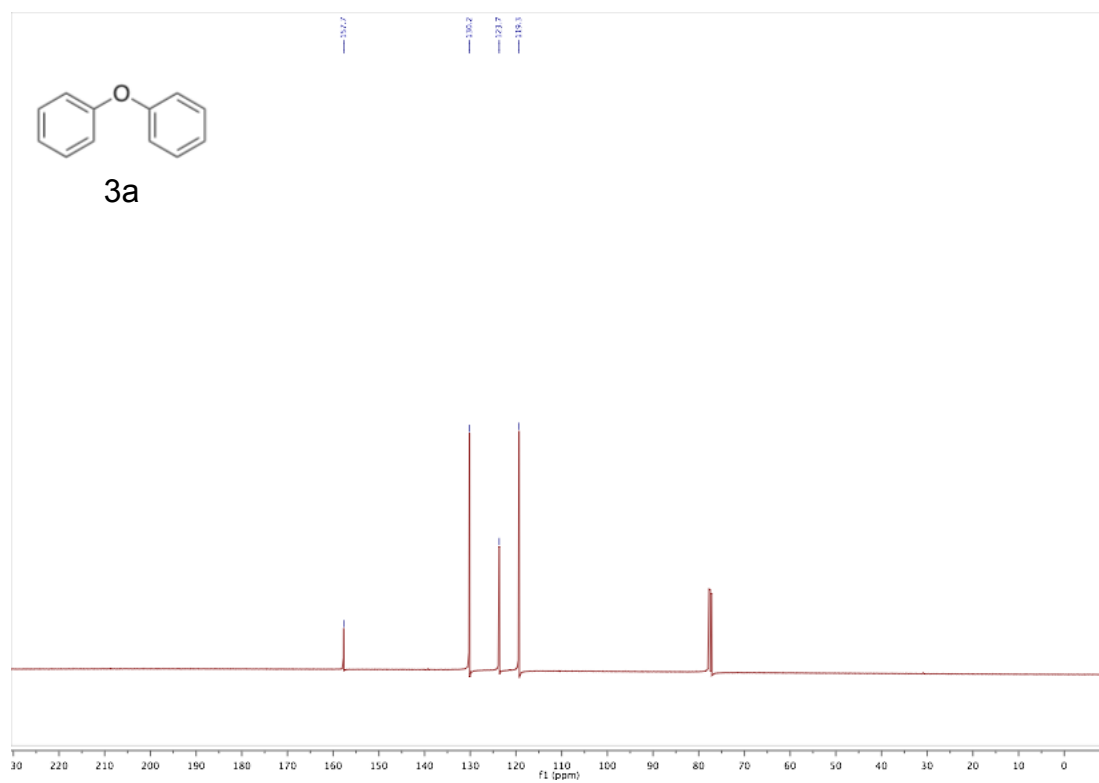

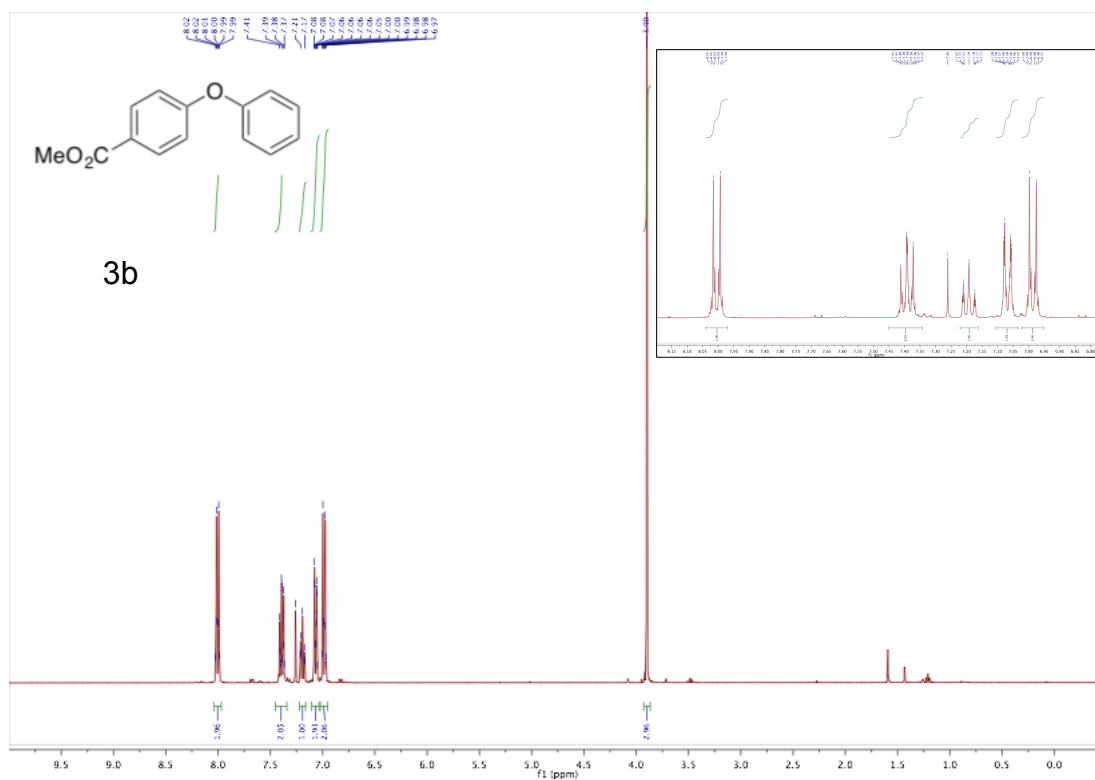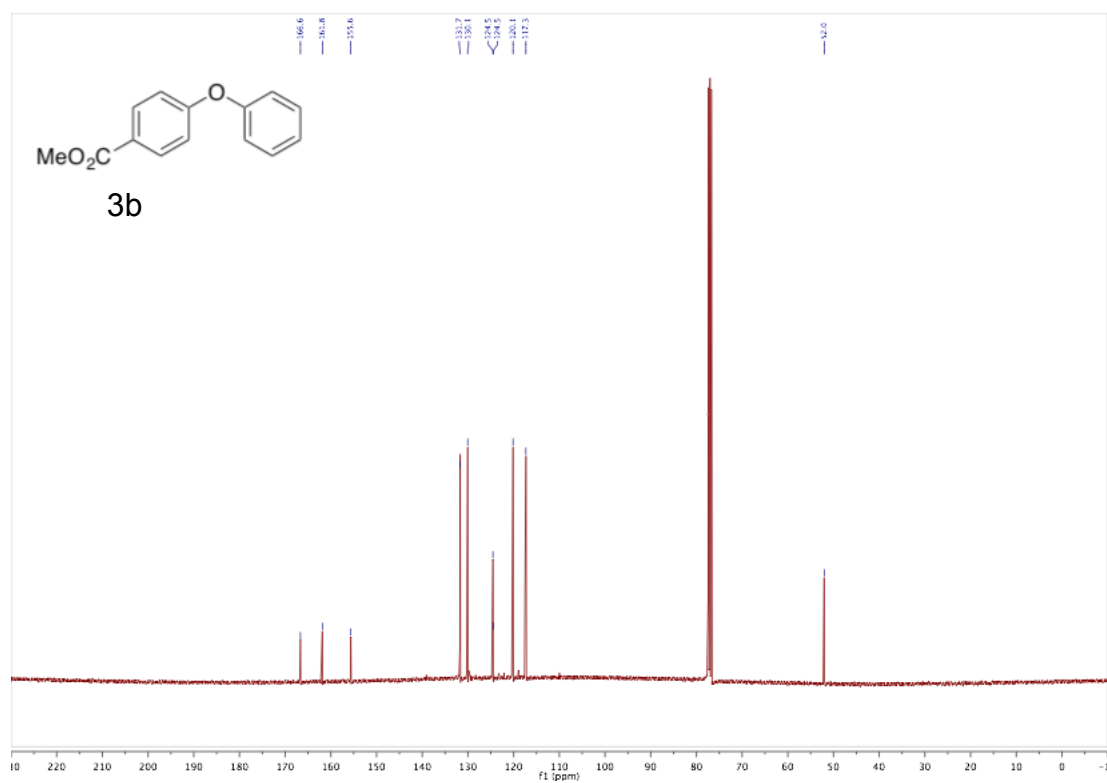

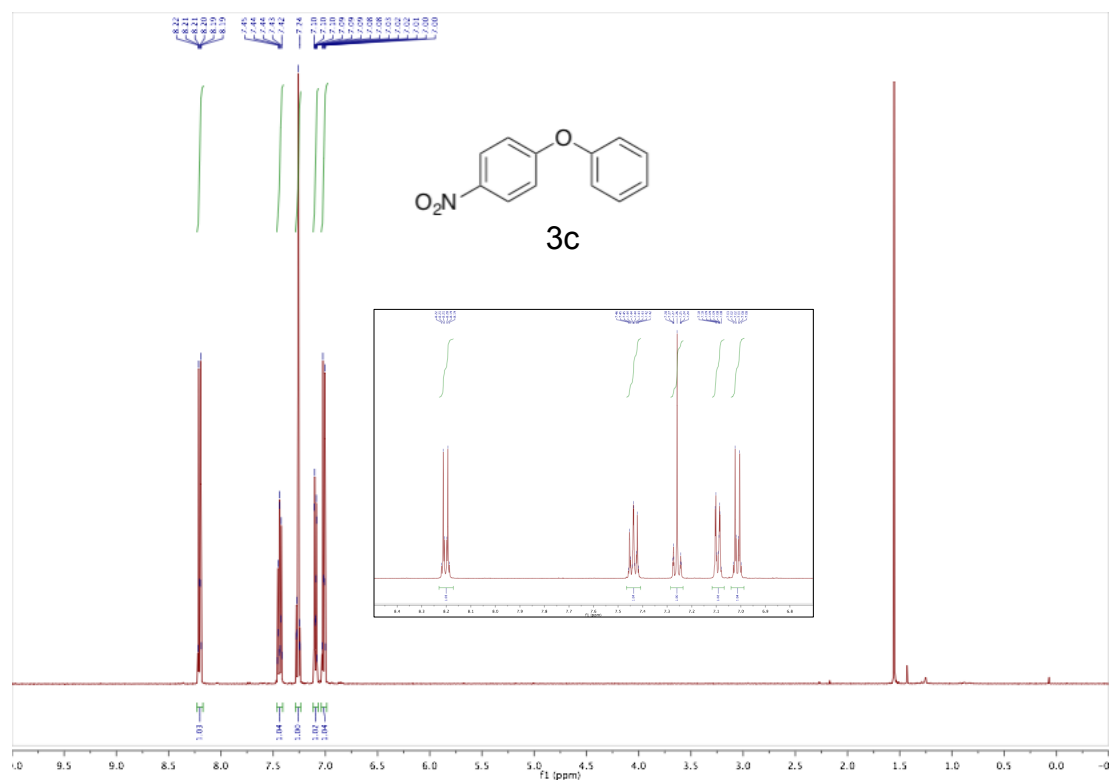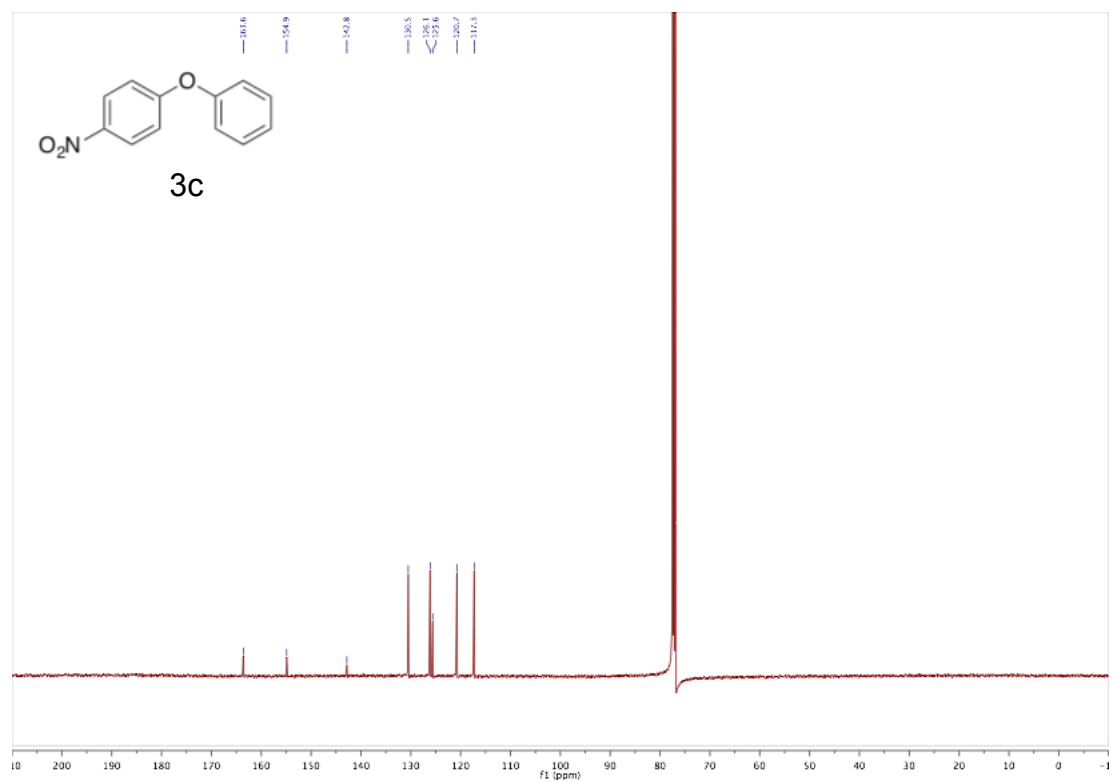



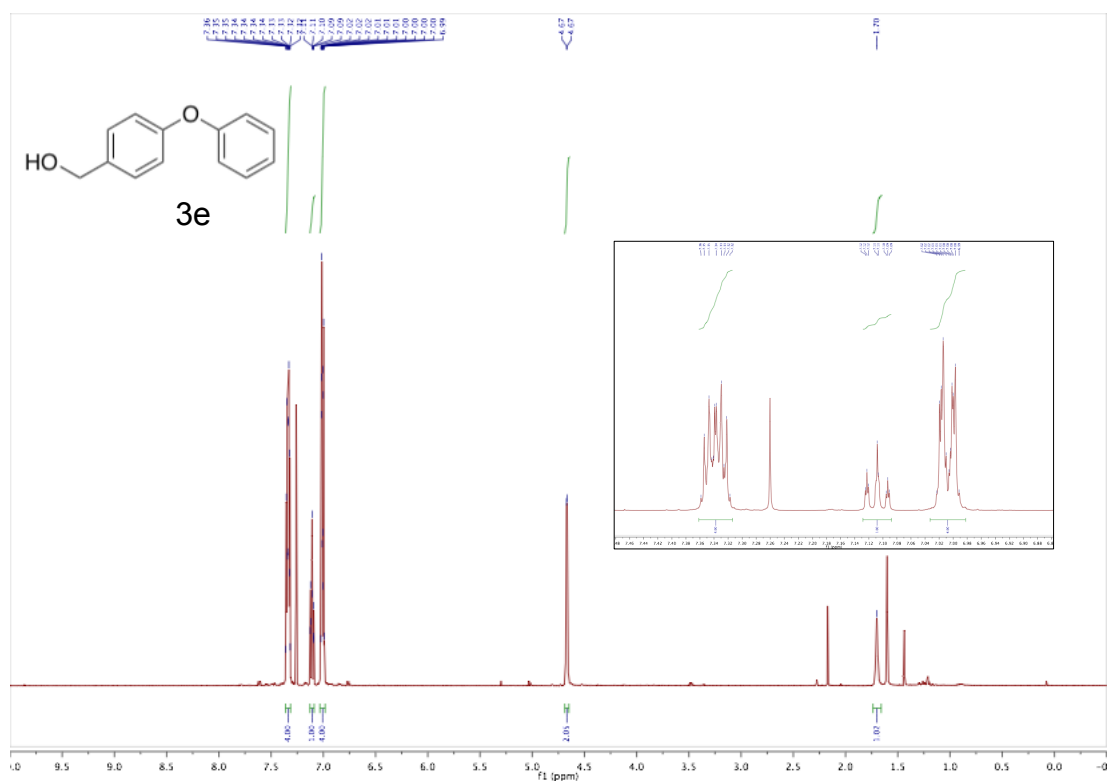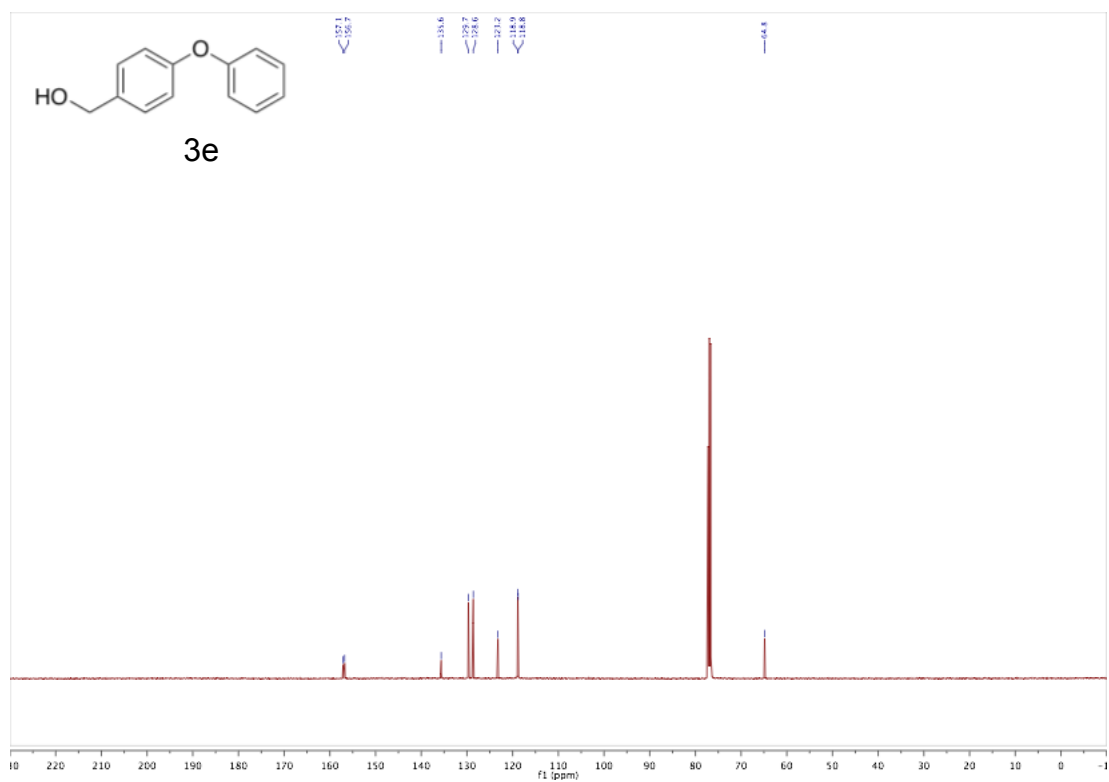

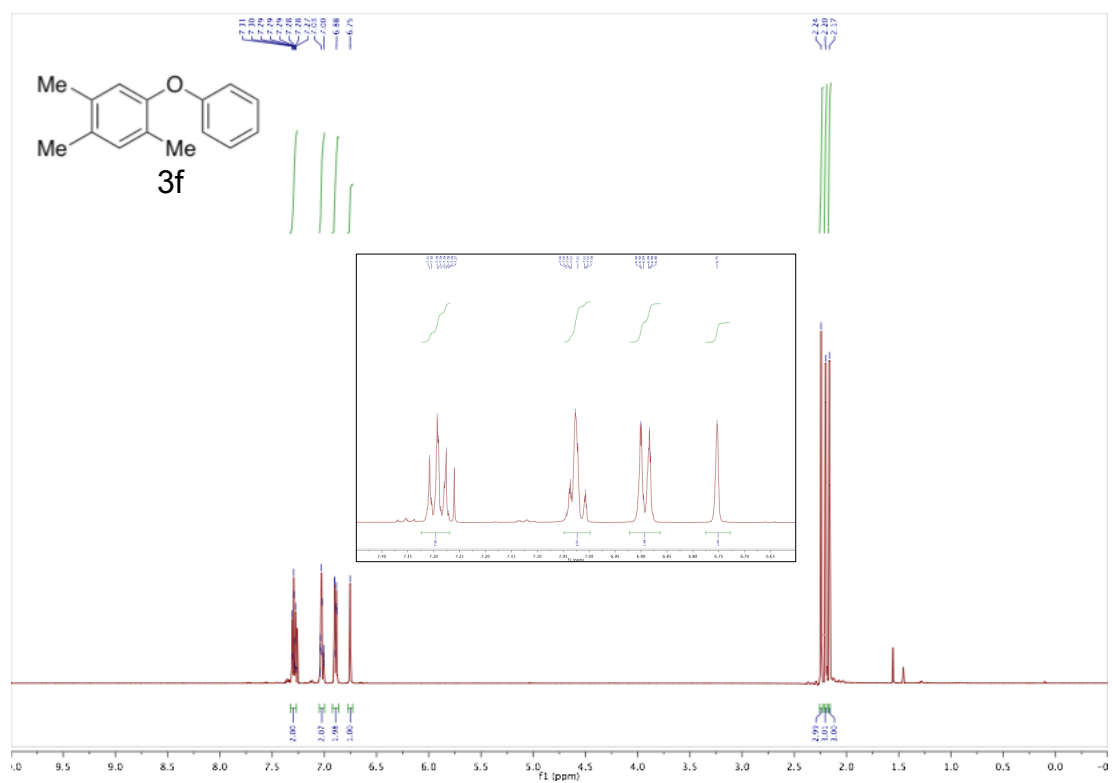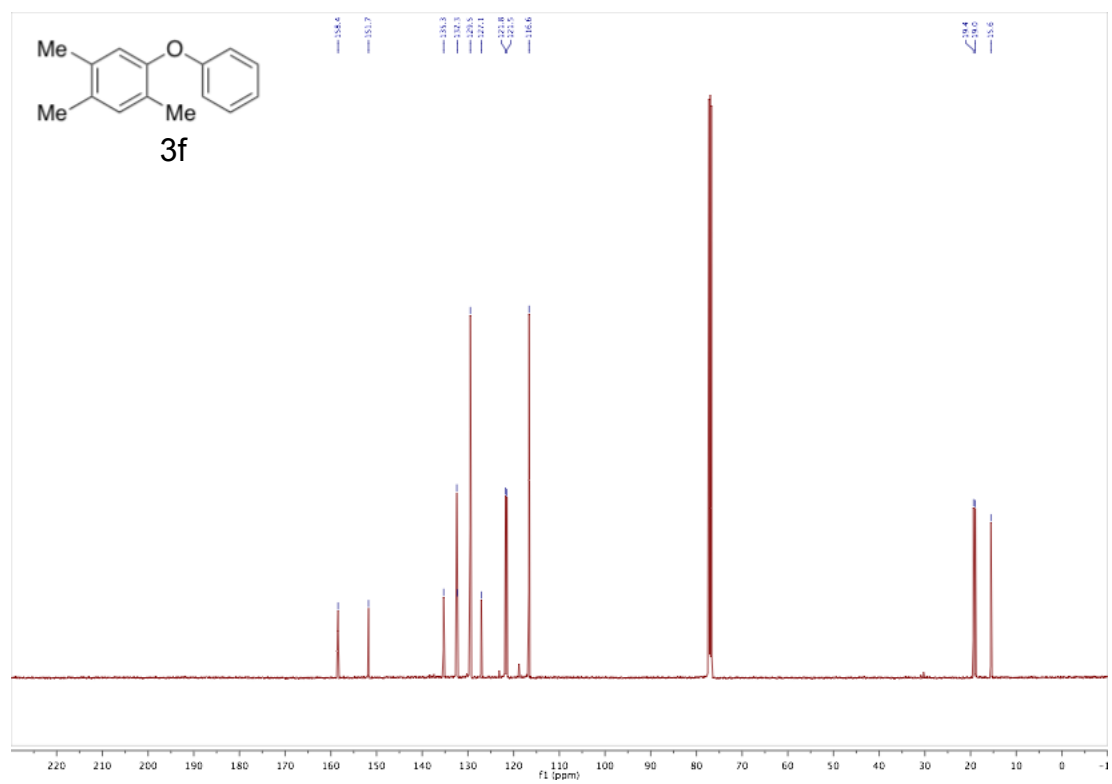

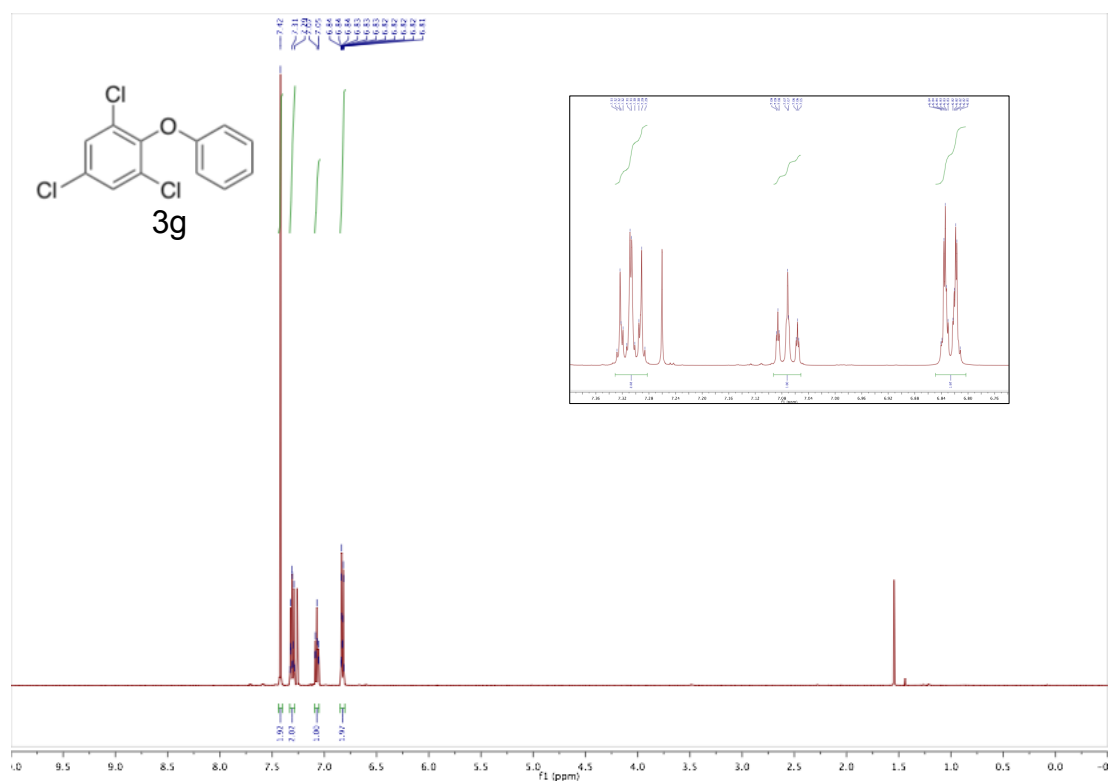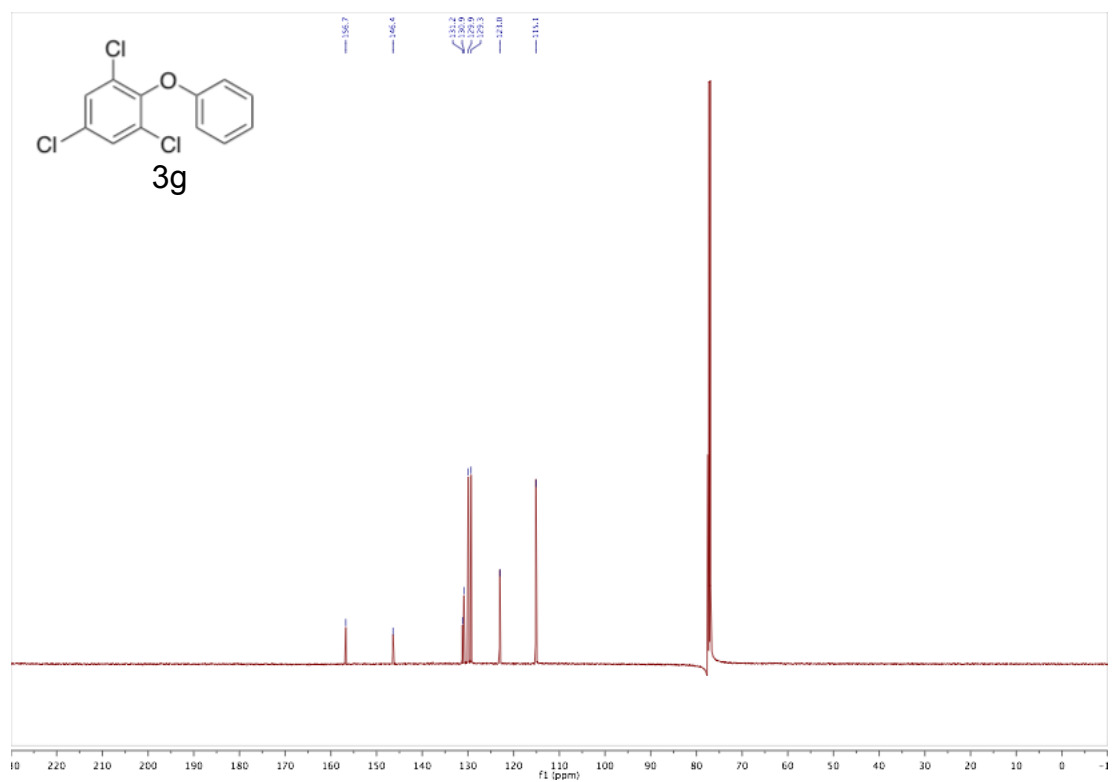

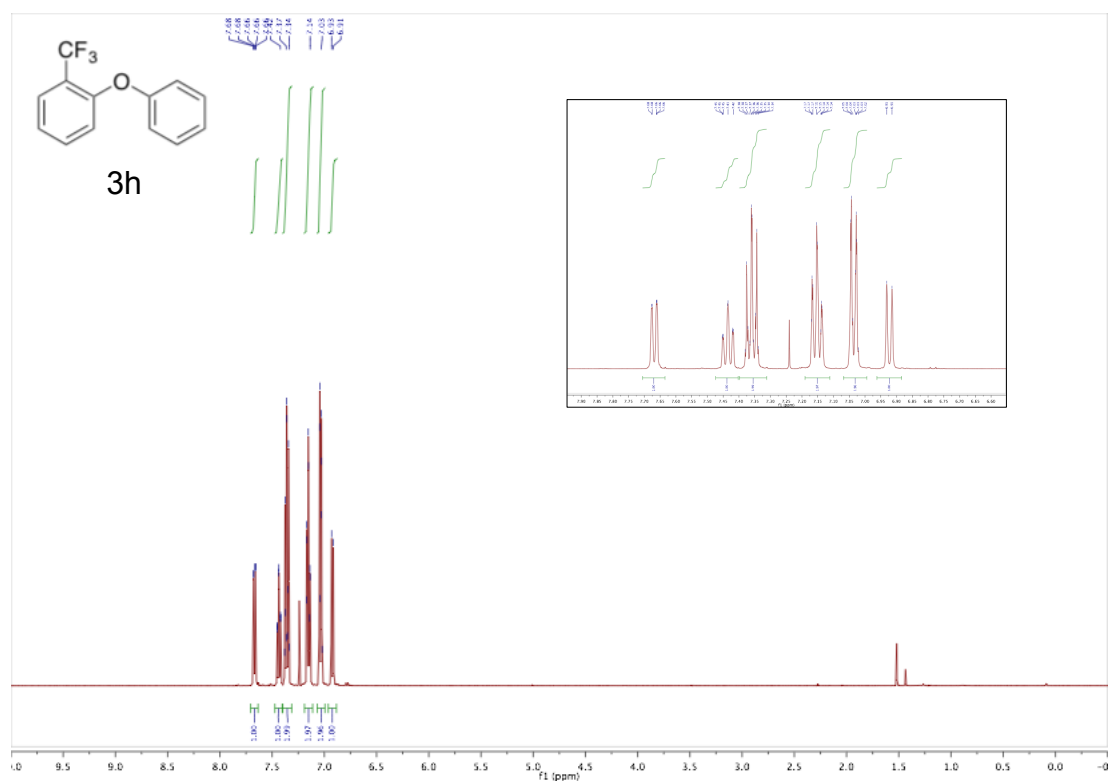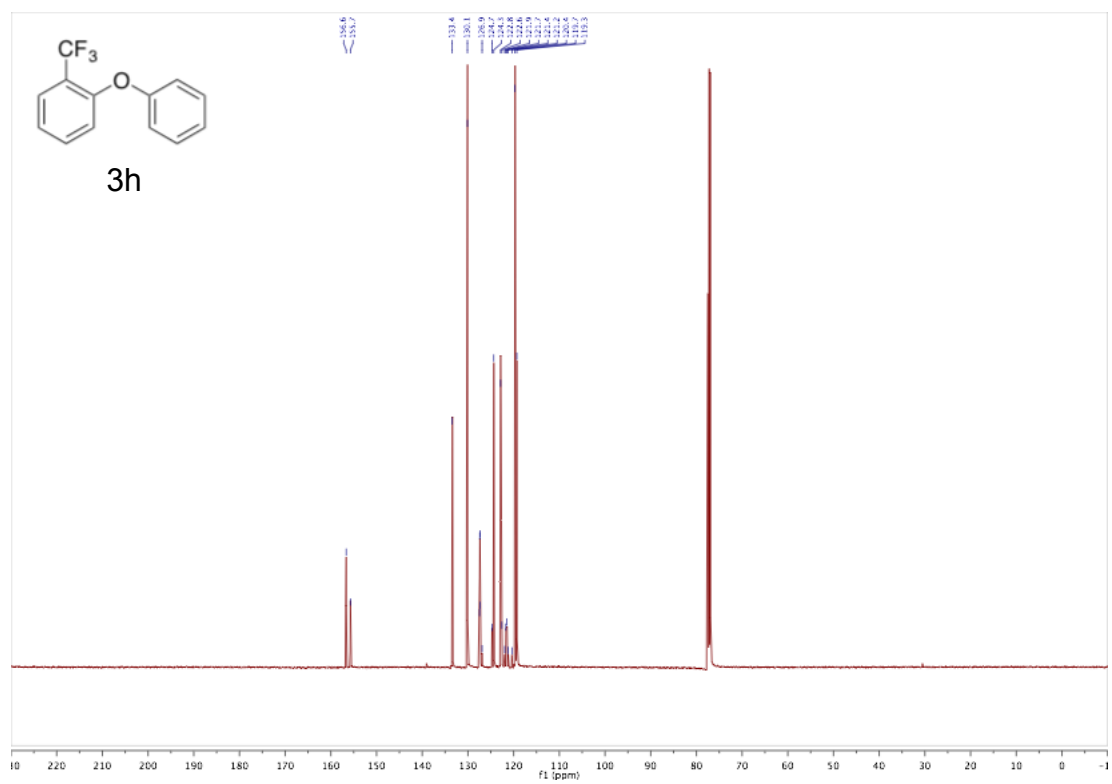

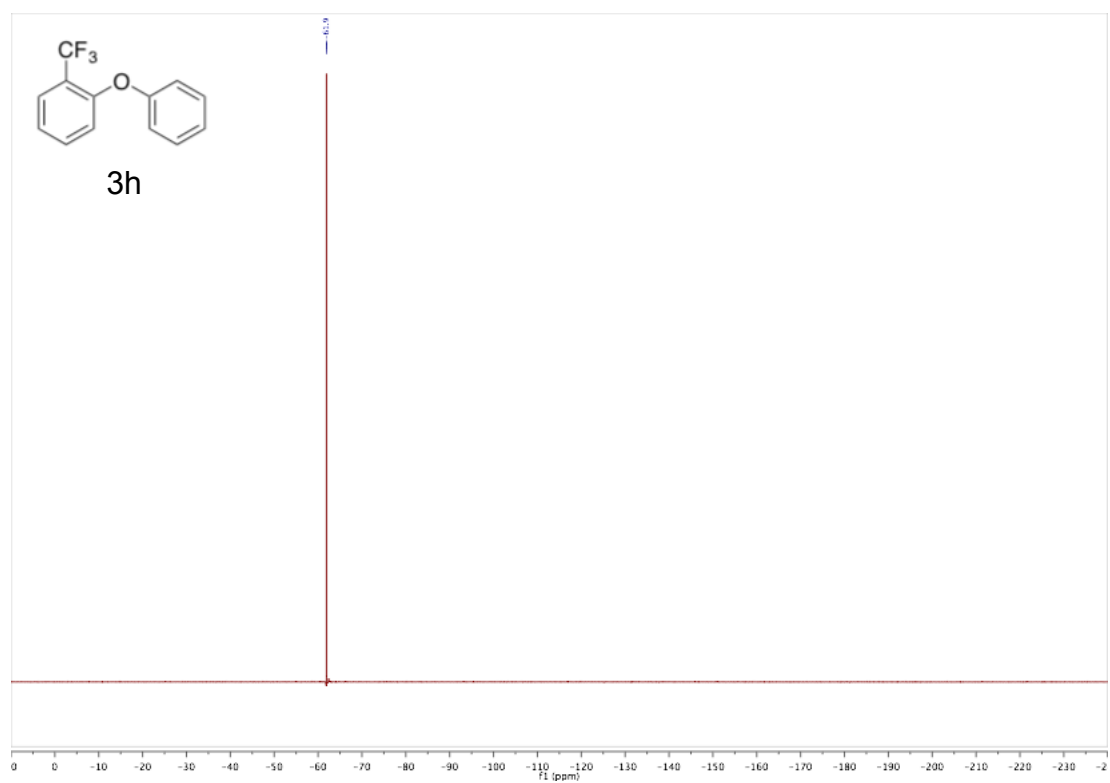



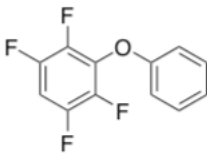

12



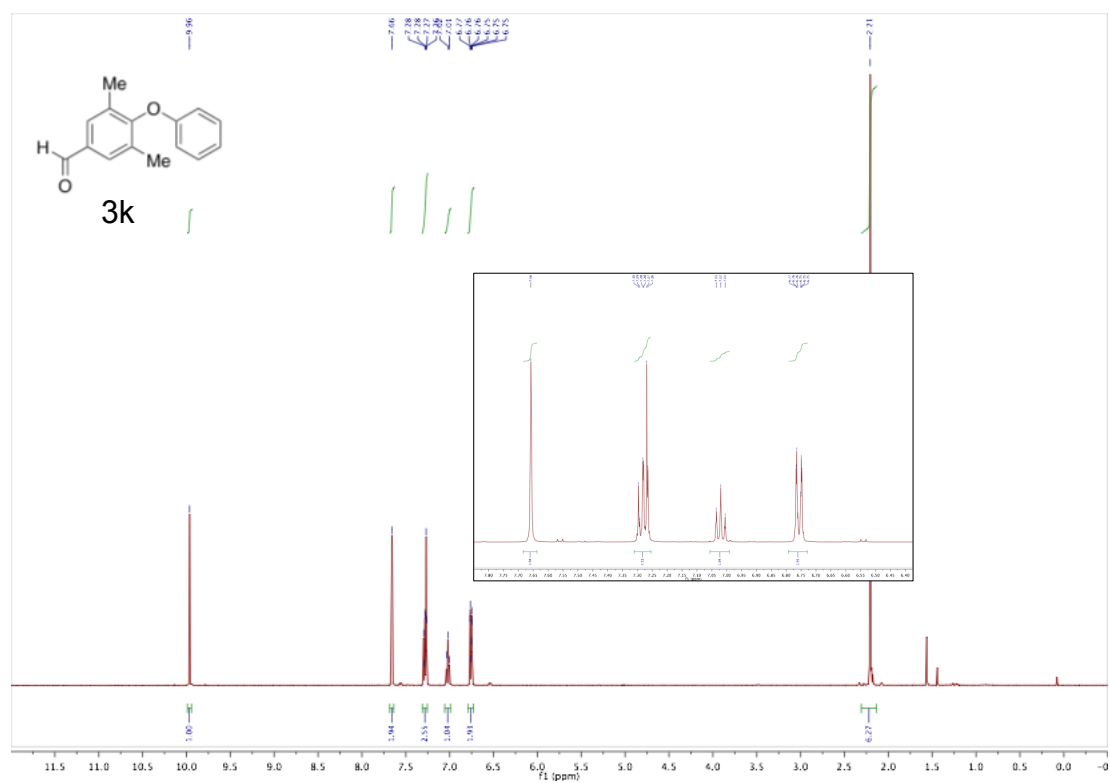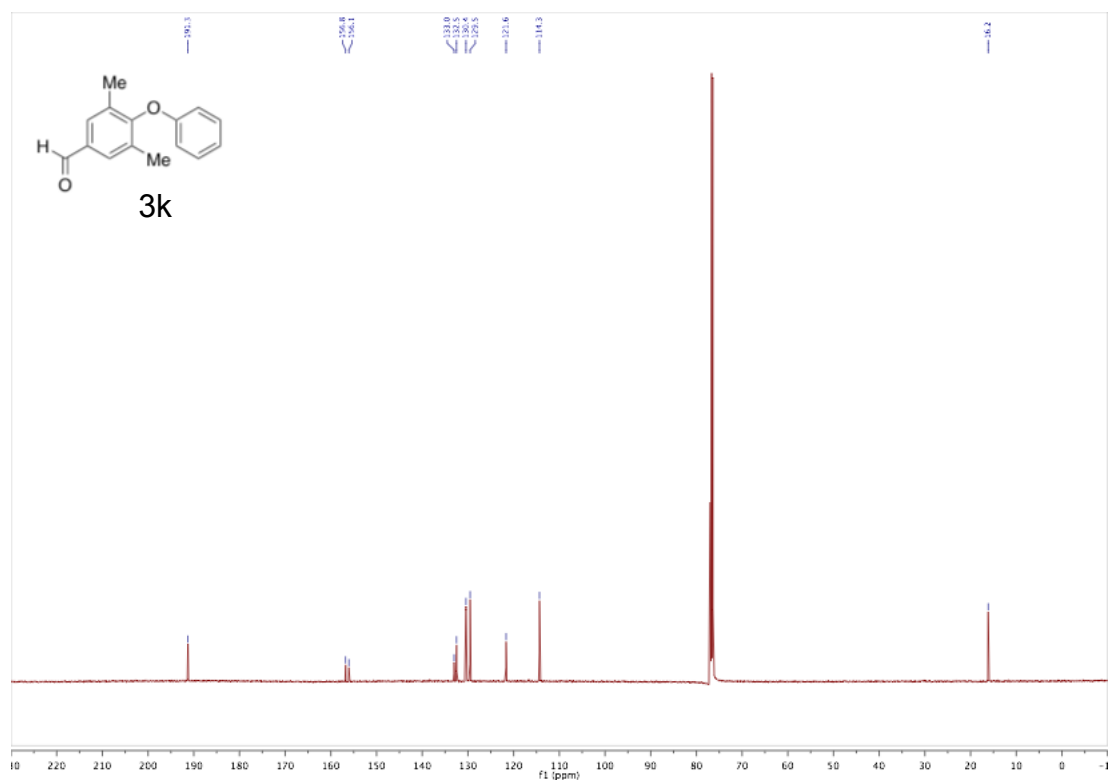

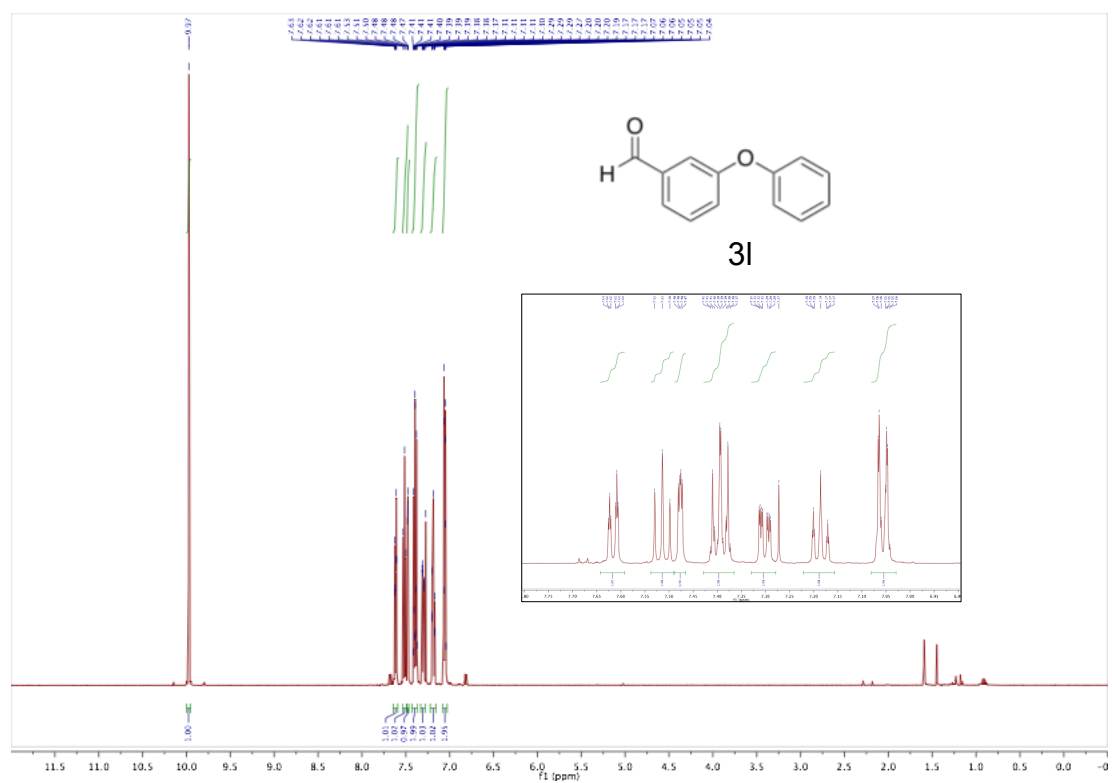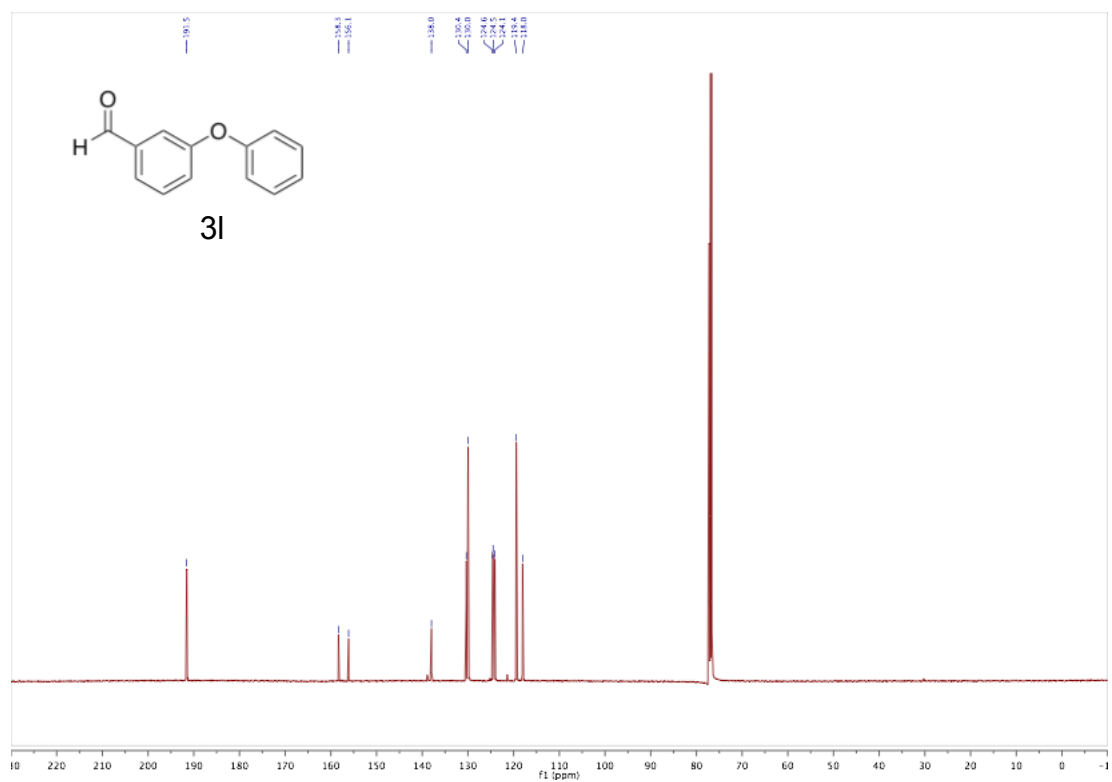

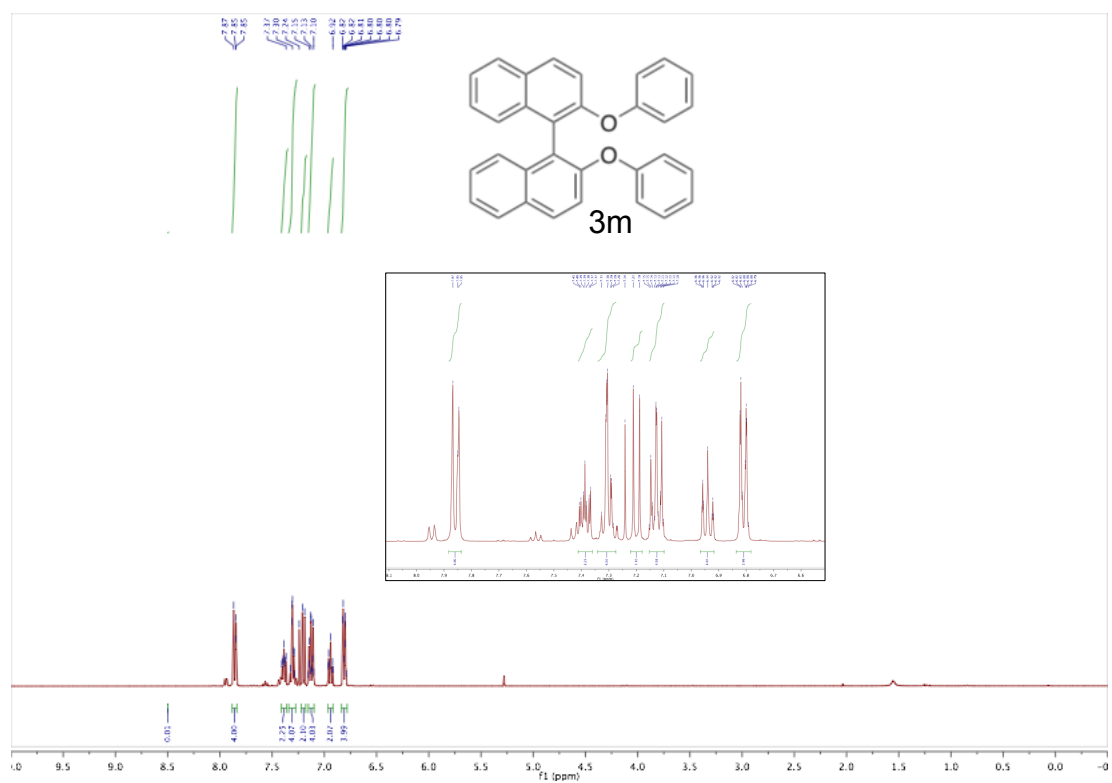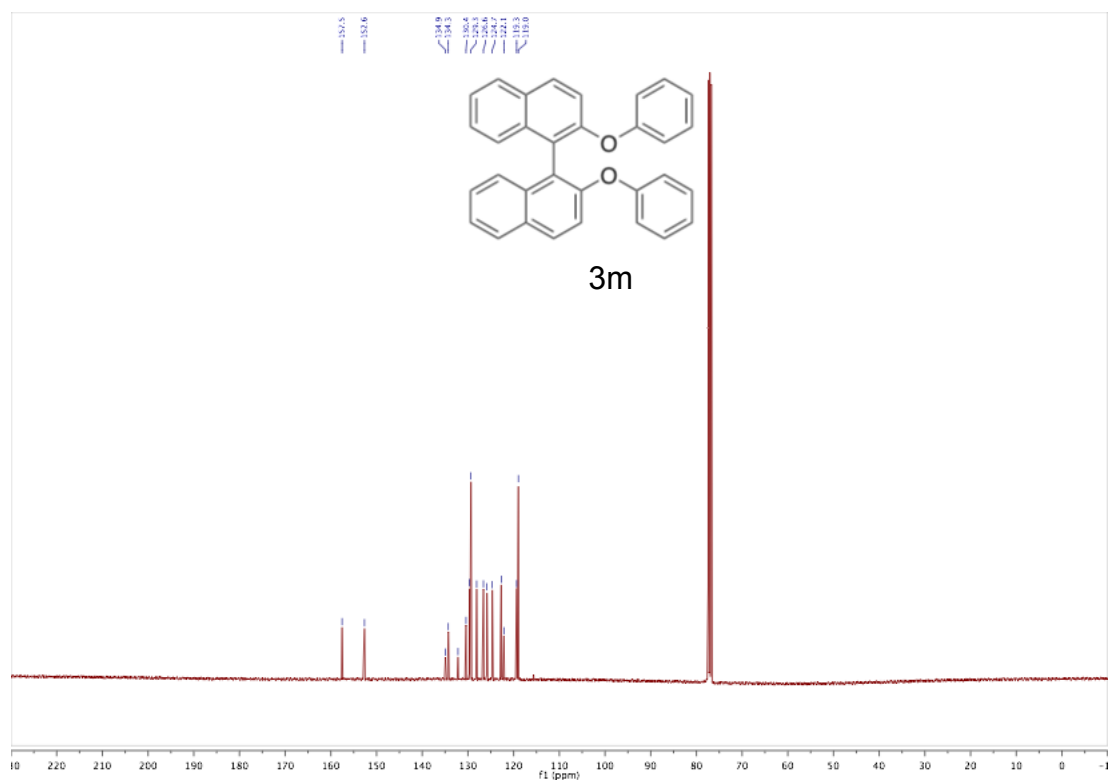



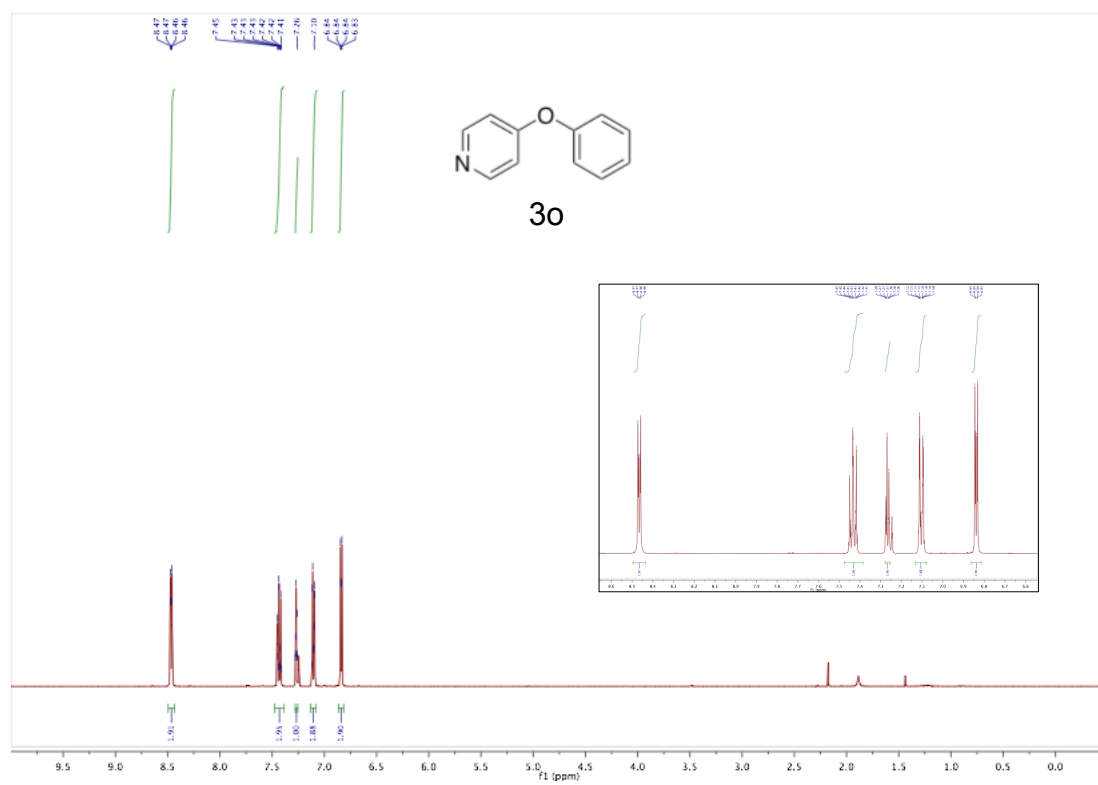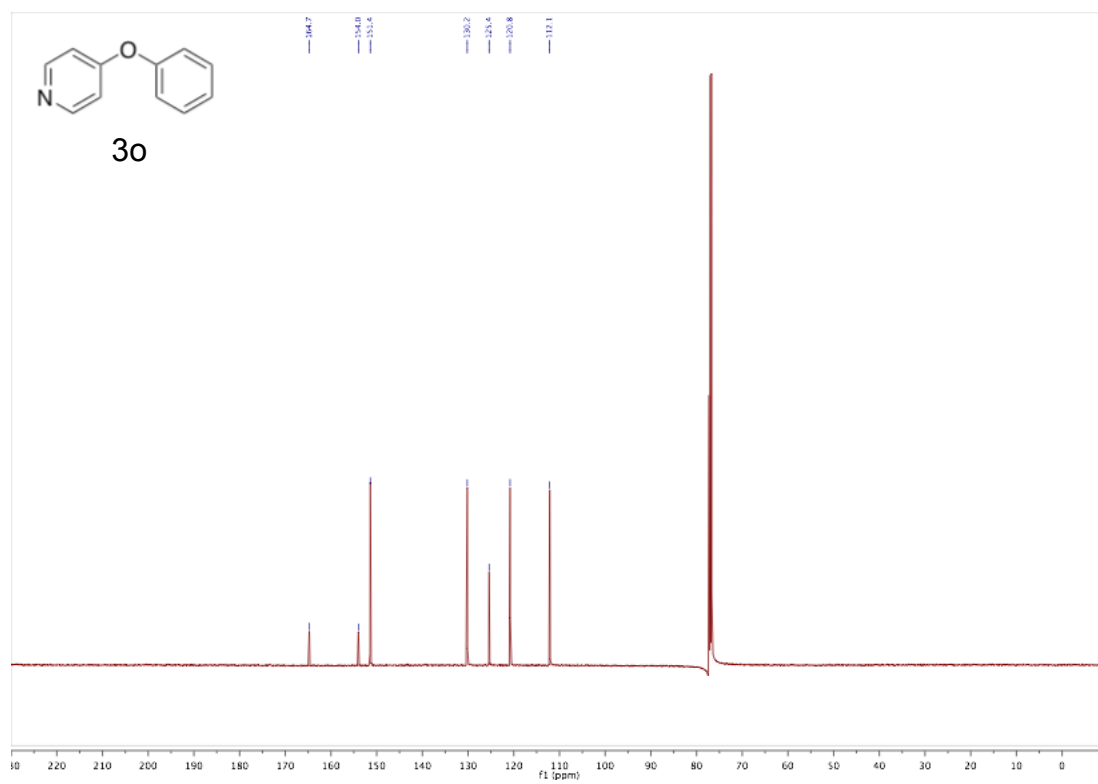



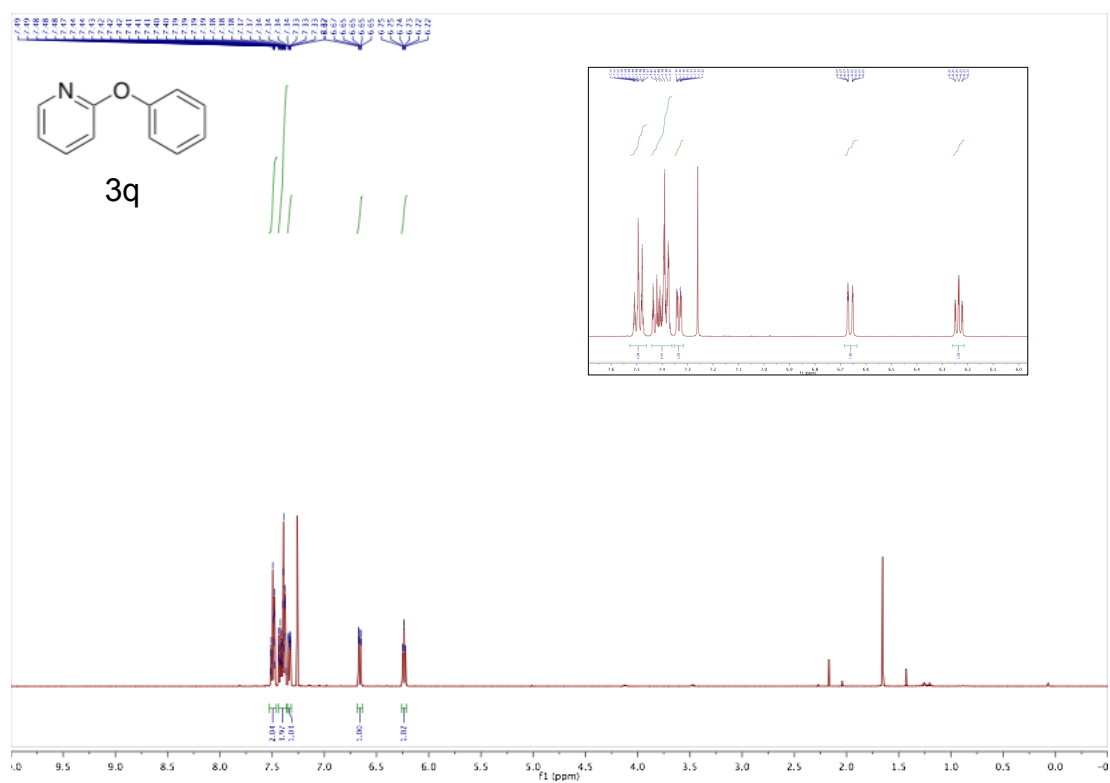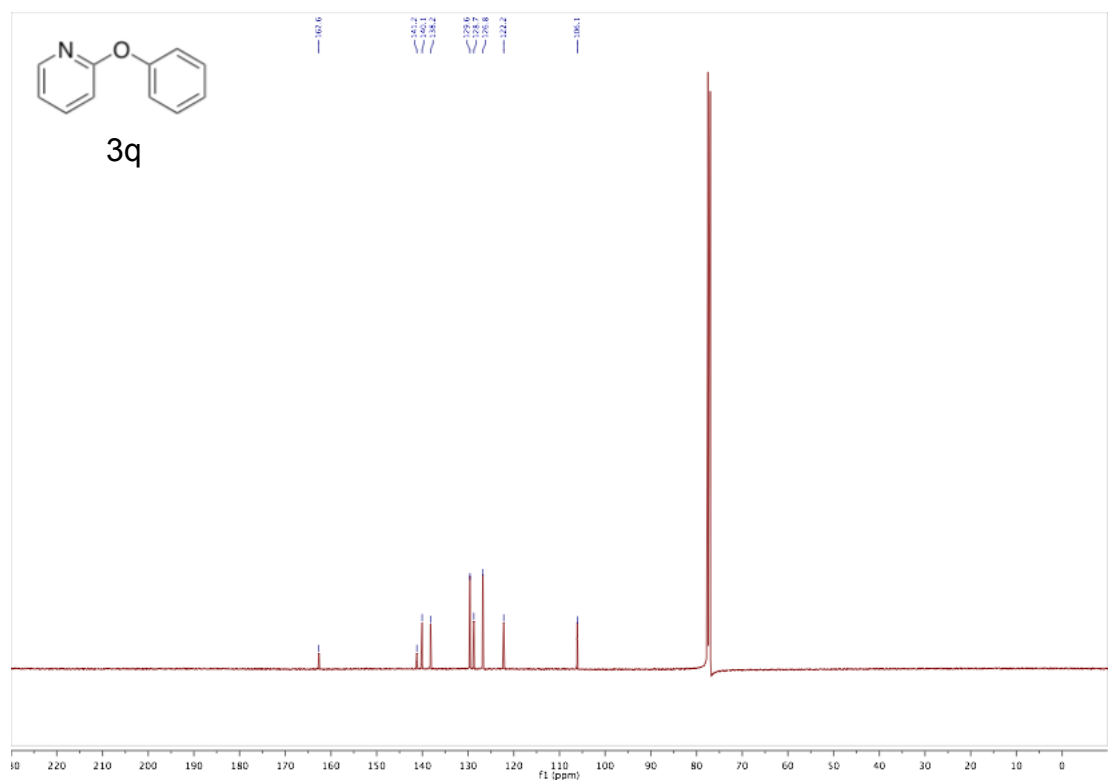

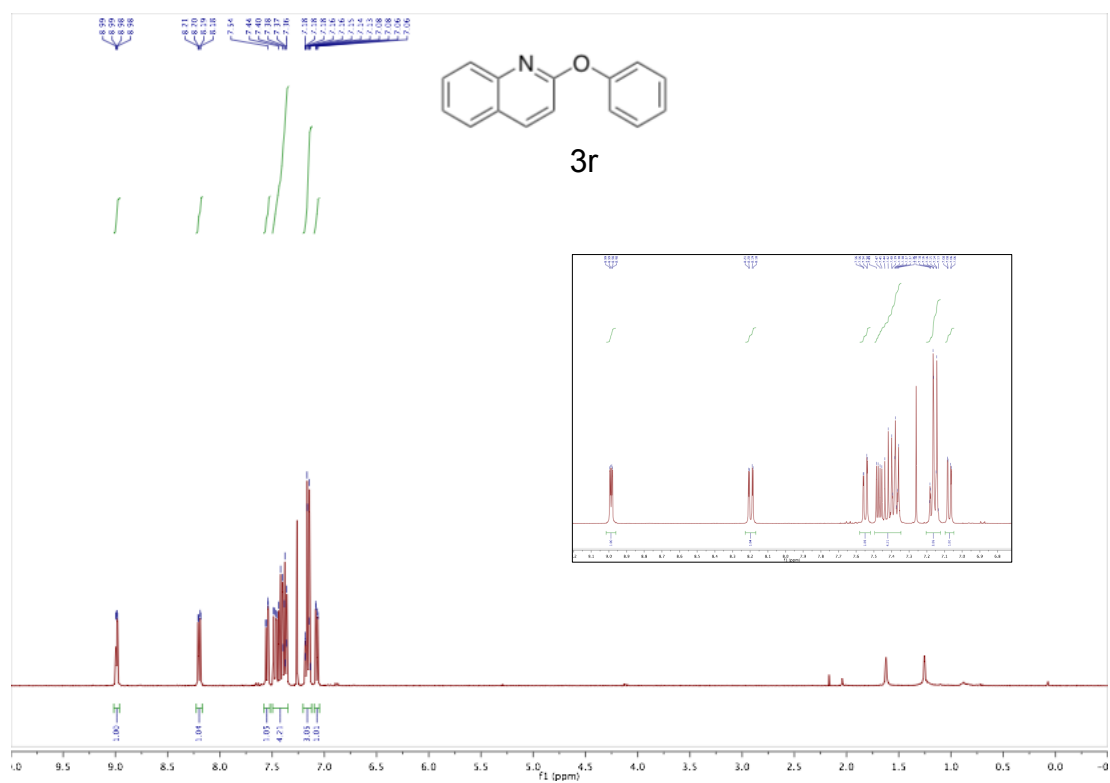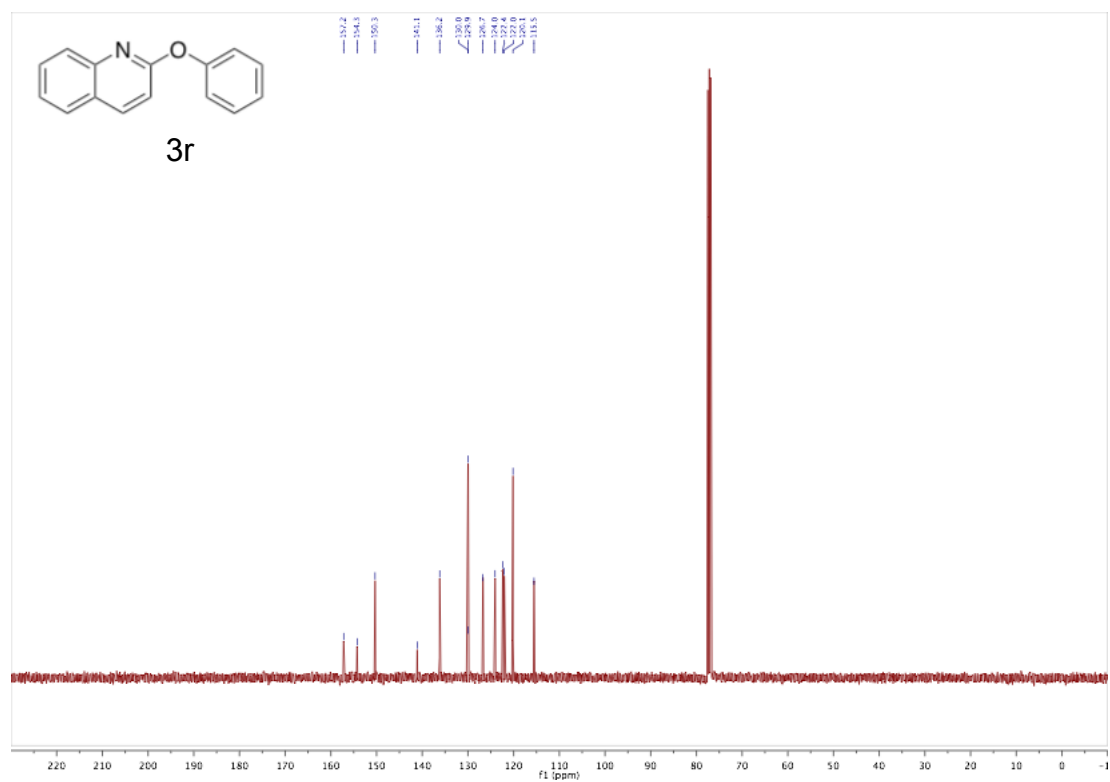

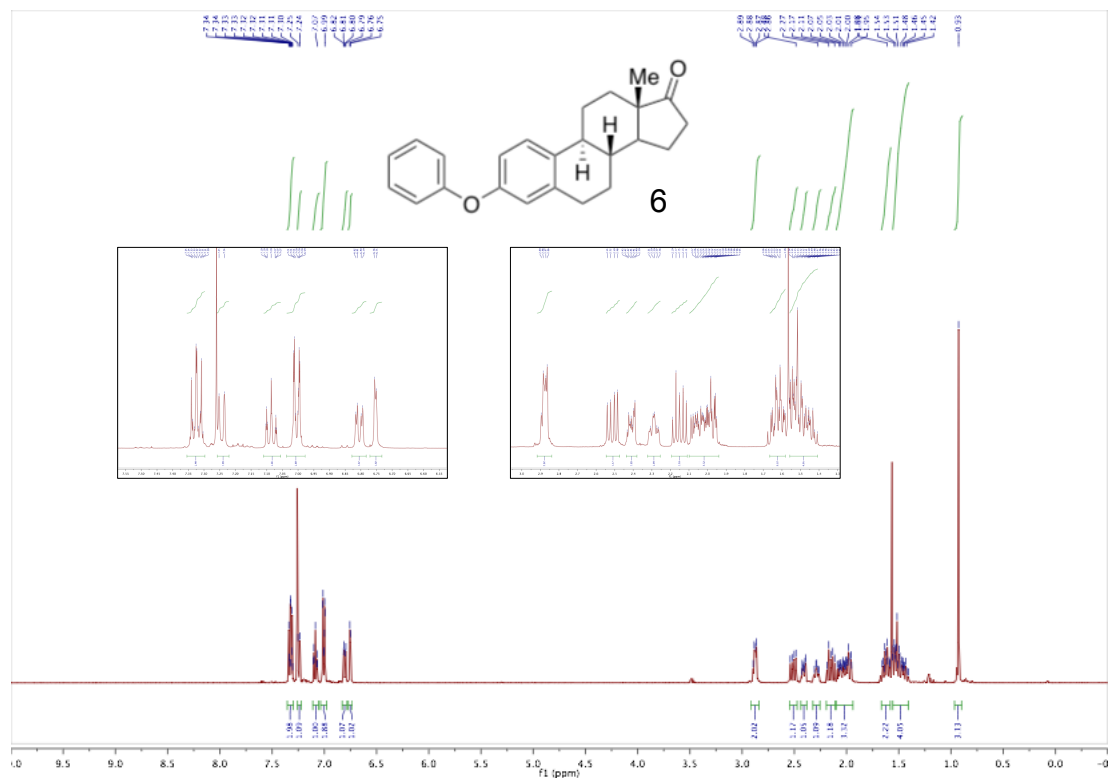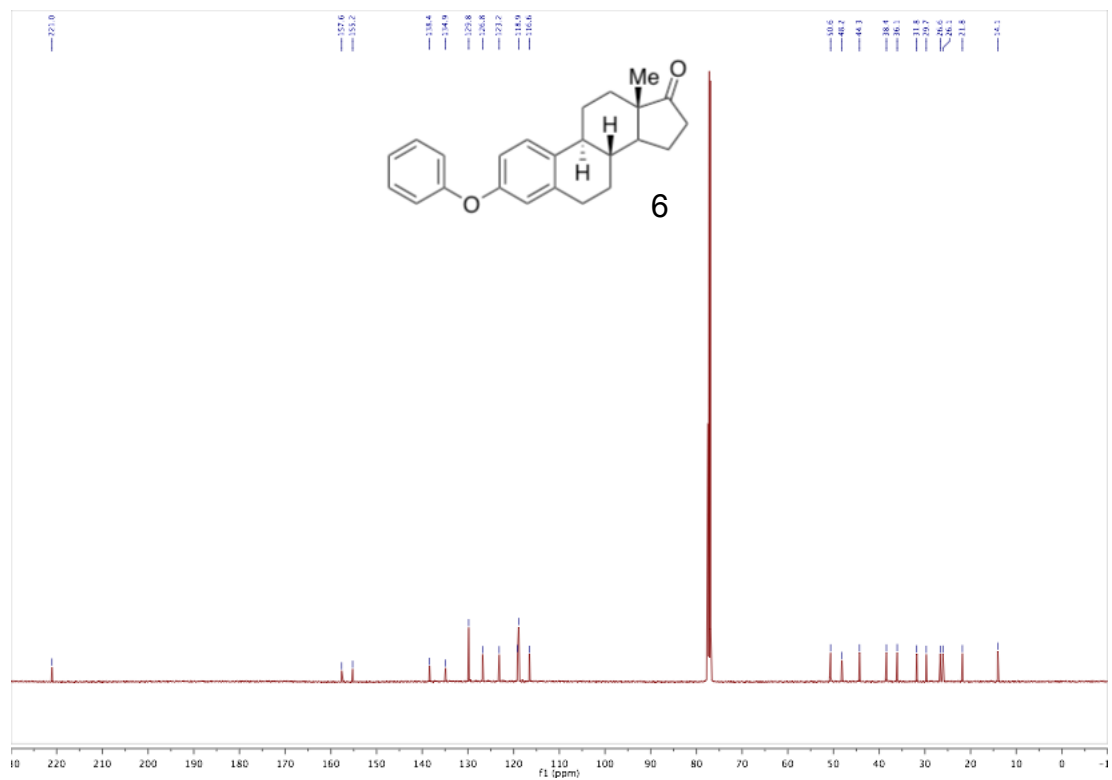



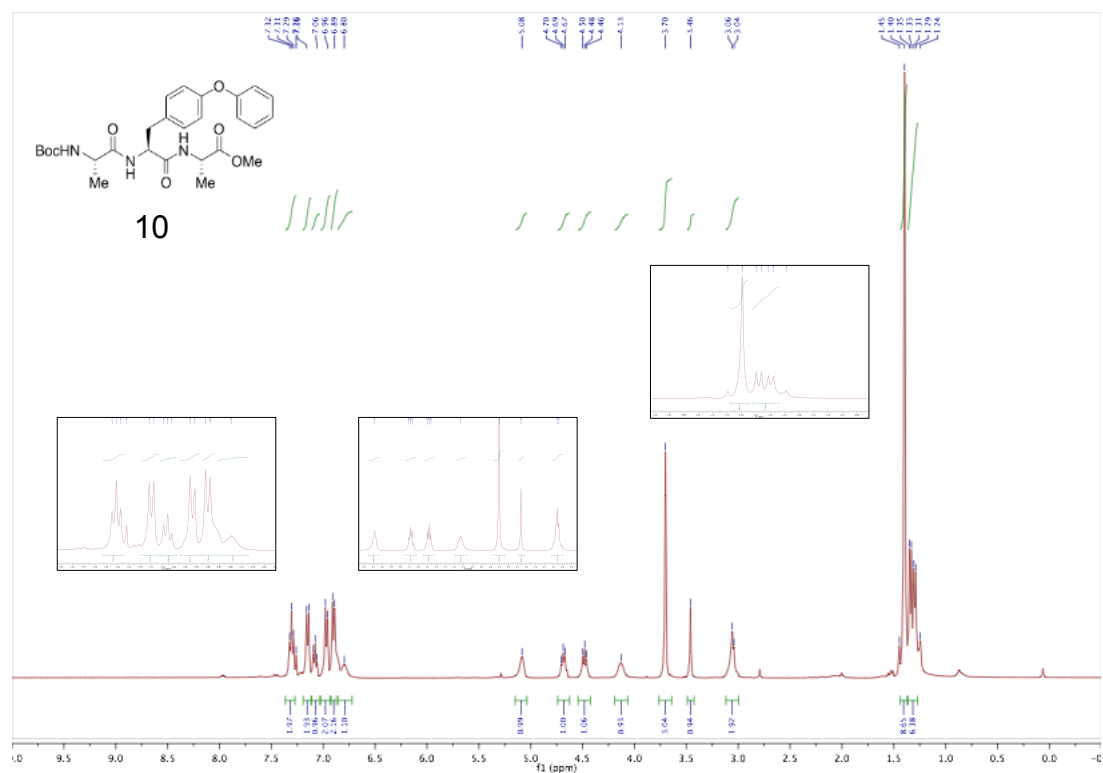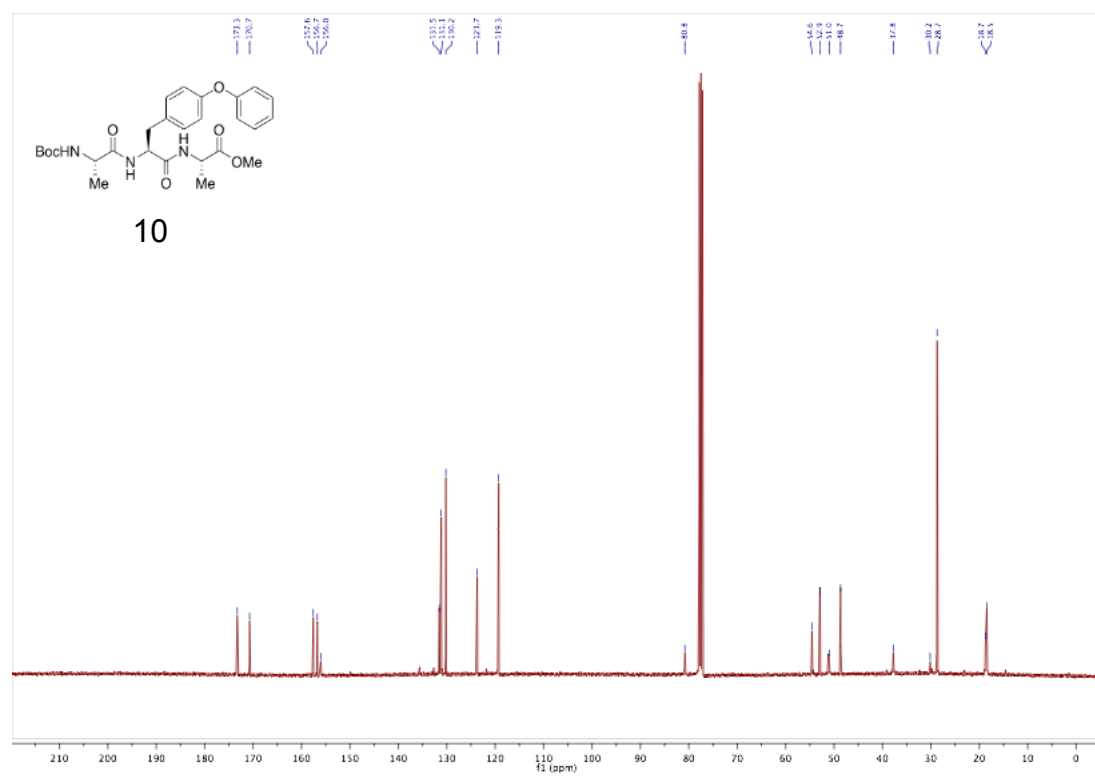

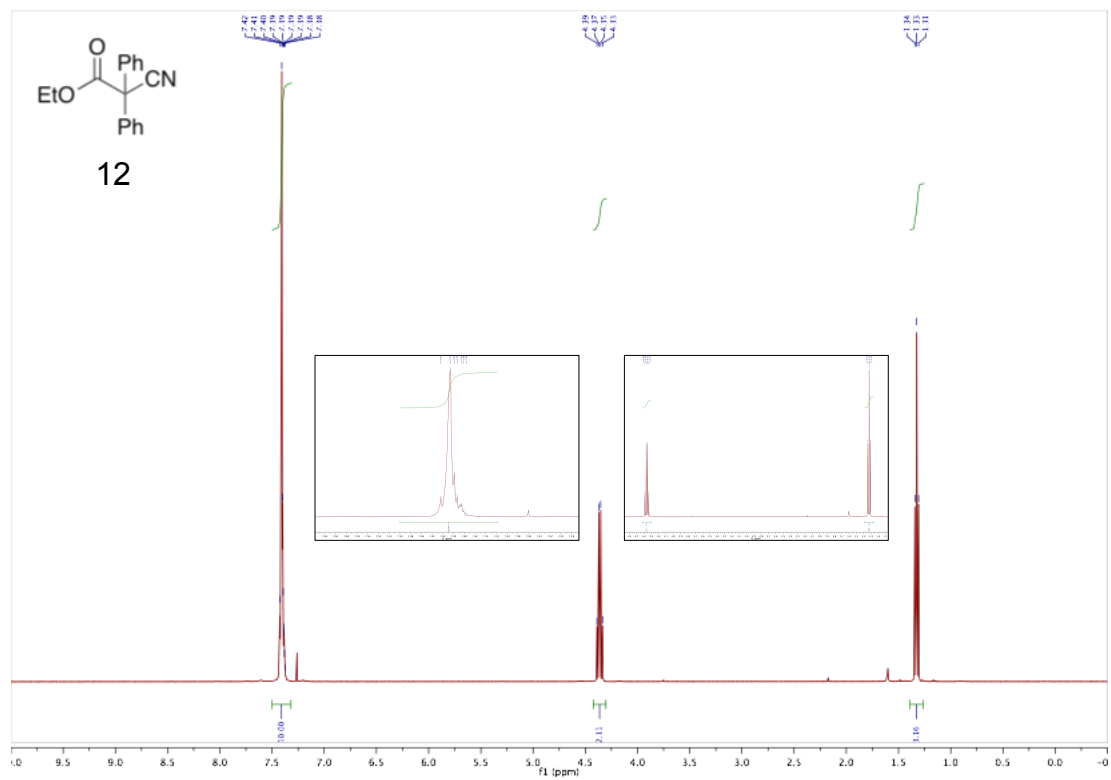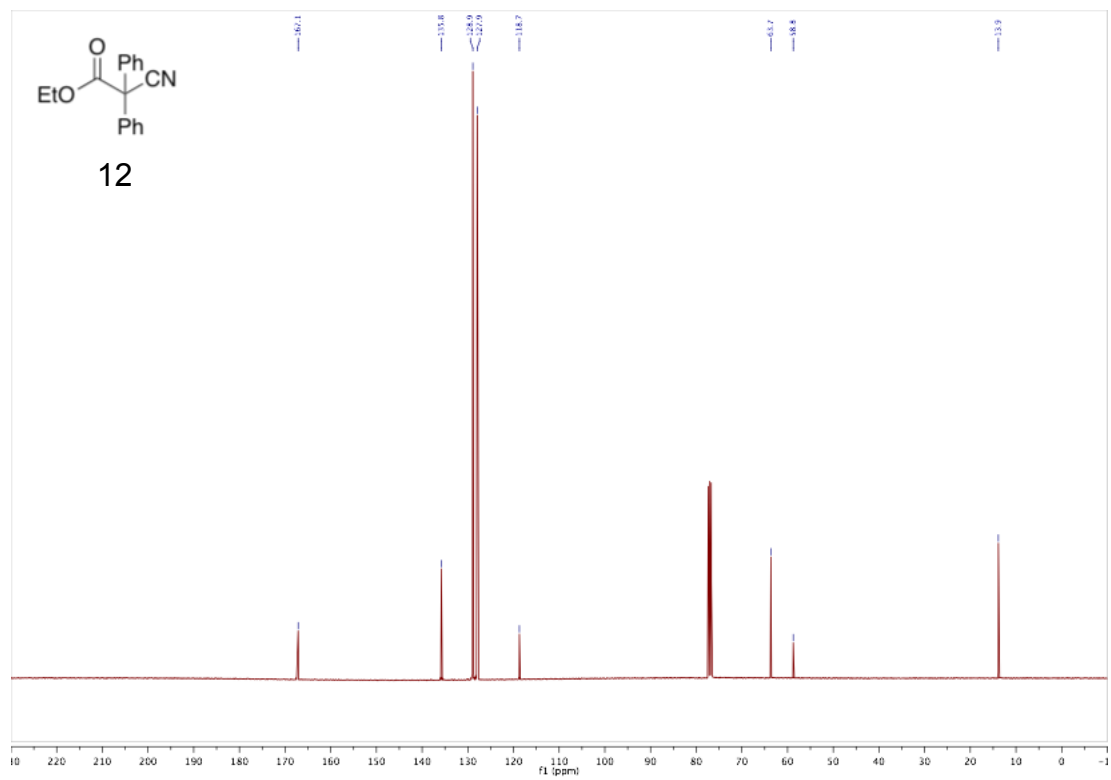

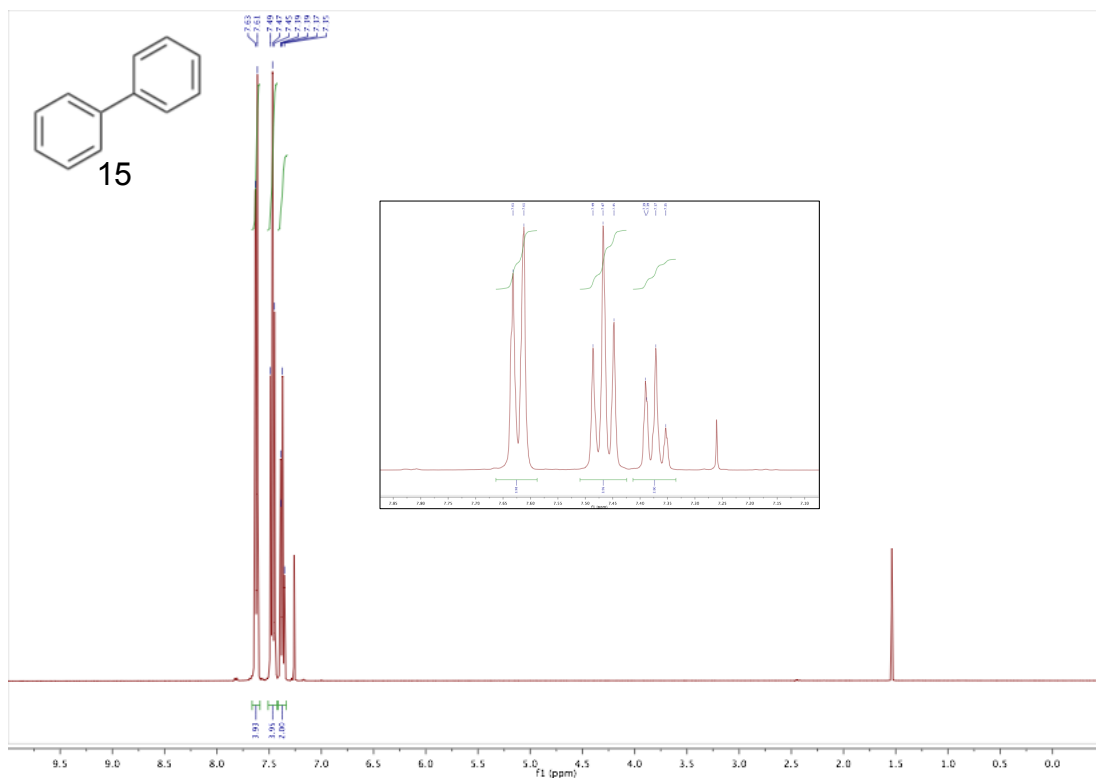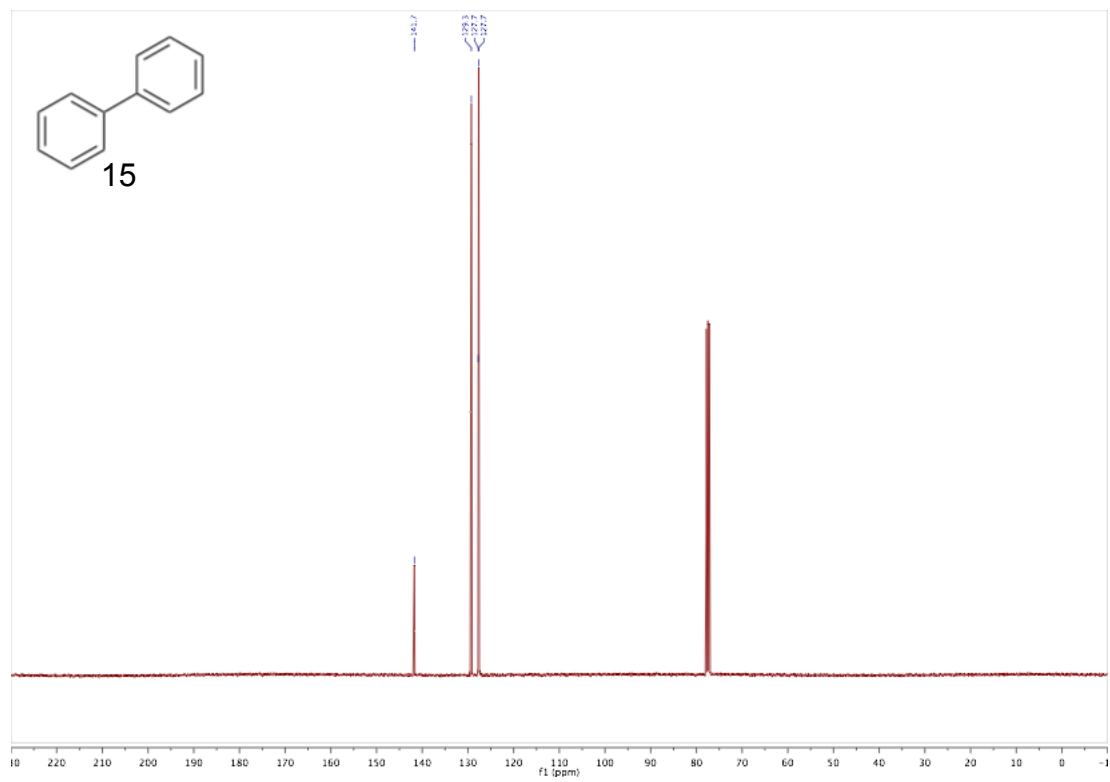

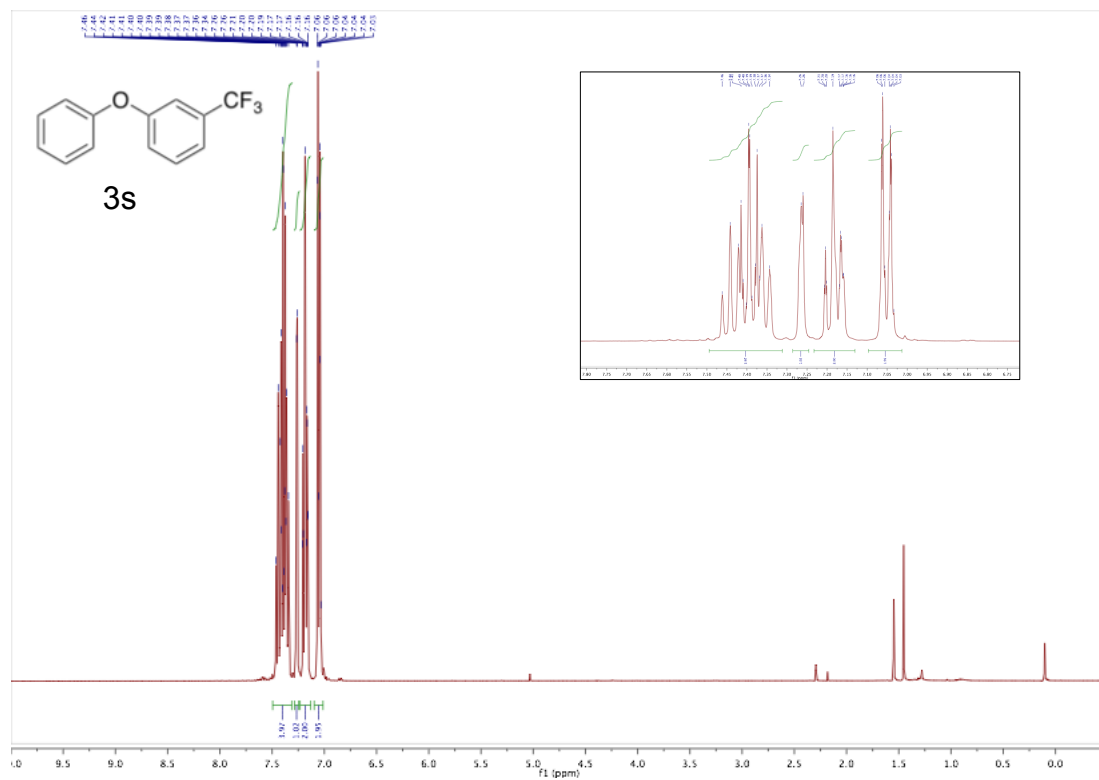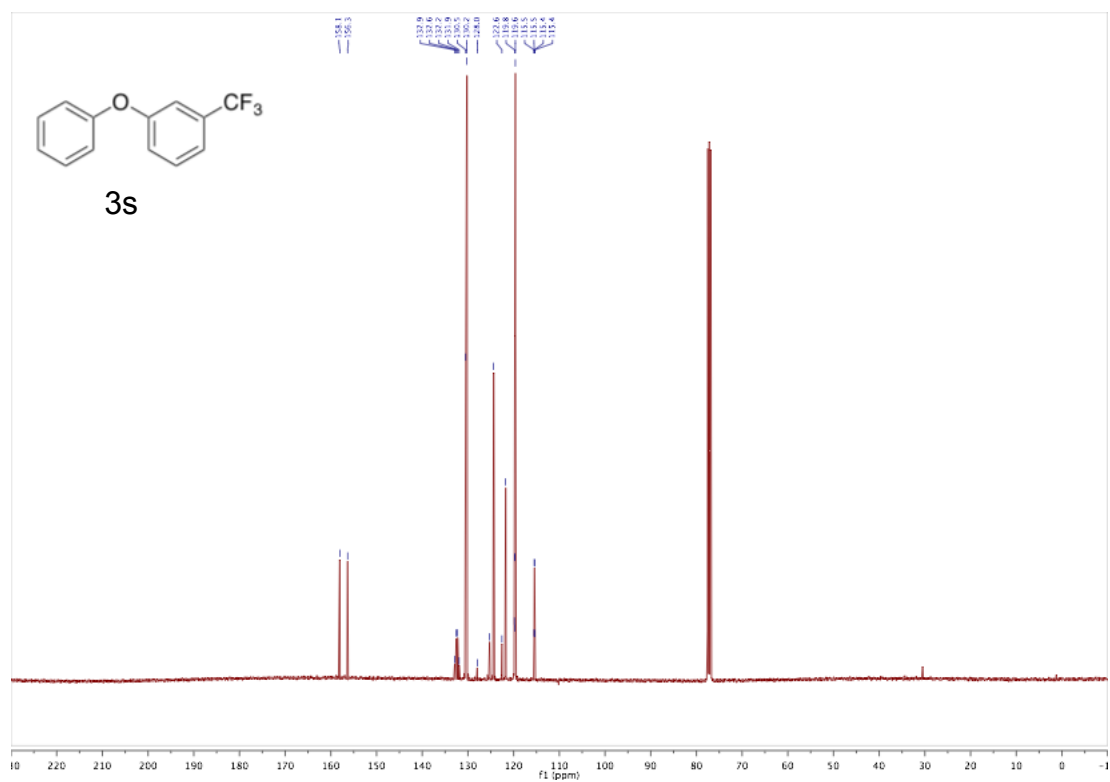

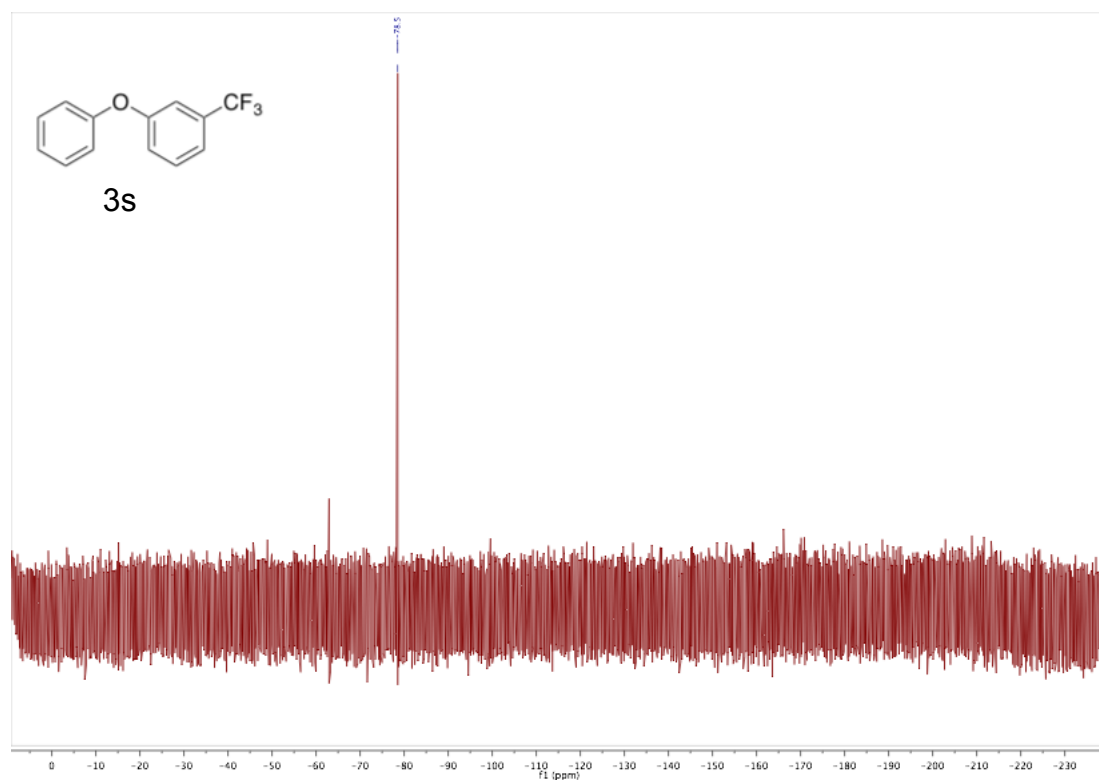

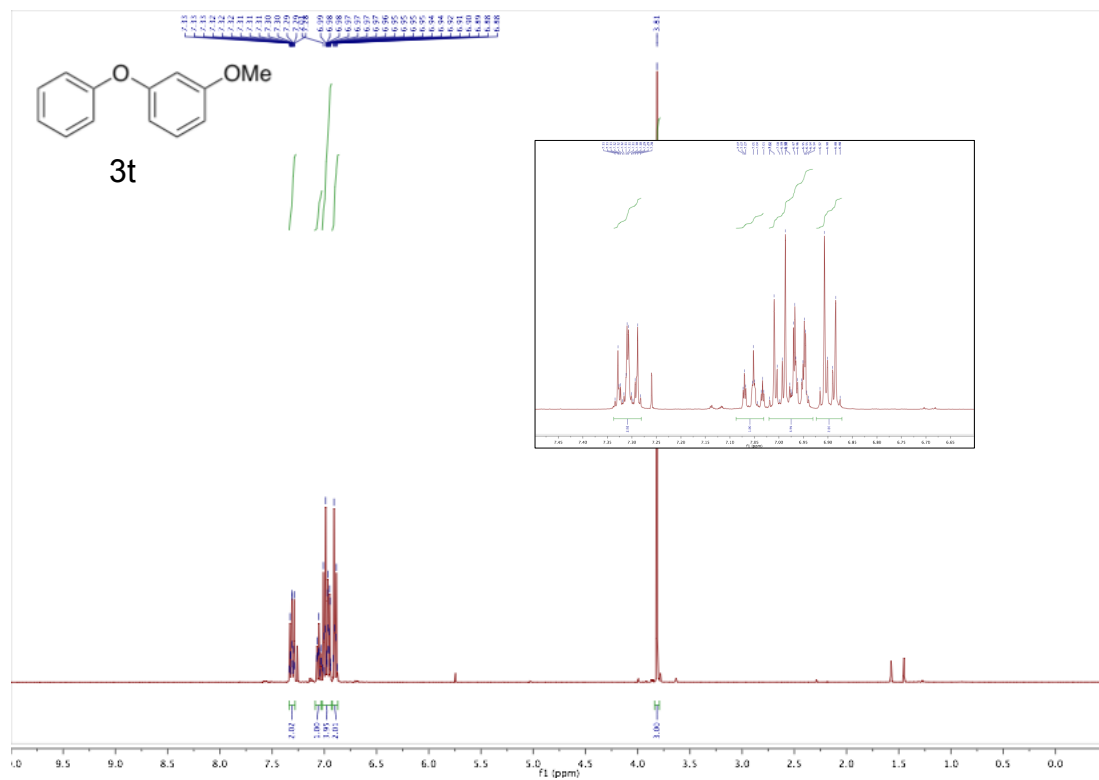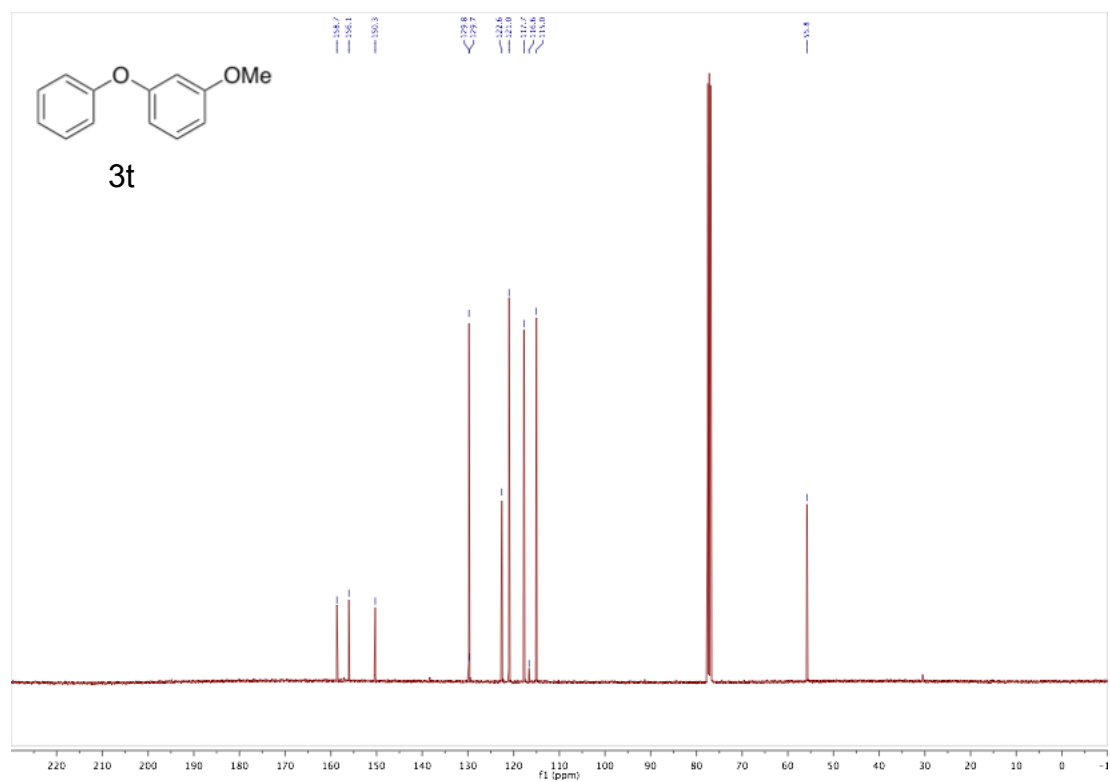

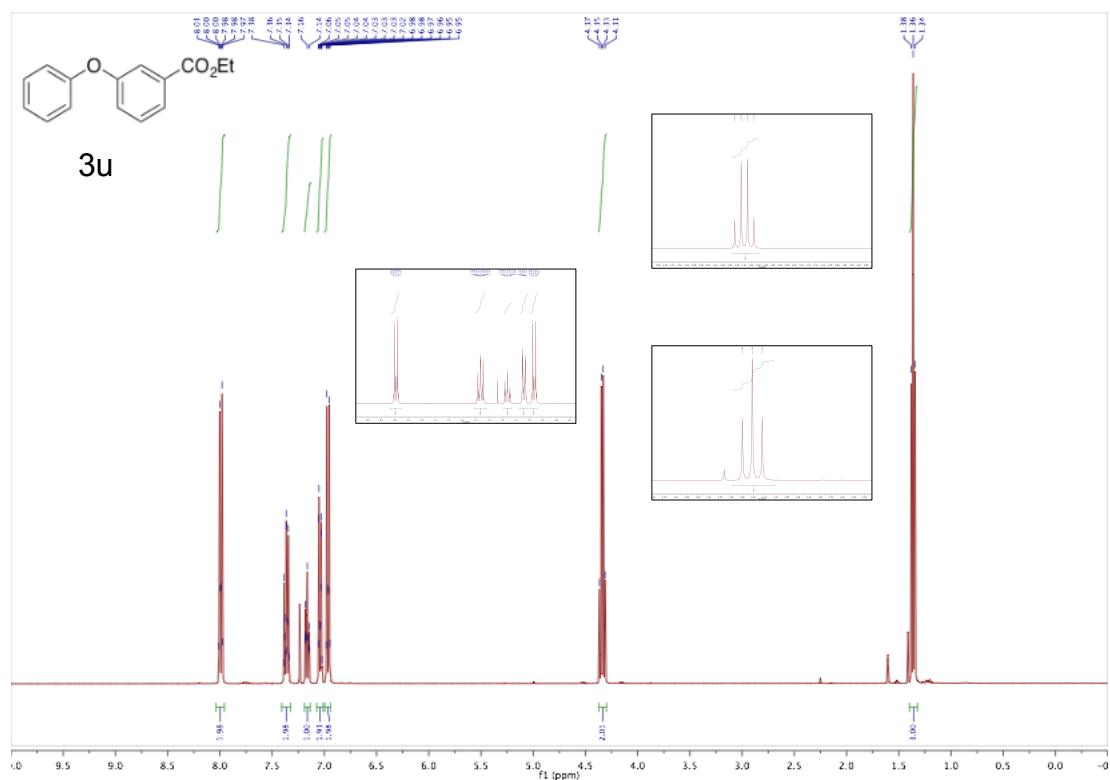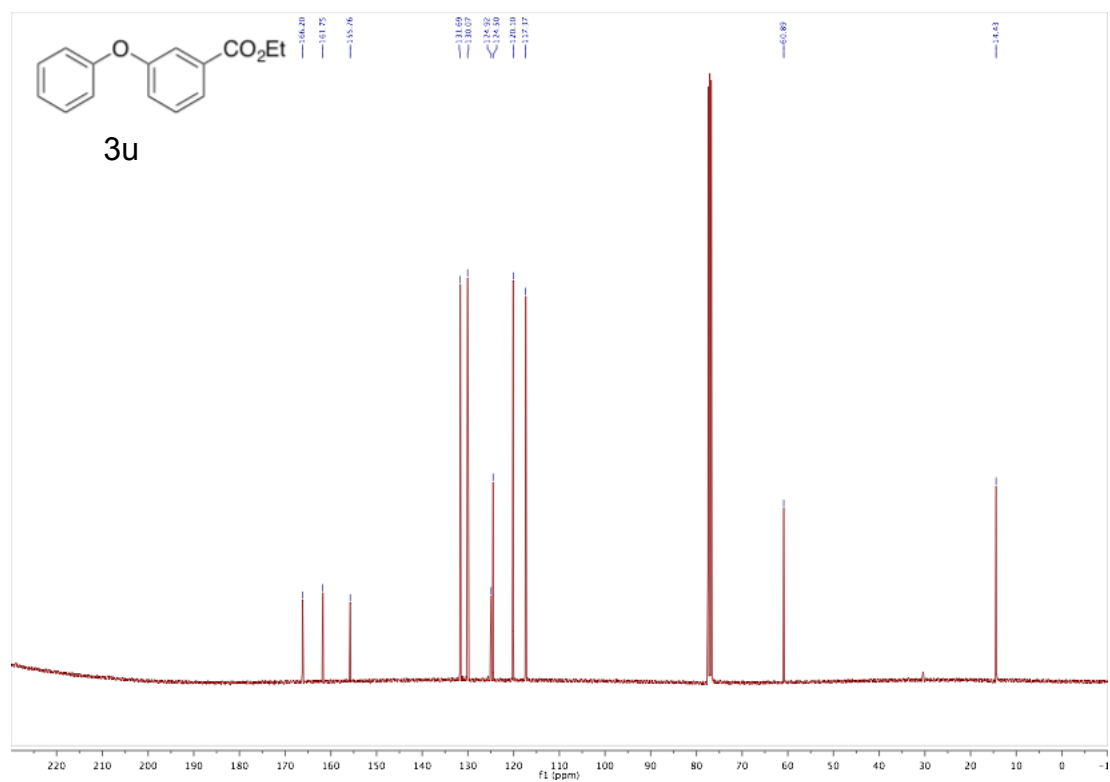



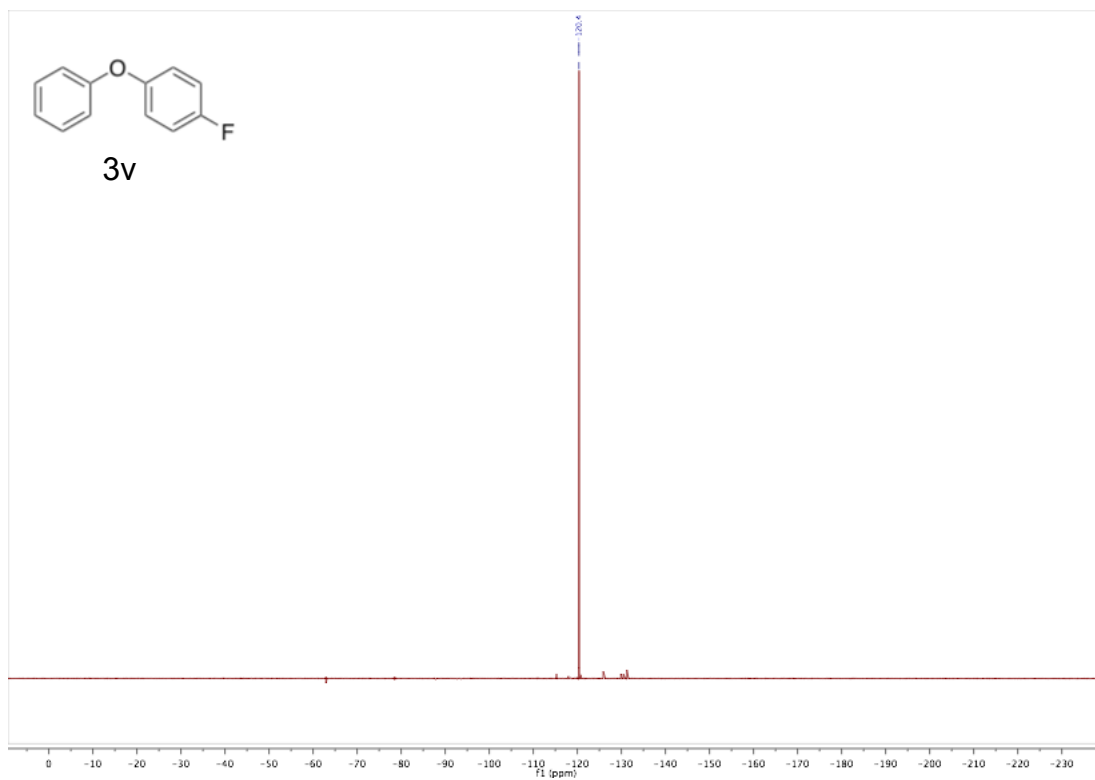

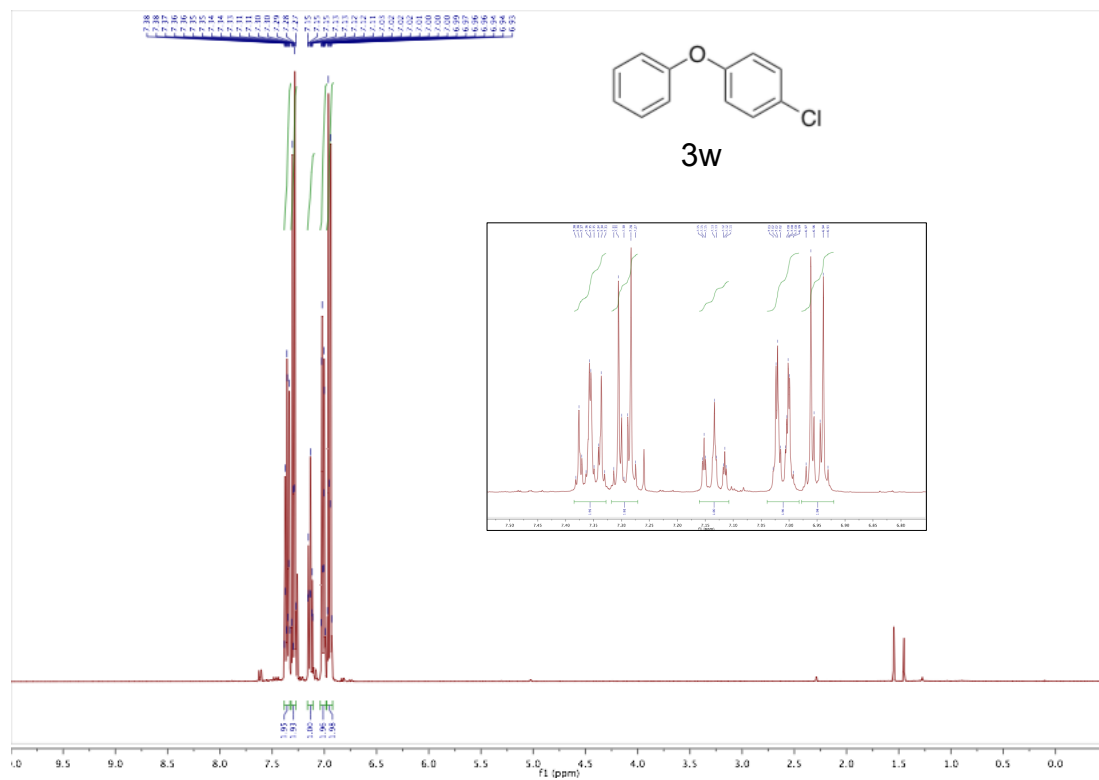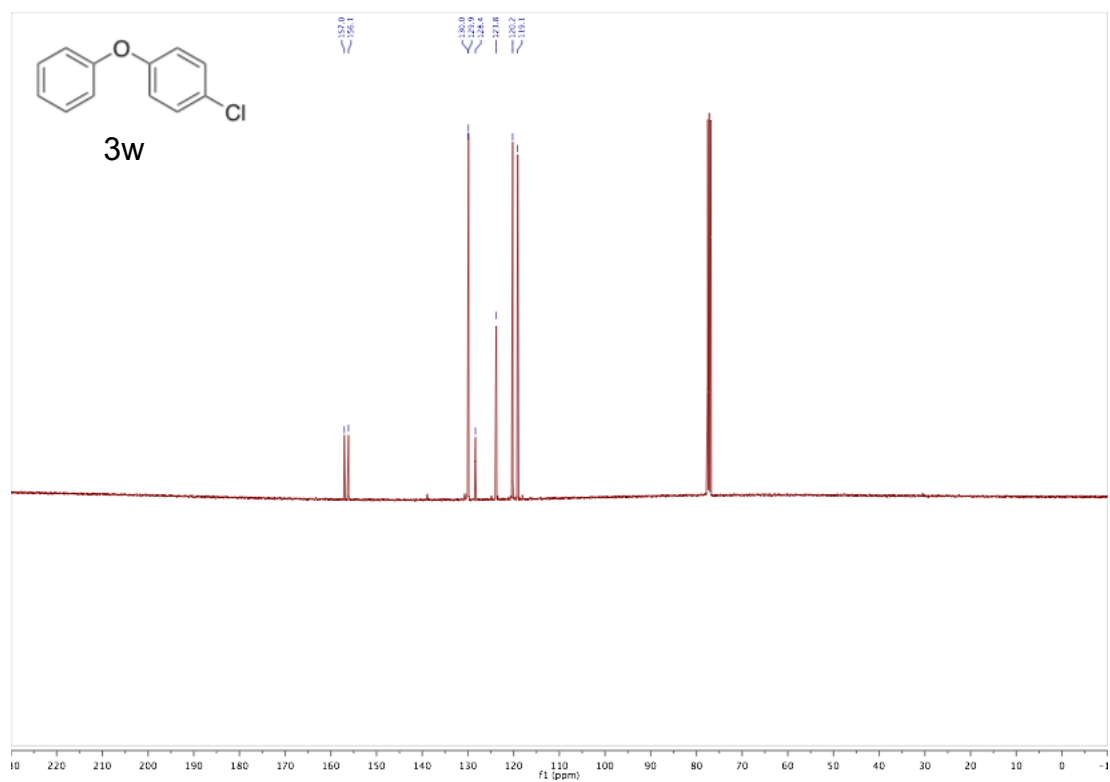



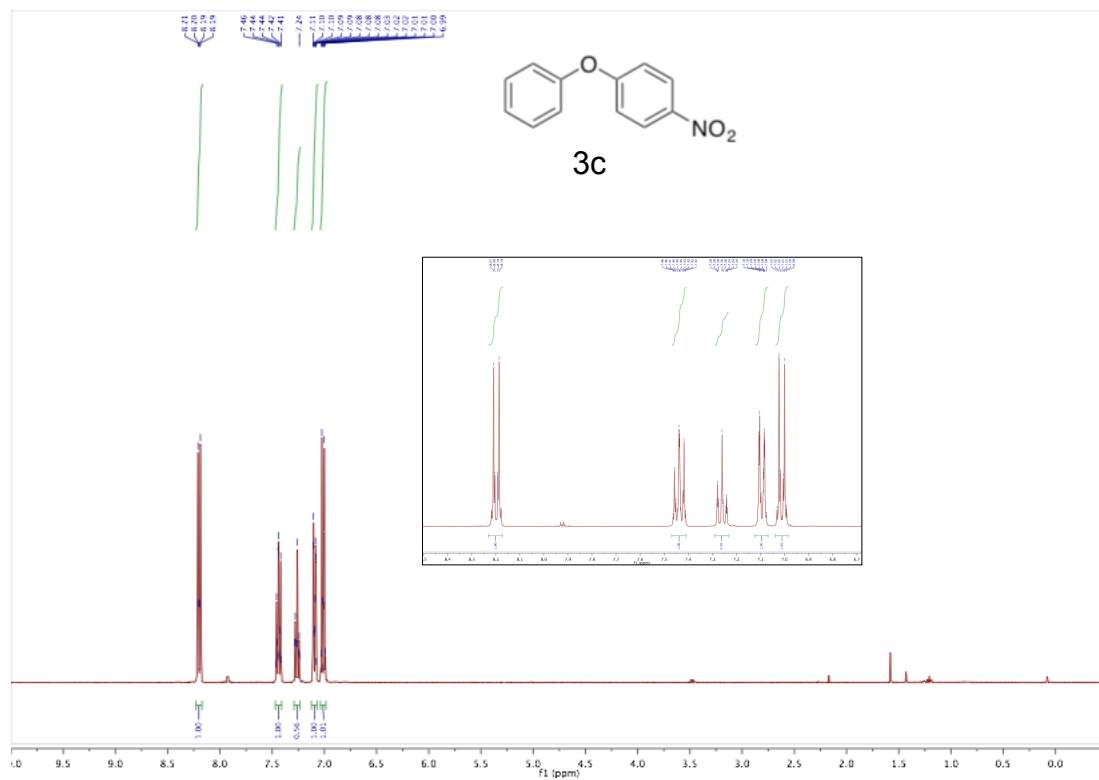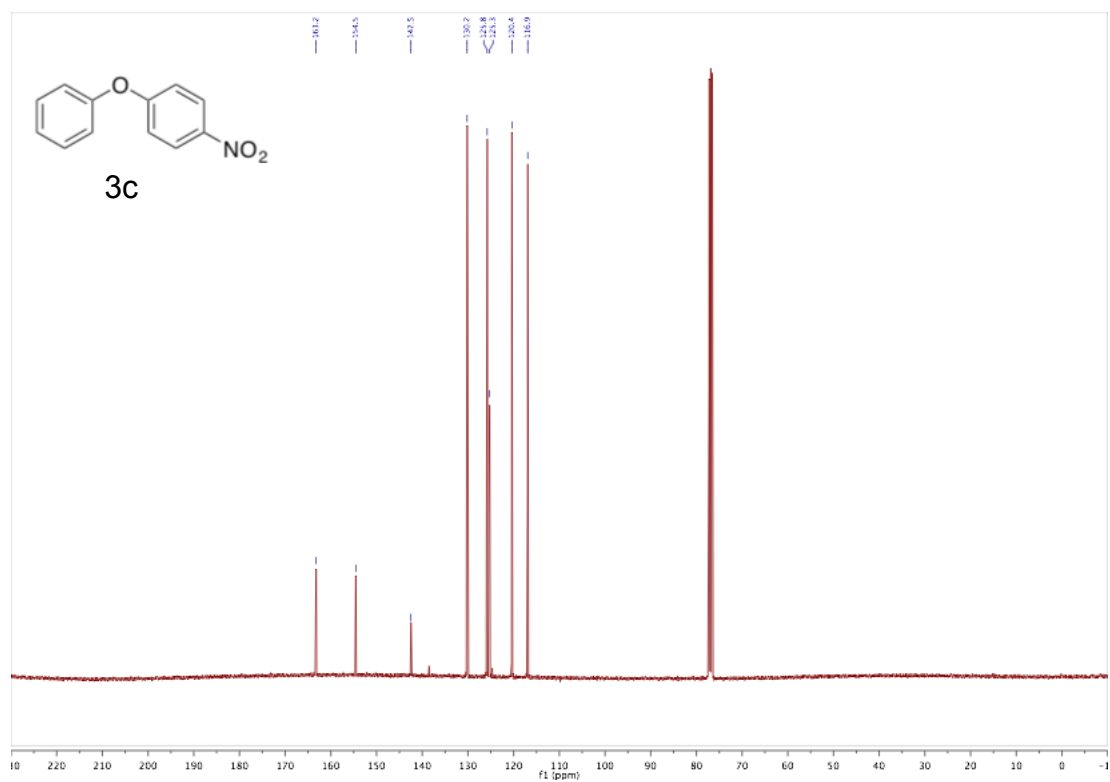



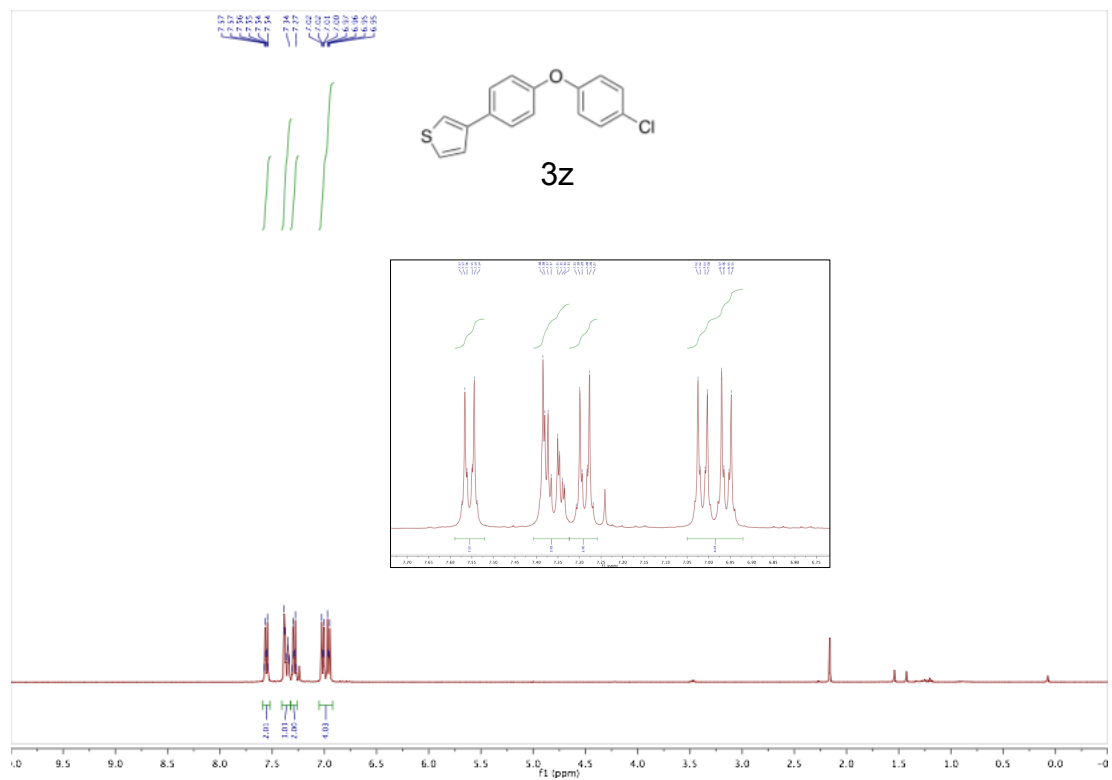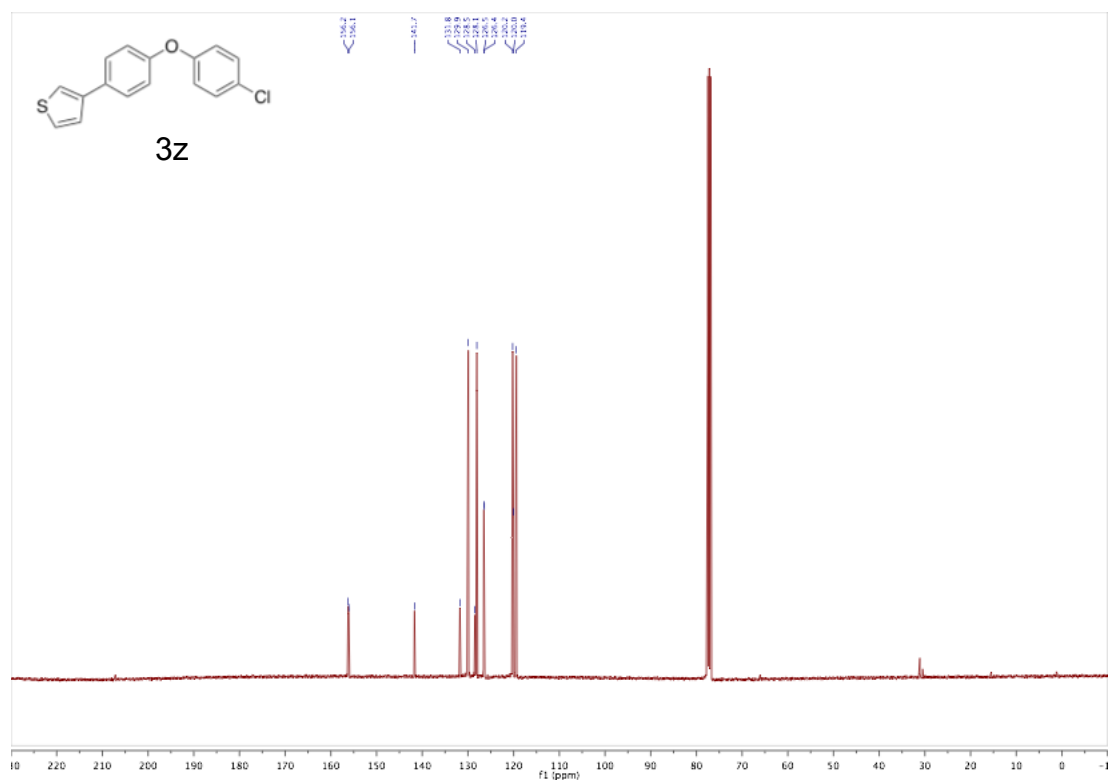

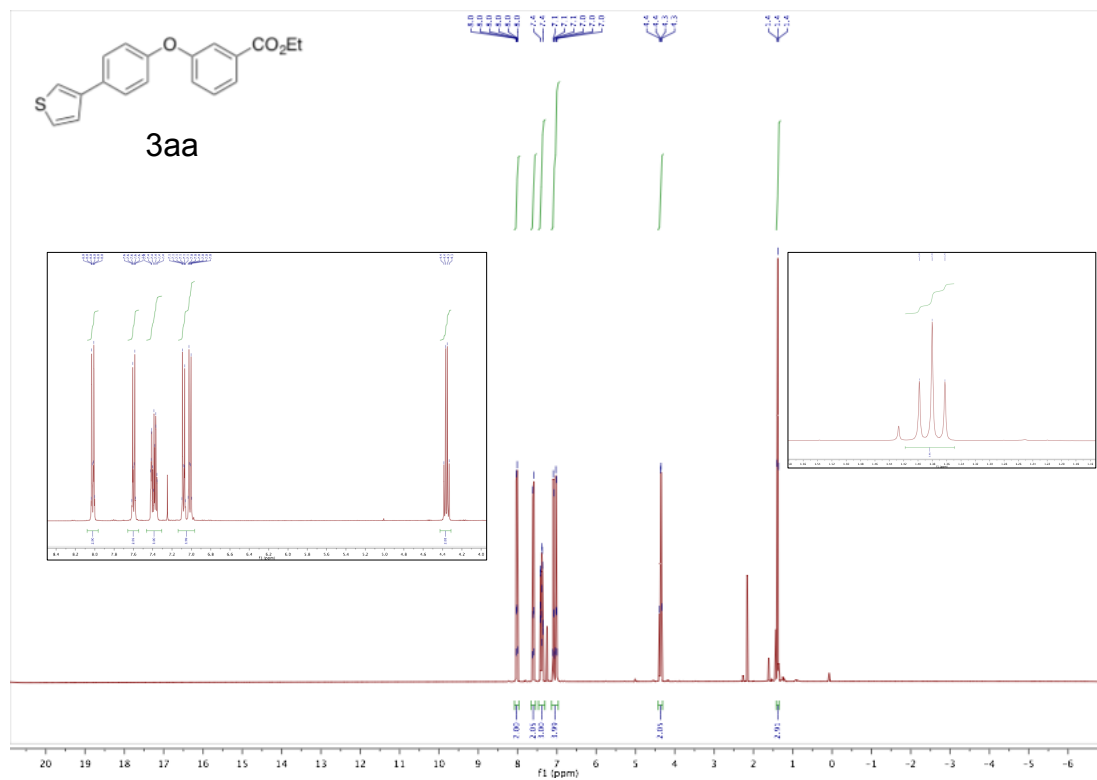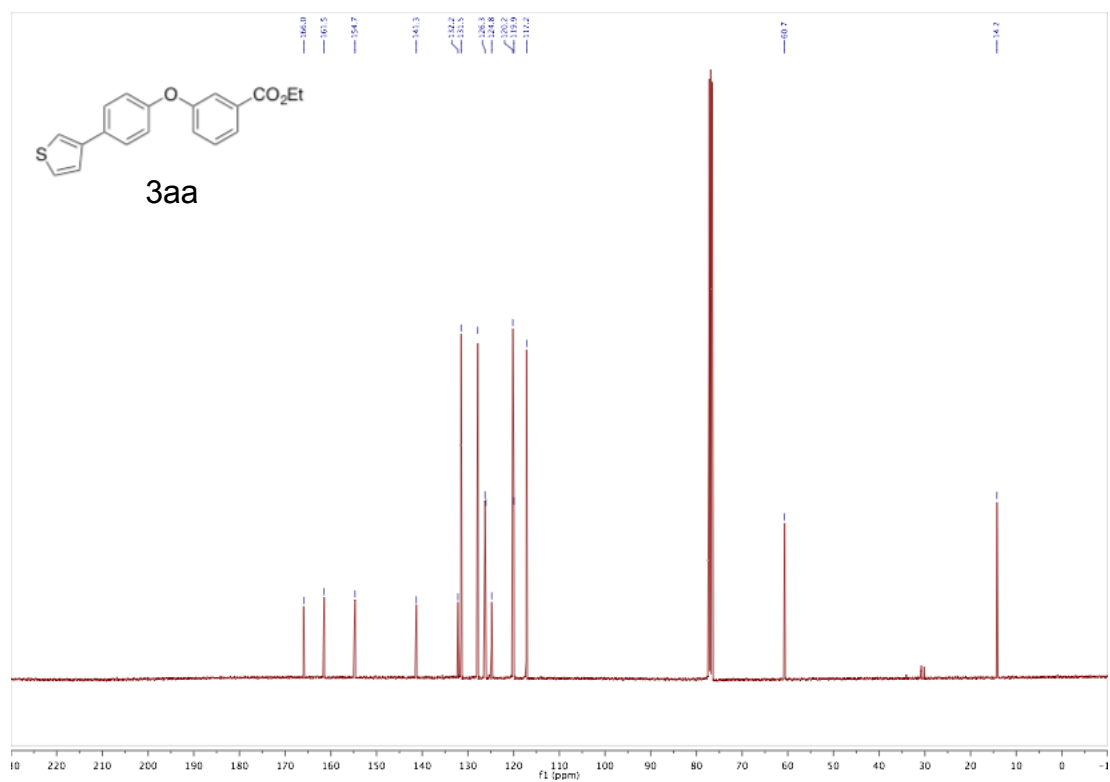

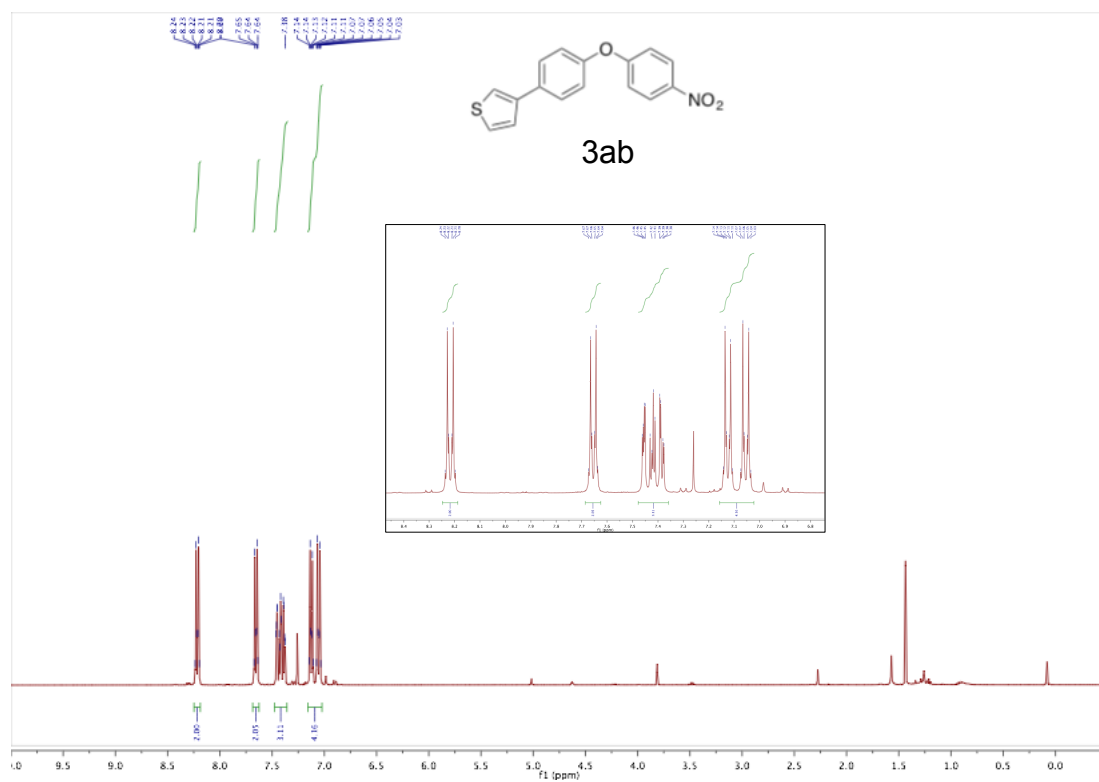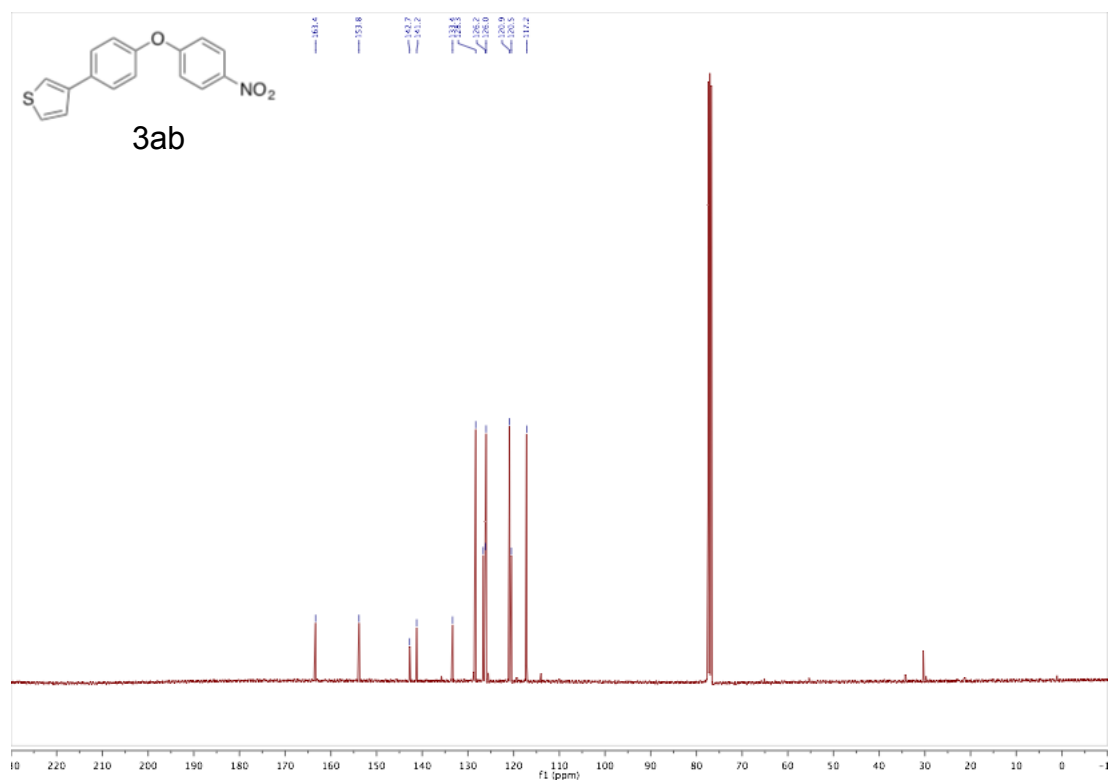

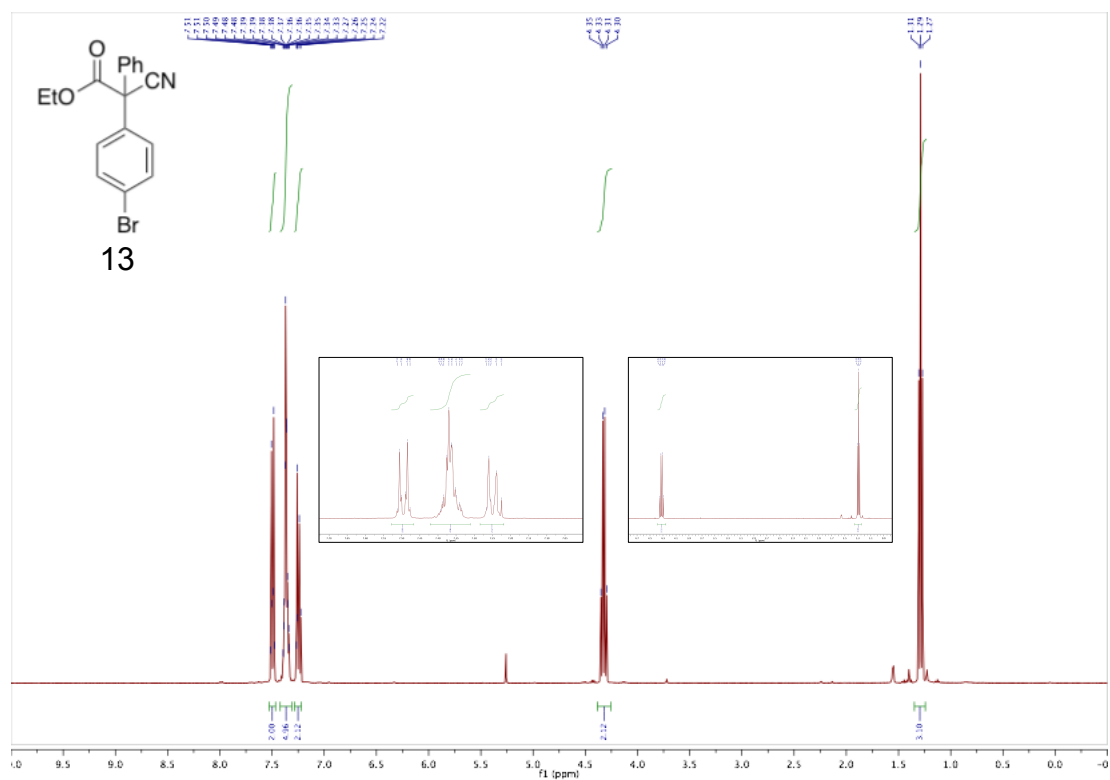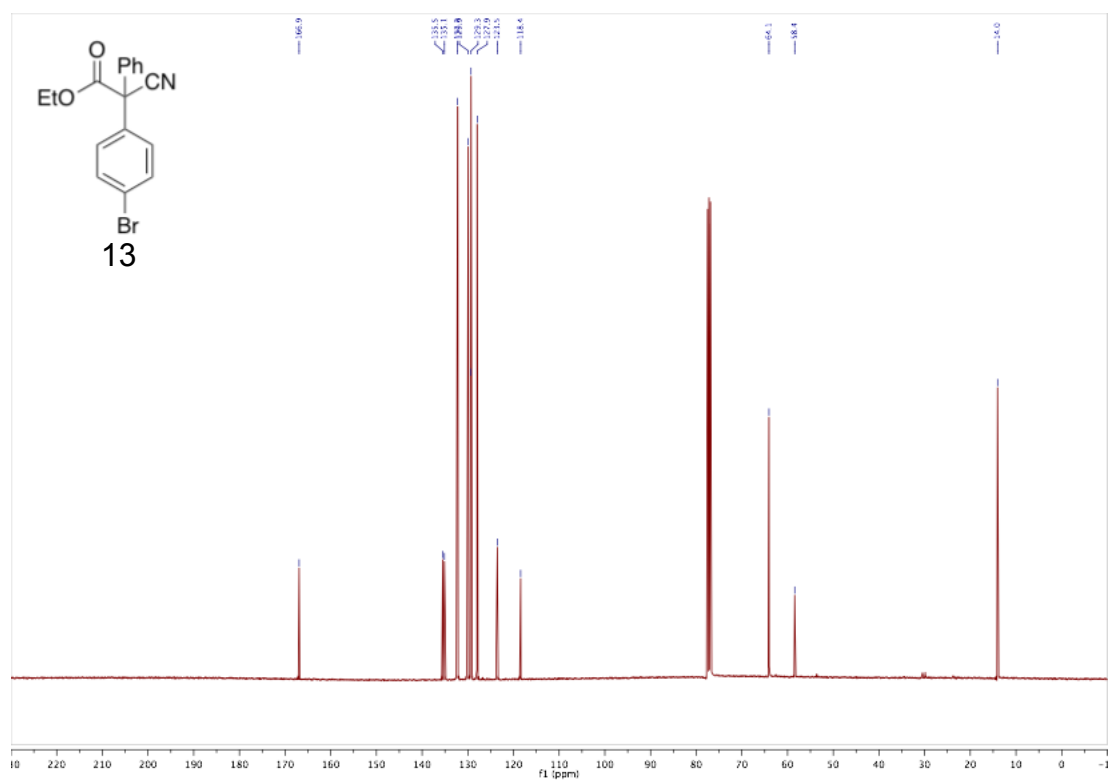

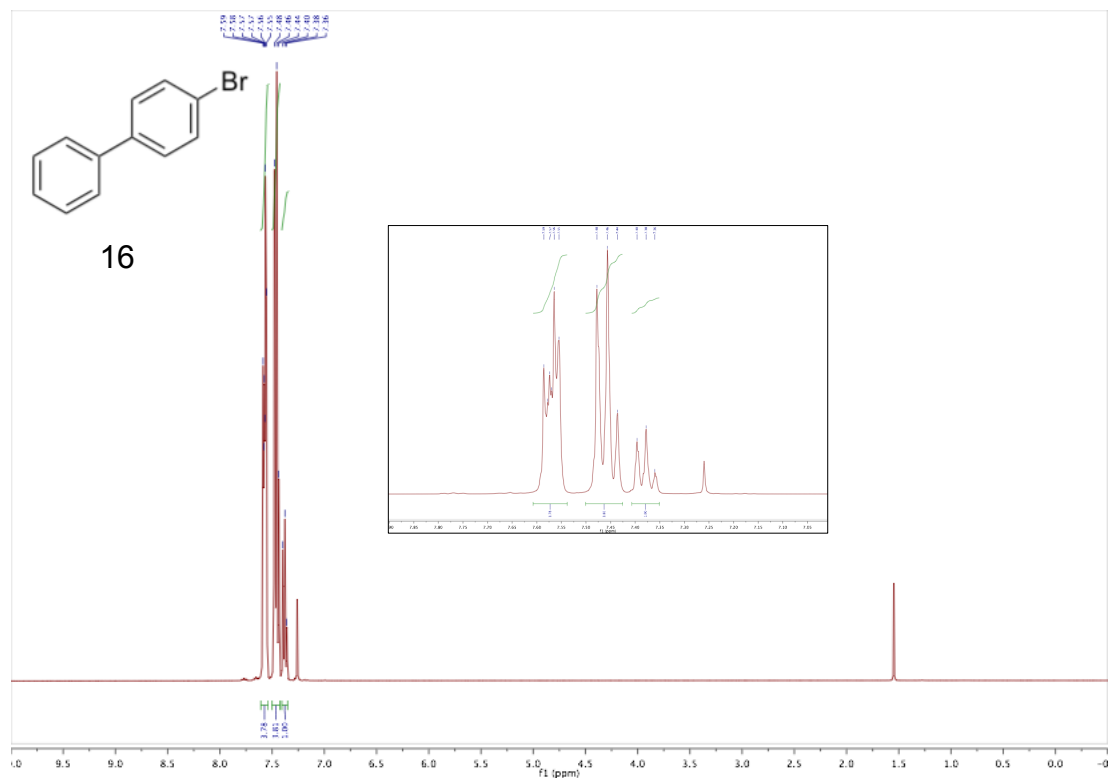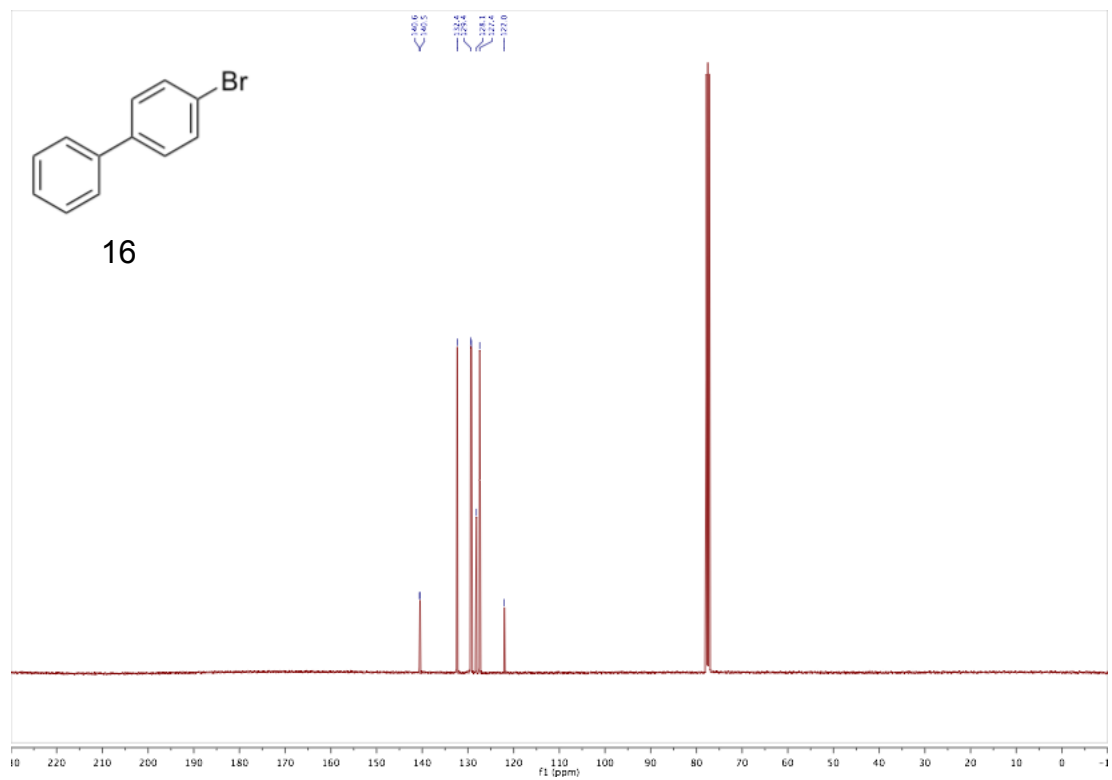

Supplement: Supplementary file 1 [file SC-006-C4SC02856B-s001.pdf]
