# Supplementary material for: A counteranion triggered arylation strategy using diaryliodonium fluorides
Source: Chem Sci. 2014 Nov 12;6(2):1277–81. doi: 10.1039/c4sc02856b (PMC5811167; doi:10.1039/c4sc02856b)
Supplement: Supplementary file 2 [file SC-006-C4SC02856B-s002.pdf]

Electronic Supplementary Material (ESI) for Chemical Science.
This journal is © The Royal Society of Chemistry 2014

1. General Information

Proton nuclear magnetic resonance (^1H NMR) spectra were recorded at ambient temperature on a Bruker AM 400 (400 MHz) or an Avance 500 (500 MHz) spectrometer. Chemical shifts (∂) are reported in pm and quoted to the nearest 0.01 ppm relative to the residual protons in $CDCl_3$ (7.26 ppm) and coupling constants (*J*) are quoted in Hertz. Data are reported as follows: Chemical shift (multiplicity, coupling constants, number of protons). Coupling constants were quoted to the nearest 0.1 Hz and multiplicity reported according to the following convention: s = singlet, d = doublet, t = triplet, q = quartet, qn = quintet, spt = septet, m = multiplet, br = broad. Where coincident coupling constants have been observed, the apparent (app) multiplicity of the proton resonance has been reported.

Carbon nuclear magnetic resonance (^{13}C NMR) spectra were recorded at ambient temperature on a Bruker AM 400 (101 MHz) or an Avance 500 (126 MHz) spectrometer. Chemical shift (∂) was measured in ppm and quoted to the nearest 0.1 ppm relative to the residual solvent peaks in $CDCl_3$ (77.16 ppm).

Infrared (IR) spectra were recorded on a Perkin Elmer Spectrum One-FT-IR spectrometer as thin films deposited in dichloromethane. High-Resolution Mass-Spectrometry (HRMS) was conducted by the EPSRC Mass Spectrometry Service at the University of Swansea. Melting points (m.p.) were recorded using a Gallenkamp melting-point apparatus and are reported uncorrected.

Analytical thin layer chromatography (TLC) was performed using pre-coated Merck glass backed silica gel plates (Silica gel 60 F254). Flash column chromatography was undertaken on Fluka or Material Harvest silica gel (230-400 mesh) under a positive pressure of nitrogen unless otherwise stated. Visualization was achieved under ultraviolet light (254 nm) and chemical staining with ceric ammonium molybdate or basic permanganate solutions as appropriate.

Tetrahydrofuran (THF), toluene, hexane, diethyl ether, dimethylformamide, dimethylsulfoxide, ethyl acetate, 1,4-dioxane, dichloroethane, acetonitrile, methanol and dichloromethane were dried and distilled using standard methods. All reagents were purchased at the highest commercial quality and used without further purification. All reactions were monitored by TLC. Reactions were not carried out under anhydrous conditions unless otherwise stated.

2. Reaction Optimisation

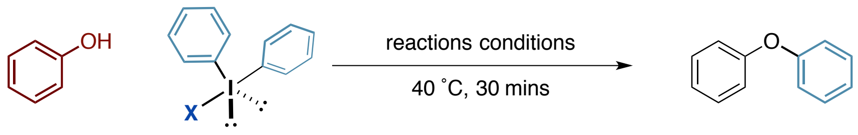

1a **2** **3a**

Table 1: Reaction Optimisation: Base Screen

Entry	X	Base	Solvent	Yield (%)*
1	OTf	$NaHCO_3$	dichloromethane	0
2	F	$NaHCO_3$	dichloromethane	95
3	F	$KHCO_3$	dichloromethane	32
4	F	Na_2CO_3	dichloromethane	84
5	F	K_2CO_3	dichloromethane	47
6	F	Cs_2CO_3	dichloromethane	92
7	F	Ca_2CO_3	dichloromethane	60

Table 2: Reaction Optimisation: Solvent Screen

Entry	X	Base	Solvent	Yield (%)·
1	F	$NaHCO_3$	dichloromethane	95
2	F	$NaHCO_3$	toluene	42
3	F	$NaHCO_3$	acetonitrile	90
4	F	$NaHCO_3$	diethyl ether	44
5	F	$NaHCO_3$	tetrahydrofuran	50
6	F	$NaHCO_3$	1,4-dioxane	38
7	F	$NaHCO_3$	ethyl acetate	89
8	F	$NaHCO_3$	dimethylformamide	89
9	F	$NaHCO_3$	dimethylsulfoxide	10
10	F	$NaHCO_3$	methanol	15

· GC yield with triphenylmethane as the internal standard

3. Synthesis of Diphenyliodonium Fluoride (2b)

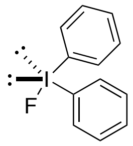

Prepared according to a procedure by Rawal.[1] Diphenyliodonium chloride (10 g, 31.6 mmol) was dissolved in H_2O (600 mL) in a round-bottomed flask and the mixture was warmed until a homogeneous solution was obtained. Potassium iodide (10.5 g, 63.2 mmol) was added at this temperature and precipitation was immediately observed. The resulting suspension was cooled to 23 °C and filtered. The collected solids were dried under vacuum overnight. The diphenyliodonium iodide salt was used directly in the next step. To a vigorously stirred solution of silver fluoride (2.3 g, 18.1 mmol) in H_2O (80 mL) under nitrogen was added diphenyliodonium iodide (7.8 g, 19 mmol) at 0 °C. The flask was removed from the ice bath after 10 minutes and wrapped with aluminium foil. The reaction mixture was further stirred in the dark at 23 °C overnight. The resulting suspension was filtered and the solid washed with H_2O (2 x 20 mL). The collected filtrate was concentrated, azeotroped with toluene a few times before the solids were dried under vacuum overnight to provided the title compound (5.48 g, 96%) as an off-white solid.

^{1}H NMR (400 MHz, $CDCl_3$): δ 7.88-7.86 (m, 4H), 7.50-7.45 (m, 2H), 7.38-7.33 (m, 4H); **^{13}C NMR** (101 MHz, $CDCl_3$): δ 134.3, 131.3, 130.9, 119.9; **^{19}F NMR** (376 MHz, $CDCl_3$): δ -43.4.

These data are in accordance with the literature.[1]

[1] T. Iwama, V. B. Birman, S. A. Kozmin and A. Rawal, *Org. Lett.* **1999**, *1*, 673.

4. Phenylation of Functionalized Phenols

4.1 General Procedure A for the Phenylation of Functionalized Phenols/Heterocycles With Diphenyliodonium Fluoride

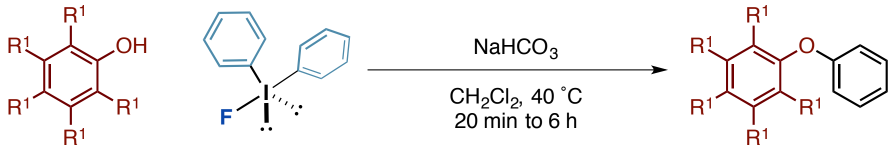

To a round-bottomed flask was charged the phenol/heterocycle (1.0 mmol, 1 equiv), sodium hydrogen carbonate (2.4 mmol, 2.4 equiv) and diphenyliodonium fluoride **2b** (1.2 mmol, 1.2 equiv). This was taken up in dichloromethane (20 mL) and the reaction stirred at 40 °C for the specified length of time. The reaction mixture was diluted with water (20 mL) and the organic layer extracted with dichloromethane (2 x 10 mL), dried over $MgSO_4$ and concentrated *in vacuo*. The crude residue was purified by flash chromatography to yield the corresponding diaryl ether.

Diphenylether (3a)

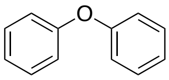

Prepared according to General Procedure A from phenol (94 mg, 1.0 mmol), sodium hydrogen carbonate (200 mg, 2.4 mmol) and diphenyliodonium fluoride **2b** (360 mg, 1.2 mmol) in dichloromethane (20 mL) at 40 °C for 1 hour. Purification by flash chromatography (petroleum ether 40-60°) gave compound **3a** (141 mg, 83%) as a colourless oil.

R_f 0.47 (petroleum ether 40-60°); **IR** υ max (film, cm^{-1}): 3040, 2342, 1583, 1486, 1233; **^{1}H NMR** (400 MHz, $CDCl_3$): δ 7.37-7.33 (m, 4H), 7.11 (tt, *J* = 7.1, 1.1 Hz, 2H), 7.05-7.02 (m, 4H); **^{13}C NMR** (101 MHz, $CDCl_3$): δ 157.7, 130.2, 123.7, 119.3; **HRMS** (ESI) calculated for $C_{12}H_{11}O$ $[M + H]^+$ m/z requires 171.0810, found 171.0803.

These data are in accordance with those of the commercial chemical (CAS Number: 101-84-8).

Methyl 4-phenoxybenzoate (3b)

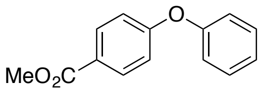

Prepared according to General Procedure A from methyl 4-hydroxybenzoate (152 mg, 1.0 mmol), sodium hydrogen carbonate (200 mg, 2.4 mmol) and diphenyliodonium fluoride **2b** (360 mg, 1.2 mmol) in dichloromethane (20 mL) at 40 °C for 6 hours. Purification by flash chromatography (10% diethyl ether in petroleum ether 40-60°) gave compound **3b** (191 mg, 83%) as a white solid.

R$_f$ 0.19 (5% diethyl ether in petroleum ether 40-60°); **m.p.** 59-60 °C; **IR** υ max (film, cm^{-1}): 1716, 1588, 1490, 1283, 1242; **^{1}H NMR** (400 MHz, $CDCl_3$): δ 8.02-7.99 (m, 2H), 7.41-7.37 (m, 2H), 7.21-7.17 (m, 1H), 7.08-7.05 (m, 2H), 7.00-6.97 (m, 2H), 3.90 (s, 3H); **^{13}C NMR** (101 MHz, $CDCl_3$): δ 166.7, 161.9, 155.6, 131.7, 130.0, 124.5 (2 Cs), 120.1, 117.3, 52.1; **HRMS** (ESI) calculated for $C_{14}H_{13}O_3$ $[M + H]^+$ m/z requires 229.0865, found 229.0857.

These data are in accordance with the literature.[2]

1-nitro-4-phenoxybenzene (3c)

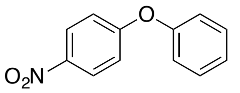

Prepared according to General Procedure A from 4-nitrophenol (134 mg, 1.0 mmol), sodium hydrogen carbonate (200 mg, 2.4 mmol) and diphenyliodonium fluoride **2b** (360 mg, 1.2 mmol) in dichloromethane (20 mL) at 40 °C for 5 hours. Purification by flash chromatography (10% diethyl ether in petroleum ether 40-60°) gave compound **3c** (186 mg, 86%) as a yellow solid.

R$_f$ 0.74 (5% diethyl ether in petroleum ether 40-60°); **m.p.** 56-57 °C; **IR** υ max (film, cm^{-1}): 3020, 2343, 1583, 1515, 1487, 1343, 1247; **^{1}H NMR** (400 MHz, $CDCl_3$): δ 8.22-8.19 (m, 2H), 7.46-7.42 (m, 2H), 7.28-7.24 (m, 1H), 7.10-7.08 (m, 2H), 7.03-7.00 (m, 2H); **^{13}C NMR** (101 MHz, $CDCl_3$): δ 163.6, 154.9, 142.8, 130.5, 126.1, 125.6, 120.7, 117.3; **HRMS** (ESI) calculated for $C_{12}H_{10}NO_3$ $[M + H]^+$ m/z requires 216.0661, found 216.0655.

---

[2] T. Hu, T. Schultz, C. Torborg, X. Chen, J. Wang, M. Beller and J. Huang, *Chem. Commun.* **2009**, *47*, 7330.

These data are in accordance with the literature.[3]

4-phenoxy-1,1'-biphenyl (3d)

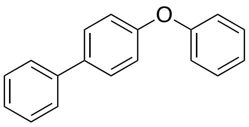

Prepared according to General Procedure A from 4-phenylphenol (170 mg, 1.0 mmol), sodium hydrogen carbonate (200 mg, 2.4 mmol) and diphenyliodonium fluoride **2b** (360 mg, 1.2 mmol) in dichloromethane (20 mL) at 40 °C for 5 hours. Purification by flash chromatography (10% diethyl ether in petroleum ether 40-60°) gave compound **3d** (210 mg, 85%) as a white solid.

R_f 0.19 (petroleum ether 40-60°); **m.p.** 68-69 °C; **IR** υ max (film, cm^{-1}): 2975, 1590, 1484, 1237; **^{1}H NMR** (400 MHz, $CDCl_3$): δ 7.58-7.54 (m, 4H), 7.45-7.42 (m, 2H), 7.38-7.31 (m, 3H), 7.14-7.11 (m, 1H), 7.09-7.05 (m, 4H); **^{13}C NMR** (101 MHz, $CDCl_3$): δ 157.1, 156.8, 140.5, 136.2, 129.7, 128.7, 128.4, 127.0, 126.8, 123.3, 119.0, 110.0; **HRMS** (ESI) calculated for $C_{18}H_{15}O$ [M + H]$^+$ m/z requires 247.1123, found 247.1118.

These data are in accordance with the literature.[3]

(4-phenoxyphenyl)methanol (3e)

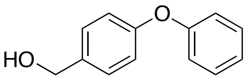

Prepared according to General Procedure A from 4-(hydroxymethyl)phenol (124 mg, 1.0 mmol), sodium hydrogen carbonate (200 mg, 2.4 mmol) and diphenyliodonium fluoride **2b** (360 mg, 1.2 mmol) in dichloromethane (20 mL) at 40 °C for 2 hours. Purification by flash chromatography (25% ethyl acetate in petroleum ether 40-60°) gave compound **3e** (162 mg, 82%) as a white solid.

R_f 0.47 (25% ethyl acetate in petroleum ether 40-60°); **m.p.** 50-51 °C; **IR** υ max (film, cm^{-1}): 3342, 1589, 1506, 1488, 1233; **^{1}H NMR** (400 MHz, $CDCl_3$): δ 7.36-7.32 (m, 4H), 7.12-7.09 (m, 1H), 7.02-6.99 (m, 4H), 4.67 (s, 2H), 1.70 (s, 1H); **^{13}C NMR** (101 MHz, $CDCl_3$): δ 157.1, 156.8, 135.6, 129.7, 128.6, 123.2, 118.9, 118.8, 64.9; **HRMS** (ESI) calculated for $C_{13}H_{12}O_2$ [M]$^+$ m/z requires 200.0837, found 200.0830.

[3] O. Bistri, A. Correa and C. Bolm, *Angew. Chem. Int. Ed.* **2008**, *47,* 586.

These data are in accordance with the literature.[4]

1,2,4-trimethyl-5-phenoxybenzene (3f)

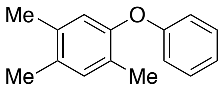

Prepared according to General Procedure A from 2,4,5-trimethylphenol (136 mg, 1.0 mmol), sodium hydrogen carbonate (200 mg, 2.4 mmol) and diphenyliodonium fluoride **2b** (360 mg, 1.2 mmol) in dichloromethane (20 mL) at 40 °C for 20 minutes. Purification by flash chromatography (petroleum ether 40-60°) gave compound **3f** (191 mg, 90%) as a colourless oil.

R_f 0.40 (petroleum ether 40-60°); **IR** υ max (film, cm^{-1}): 3073, 1590, 1489, 1218; **^{1}H NMR** (400 MHz, $CDCl_3$): δ 7.31-7.27 (m, 2H), 7.04-7.00 (m, 2H), 6.89 (dt, *J* = 7.7, 1.0 Hz, 2H), 6.75 (s, 1H), 2.24 (s, 3H), 2.20 (s, 3H), 2.17 (s, 3H); **^{13}C NMR** (101 MHz, $CDCl_3$): δ 158.4, 151.8, 135.3, 132.4, 132.3, 129.5, 127.1, 121.8, 121.5, 116.6, 19.4, 19.0, 15.6; **HRMS** (ESI) calculated for $C_{15}H_{17}O$ $[M + H]^+$ m/z requires 213.1279, found 213.1273.

1,3,5-trichloro-2-phenoxybenzene (3g)

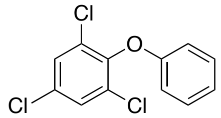

Prepared according to General Procedure A from 2,4,6-trichlorophenol (197 mg, 1.0 mmol), sodium hydrogen carbonate (200 mg, 2.4 mmol) and diphenyliodonium fluoride **2b** (360 mg, 1.2 mmol) in dichloromethane (20 mL) at 40 °C for 6 hours. Purification by flash chromatography (petroleum ether 40-60°) gave compound **3g** (231 mg, 85%) as a crystalline white solid.

R_f 0.63 (petroleum ether 40-60°); **m.p.** 57-58 °C; **IR** υ max (film, cm^{-1}): 3077, 1592, 1557, 1490, 1440, 1254, 1195; **^{1}H NMR** (400 MHz, $CDCl_3$): δ 7.42 (s, 2H), 7.32-7.29 (m, 2H), 7.09-7.06 (m, 1H), 6.84-6.82 (m, 2H); **^{13}C NMR** (101 MHz, $CDCl_3$): δ 156.7, 146.4, 131.2, 130.9, 129.9, 129.3, 123.0, 115.1; **HRMS** (ESI) calculated for $C_{12}H_8{}^{35}Cl_3O$ $[M + H]^+$ m/z requires 274.9611, found 274.9604.

1-phenoxy-2-(trifluoromethyl)benzene (3h)

[4] Q. Zhang, D. Wang, X. Wang and K. Ding, *J. Org. Chem.* **2009**, *74*, 7187.

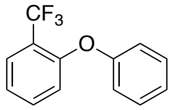

Prepared according to General Procedure A from 2-trifluoromethylphenol (162 mg, 1.0 mmol), sodium hydrogen carbonate (200 mg, 2.4 mmol) and diphenyliodonium fluoride **2b** (360 mg, 1.2 mmol) in dichloromethane (20 mL) at 40 °C for 1.5 hours. Purification by flash chromatography (petroleum ether 40-60°) gave compound **3h** (217 mg, 93%) as a colourless oil.

R$_f$ 0.52 (petroleum ether 40-60°); **IR** υ max (film, cm^{-1}): 3072, 1610, 1585, 1487, 1459, 1320, 1242; **^{1}H NMR** (400 MHz, CDCl$_3$): δ 7.67 (m, 1 H), 7.45-7.42 (m, 1H), 7.38-7.34 (m, 2H), 7.17-7.14 (m, 2H), 7.05-7.02 (m, 2H), 6.92 (d, *J* = 8.3 Hz, 1H); **^{13}C NMR** (101 MHz, CDCl$_3$): δ 156.7, 155.7, 133.4, 130.1, 127.4 (q, $^3J_{F\text{-}C}$ = 5.1 Hz), 124.3, 123.6 (q, $^1J_{F\text{-}C}$ = 272.5 Hz), 122.9, 121.5 (q, $^2J_{F\text{-}C}$ = 31.4 Hz), 119.7, 119.3; **^{19}F NMR** (376 MHz, CDCl$_3$): δ -61.9; **HRMS** (ESI) calculated for $C_{13}H_{10}F_3O$ [M + H]$^+$ m/z requires 239.0684, found 239.0679.

These data are in accordance with the literature.[5]

1,2,4,5-tetrafluoro-3-phenoxybenzene (3i)

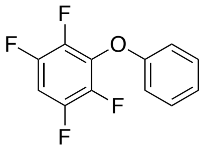

Prepared according to General Procedure A from 2,3,5,6-tetrafluorophenol (166 mg, 1.0 mmol), sodium hydrogen carbonate (200 mg, 2.4 mmol) and diphenyliodonium fluoride **2b** (360 mg, 1.2 mmol) in dichloromethane (20 mL) at 40 °C for 6 hours. Purification by flash chromatography (petroleum ether 40-60°) gave compound **3i** (175 mg, 72%) as a colourless oil.

R$_f$ 0.58 (petroleum ether 40-60°); **IR** υ max (film, cm^{-1}): 3081, 1641, 1591, 1519, 1485, 1201; **^{1}H NMR** (400 MHz, CDCl$_3$): δ; 7.36-7.32 (m, 2H), 7.11 (tt, *J* = 7.4, 1.0 Hz, 1H), 7.01 – 6.94 (m, 3H); **^{13}C NMR** (101 MHz, CDCl$_3$): δ 157.1, 146.6 (dtd, $J_{F\text{-}C}$ = 248.4, 12.3, 4.2), 141.6 (dddd, $J_{F\text{-}C}$ = 250.1, 14.8, 4.8, 2.4 Hz), 134.2 (ddt, $J_{F\text{-}C}$ = 17.2, 8.9, 4.1 Hz), 129.8, 123.7, 115.5, 101.8 (t, $J_{F\text{-}C}$ = 22.9 Hz); **^{19}F NMR** (376 MHz, CDCl$_3$): δ 154.3-154.0 (m, 2H), 138.4-

[5] T. D. Senecal, A. T. Parsons and S. L. Buchwald, *J. Org. Chem.* **2011**, *76*, 1174.

138.5 (m, 2H); **HRMS** (ESI) calculated for $C_{12}H_7F_4O$ $[M + H]^+$ m/z requires 243.0433, found 243.0429.

These data are in accordance with the literature.[6]

(4-phenoxyphenyl)(phenyl)methanone (3j)

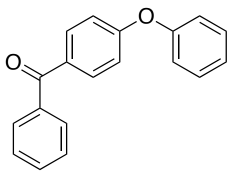

Prepared according to General Procedure A from 4-hydroxybenzophenone (198 mg, 1.0 mmol), sodium hydrogen carbonate (200 mg, 2.4 mmol) and diphenyliodonium fluoride **2b** (360 mg, 1.2 mmol) in dichloromethane (20 mL) at 40 °C for 5 hours. Purification by flash chromatography (20% diethyl ether in petroleum ether 40-60°) gave compound **3j** (225 mg, 82%) as a white solid.

R_f 0.60 (20% diethyl ether in petroleum ether 40-60°); **m.p.** 69-70 °C (lit. 74-75 °C); **IR** υ max (film, cm^{-1}): 3101, 1654, 1586, 1489, 1243; **^{1}H NMR** (400 MHz, $CDCl_3$): δ 7.83-7.81 (m, 2H), 7.79-7.77 (m, 2H), 7.48-7.47 (m, 2H), 7.39-7.42 (m, 2H), 7.22-7.19 (m, 2H), 7.11-7.09 (m, 2H), 7.05-7.02 (m, 2H); **^{13}C NMR** (101 MHz, $CDCl_3$): δ 195.5, 161.6, 155.5, 137.9, 132.4, 132.1, 131.9, 130.0, 129.8, 128.2, 124.5, 120.1, 117.1; **HRMS** (ESI) calculated for $C_{19}H_{15}O$ $[M + H]^+$ m/z requires 275.1072, found 275.1070.

These data are in accordance with the literature.[7]

3,5-dimethyl-4-phenoxybenzaldehyde (3k)

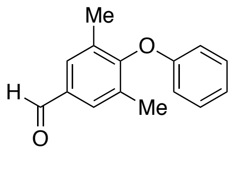

Prepared according to General Procedure A from 4-hydroxy-3,5-dimethylbenzaldehyde (150 mg, 1.0 mmol), sodium hydrogen carbonate (200 mg, 2.4 mmol) and diphenyliodonium fluoride **2b** (360 mg, 1.2 mmol) in dichloromethane (20 mL) at 40 °C for 4 hours.

[6] L. Sun, M. Rong, D. Kong, Z. Bai, Y. Yuan and Z. Weng, *J. Fluorine. Chem.* **2013**, *150*, 117.
[7] J. Chen, Z. Wang, X. Zheng, J. Ding, M. Liu and H. W. *Tetrahedron*, **2012**, *68*, 8905.

Purification by flash chromatography (5% diethyl ether in petroleum ether 40-60°) gave compound **3k** (186 mg, 82%) as a white solid.

R$_f$ 0.18 (5% diethyl ether in petroleum ether 40-60°); **m.p.** 60-61 °C; IR υ max (film, cm^{-1}): 3333, 1692, 1589, 1491, 1305, 1223; **^{1}H NMR** (400 MHz, $CDCl_3$): δ 9.96 (s, 1H), 7.66 (s, 2H), 7.30-7.26 (m, 2H), 7.03-7.03 (m, 1H), 6.76 (dt, *J* = 7.8, 1.0 Hz, 2H), 2.21 (s, 6H); **^{13}C NMR** (101 MHz, $CDCl_3$): δ 191.3, 156.8, 156.1, 133.0, 132.5, 130.4, 129.5, 121.7, 114.3, 16.2; **HRMS** (ESI) calculated for $C_{15}H_{15}O_2$ [M + H]$^+$ m/z requires 227.1072, found 227.1065.

3-phenoxybenzaldehyde (3l)

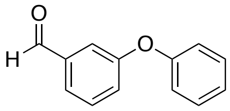

Prepared according to General Procedure A from methyl 2-hydroxybenzaldehyde (122 mg, 1.0 mmol), sodium hydrogen carbonate (200 mg, 2.4 mmol) and diphenyliodonium fluoride **2b** (360 mg, 1.2 mmol) in dichloromethane (20 mL) at 40 °C for 4 hours. Purification by flash chromatography (5% diethyl ether in petroleum ether 40-60°) gave compound **3l** (153 mg, 77%) as a colourless liquid.

R$_f$ 0.17 (5% diethyl ether in petroleum ether 40-60°); **IR** υ max (film, cm^{-1}): 3066, 2821, 2731, 1697, 1582, 1480, 1448, 1246, 1210; **^{1}H NMR** (400 MHz, $CDCl_3$): δ 9.96 (s, 1H), 7.63-7.61 (m, 1H), 7.51 (t, *J* = 7.9 Hz, 1H), 7.48-7.47 (m, 1H), 7.41-7.37 (m, 2H), 7.31-7.29 (m, 1H), 7.20-7.17 (m, 1H), 7.07-7.04 (m, 2H); **^{13}C NMR** (101 MHz, $CDCl_3$): δ 191.5, 158.3, 156.1, 138.0, 130.4, 130.0, 124.6, 124.5, 124.1, 119.4, 118.0; **HRMS** (ESI) calculated for $C_{13}H_{11}O_2$ [M + H]$^+$ m/z requires 199.0759, found 199.0753.

These data are in accordance with those of the commercial chemical (CAS Number: 39515-51-0).

2,2'-diphenoxy-1,1'-binaphthalene (3m)

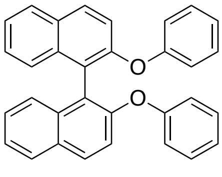

Prepared according to General Procedure A from 1,1'-bi-2-naphthol (286 mg, 1.0 mmol), sodium hydrogen carbonate (400 mg, 4.8 mmol) and diphenyliodonium fluoride **2b** (720 mg, 2.4 mmol) in dichloromethane (20 mL) at 40 °C for 4 hours. Purification by flash chromatography (10% diethyl ether in petroleum ether 40-60°) gave compound **3n** (432 mg, 99%) as an off-white solid.

R$_f$ 0.70 (10% diethyl ether in petroleum ether 40-60°); **m.p.** 214-216 °C (lit. 213-215 °C) ; **IR** υ max (film, cm^{-1}): 1588, 1489, 1238; **^{1}H NMR** (400 MHz, $CDCl_3$): δ 7.87-7.85 (m, 4H), 7.41-7.37 (m, 2H), 7.31-7.28 (m, 4H), 7.20 (d, *J* = 9.0 Hz, 2H), 7.13-7.10 (m, 4H), 6.96-6.92 (m, 2H), 6.82-6.79 (m, 4H); **^{13}C NMR** (101 MHz, $CDCl_3$): δ 157.5, 152.6, 134.9, 134.3, 132.2, 130.4, 129.7, 129.3, 128.1, 126.6, 125.8, 124.7, 122.7, 122.2, 119.3, 119.0; **HRMS** (ESI) calculated for $C_{32}H_{23}O_2$ $[M + H]^+$ m/z requires 439.1698, found 439.1690.

These data are in accordance with the literature.[8]

3-(4-phenoxyphenyl)thiophene (3n)

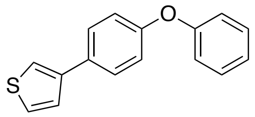

Prepared according to General Procedure A from 4-(thiophen-3-yl)phenol (176 mg, 1.0 mmol), sodium hydrogen carbonate (200 mg, 2.4 mmol) and diphenyliodonium fluoride **2b** (360 mg, 1.2 mmol) in dichloromethane (20 mL) at 40 °C for 2 hours. Purification by flash chromatography (2% diethyl ether in petroleum ether 40-60°) gave compound **3m** (253 mg, 100%) as a white solid.

R$_f$ 0.71 (2% diethyl ether in petroleum ether 40-60°); **m.p.** 116-117 °C; **IR** υ max (film, cm^{-1}): 3150, 1489, 1239; **^{1}H NMR** (400 MHz, $CDCl_3$): δ 7.58-7.55 (m, 2H), 7.40-7.33 (m, 5H), 7.14-7.10 (m, 1H), 7.06-7.03 (m, 4H); **^{13}C NMR** (101 MHz, $CDCl_3$): δ 157.4, 156.7, 141.9, 131.4, 130.0, 128.0, 126.4, 123.5, 119.9, 119.3, 119.1; **HRMS** (ESI) calculated for $C_{16}H_{13}OS$ $[M + H]^+$ m/z requires 253.0687, found 253.0685.

4-phenoxypyridine (3o)

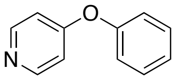

[8] N. Jalalian, E. E. Ishikawa, L. F. Silva Jr and B. Olofsson, *Org. Lett.* **2011**, *13*, 1552.

Prepared according to General Procedure A from 4-hydroxypyridine (95 mg, 1.0 mmol), sodium hydrogen carbonate (200 mg, 2.4 mmol) and diphenyliodonium fluoride **2b** (360 mg, 1.2 mmol) in dichloromethane (20 mL) at 40 °C for 16 hours. Purification by flash chromatography (70% ethyl acetate in petroleum ether 40-60°) gave compound **3o** (123 mg, 72%) as a yellow solid.

R$_f$ 0.49 (70% ethyl acetate in petroleum ether 40-60°); **m.p.** 44-45 °C (lit. 42-43 °C); **IR υ** max (film, cm^{-1}): 3038, 1578, 1485, 1417, 1259, 1209; **^{1}H NMR** (400 MHz, $CDCl_3$): δ 8.47-8.46 (m, 2H), 7.45-7.41 (m, 2H), 7.28-7.26 (m, 1H), 7.12-7.09 (m, 2H), 6.84-6.83 (m, 2H); **^{13}C NMR** (101 MHz, $CDCl_3$): δ 164.8, 154.0, 151.4, 130.2, 125.4, 120.1, 112.1; **HRMS** (ESI) calculated for $C_{11}H_{10}NO$ [M + H]$^+$ m/z requires 171.0762, found 172.0757.

These data are in accordance with the literature.[9]

3-phenoxypyridine (3p)

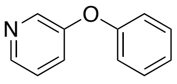

Prepared according to General Procedure A from 3-hydroxypyridine (95 mg, 1.0 mmol), sodium hydrogen carbonate (200 mg, 2.4 mmol) and diphenyliodonium fluoride **2b** (360 mg, 1.2 mmol) in dichloromethane (20 mL) at 40 °C for 20 hours. Purification by flash chromatography (20% ethyl acetate in petroleum ether 40-60°) gave compound **3p** (117 mg, 68%) as a yellow oil.

R$_f$ 0.57 (20% ethyl acetate in petroleum ether 40-60°); **IR υ** max (film, cm^{-1}): 3038, 1572, 1489, 1473, 1422, 1244; **^{1}H NMR** (400 MHz, $CDCl_3$): δ 8.41 (dd, *J* = 2.7, 0.7 Hz, 1H), 8.35 (dd, *J* = 4.5, 1.5 Hz, 1H), 7.38-7.34 (m, 2H), 7.28-7.23 (m, 2H), 7.15-7.13 (m, 1H), 7.04-7.01 (m, 2H); **^{13}C NMR** (101 MHz, $CDCl_3$): δ 156.2, 153.8, 144.2, 141.3, 129.9, 125.3, 124.0, 123.9, 118.9; **HRMS** (ESI) calculated for $C_{11}H_{10}NO$ [M + H]$^+$ m/z requires 171.0762, found 172.0754.

These data are in accordance with the literature.[8]

2-phenoxypyridine (3q)

---

[9] Y.-J. Cherng, *Tetrahedron,* **2002**, *58,* 4931.

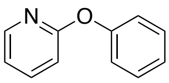

Prepared according to General Procedure A from 2-hydroxypyridine (95 mg, 1.0 mmol), sodium hydrogen carbonate (200 mg, 2.4 mmol) and diphenyliodonium fluoride **2b** (360 mg, 1.2 mmol) in dichloromethane (20 mL) at 40 °C for 16 hours. Purification by flash chromatography (30% ethyl acetate in petroleum ether 40-60°) gave compound **3q** (143 mg, 84%) as a yellow solid.

R$_f$ 0.32 (30% ethyl acetate in petroleum ether 40-60°); **m.p.** 123-124 °C; **IR** υ max (film, cm^{-1}): 2918, 1663, 1602, 1584, 1534, 1494, 1279; **^{1}H NMR** (400 MHz, $CDCl_3$): δ 7.51-7.47 (m, 2H), 7.44-7.37 (m, 4H), 7.33 (dd, *J* = 6.9, 2.1, 0.8 Hz, 1H), 6.66 (ddd, *J* = 9.3, 1.3, 0.8 Hz, 1H), 6.24 (td, *J* = 6.7, 1.4 Hz, 1H); **^{13}C NMR** (101 MHz, $CDCl_3$): δ 162.7, 141.2, 140.1, 138.2, 129.6, 128.7, 126.8, 122.2, 106.1; **HRMS** (ESI) calculated for $C_{11}H_{10}NO$ [M + H]$^+$ m/z requires 171.0762, found 172.0757.

2-phenoxyquinoline (3r)

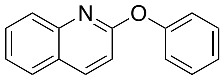

Prepared according to General Procedure A from 2-hydroxyquinoline (145 mg, 1.0 mmol), sodium hydrogen carbonate (200 mg, 2.4 mmol) and diphenyliodonium fluoride **2b** (360 mg, 1.2 mmol) in dichloromethane (20 mL) at room temperature for 16 hours. Purification by flash chromatography (20% ethyl acetate in petroleum ether 40-60°) gave compound **3r** (100 mg, 45%) as a yellow solid.

R$_f$ 0.33 (20% ethyl acetate in petroleum ether 40-60°); **m.p.** 44-45 °C; **IR** υ max (film, cm^{-1}): 2333, 2253, 903, 725; **^{1}H NMR** (400 MHz, $CDCl_3$): δ 8.99 (dd, *J* = 4.2, 1.7 Hz, 1H), 8.20 (dd, *J* = 8.4, 1.7 Hz, 1H), 7.55 (dd, *J* = 8.2, 1.2 Hz, 1H), 7.49-7.036 (m, 4H), 7.18-7.13 (m, 3H) 7.07 (dd, *J* = 7.7, 1.3 Hz, 1H); **^{13}C NMR** (101 MHz, $CDCl_3$): δ 157.2, 154.3, 150.3, 141.1, 136.2, 119.97, 129.93, 126.7, 124.1, 122.4, 122.1, 120.2, 115.5; **HRMS** (ESI) calculated for $C_{15}H_{12}NO$ [M + H]$^+$ m/z requires 222.0919, found 222.0913.

(8*R*,9*S*,13*S*)-13-methyl-3-phenoxy-6,7,8,9,11,12,13,14,15,16-decahydro-17*H*-cyclopenta[*a*]phenanthren-17-one (6)

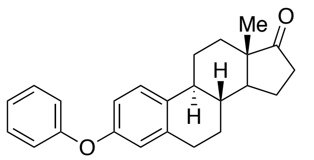

Prepared according to General Procedure A from estrone (270 mg, 1.0 mmol), sodium hydrogen carbonate (200 mg, 2.4 mmol) and diphenyliodonium fluoride **2b** (360 mg, 1.2 mmol) in dichloromethane (20 mL) at 40 °C for 2 hours. Purification by flash chromatography (25% diethyl ether in petroleum ether 40-60°) gave compound **6** (258 mg, 72%) as an off-white solid.

R$_f$ 0.38 (25% diethyl ether in petroleum ether 40-60°); **m.p.** 124-125 °C; **IR** υ max (film, cm^{-1}): 2934, 2876, 1734, 1589, 1488, 1238; **^{1}H NMR** (400 MHz, $CDCl_3$): δ 7.34-7.30 (m, 2H), 7.25 (d, *J* = 8.0 Hz, 1H), 7.10-7.07 (m, 1H), 7.01-6.99 (m, 2H), 6.80 (dd, *J* = 8.5, 2.7 Hz, 1H), 6.76 (d, *J* = 2.7 Hz, 1H), 2.89-2.86 (m, 2H), 2.54-2.48 (m, 1H), 2.43-2.39 (m, 1H), 2.31-2.26 (m, 1H), 2.19-2.11 (m, 1H), 2.09-1.95 (m, 3H), 1.66-1.58 (m, 2H), 1.56-1.41 (m, 4H), 0.93 (s, 3H); **^{13}C NMR** (101 MHz, $CDCl_3$): δ 221.0, 157.6, 155.2, 138.4, 134.9, 129.8, 126.8, 123.2, 119.2, 118.9, 116.6, 50.6, 48.2, 44.3, 38.4, 36.1, 31.8, 29.7, 26.7, 26.1, 21.8, 14.1; **HRMS** (ESI) calculated for $C_{24}H_{26}O_2$ $[M]^+$ m/z requires 347.2001, found 347.2003.

methyl (*S*)-2-((*tert*-butoxycarbonyl)amino)-3-(4-phenoxyphenyl)propanoate (8)

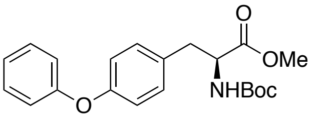

Prepared according to General Procedure A from commercially available methyl (*tert*-butoxycarbonyl)-*L*-tyrosinate **7** (295 mg, 1.0 mmol), sodium hydrogen carbonate (200 mg, 2.4 mmol) and diphenyliodonium fluoride **2b** (360 mg, 1.2 mmol) in dichloromethane (20 mL) at 40 °C for 30 min. Purification by flash chromatography (25% diethyl ether in petroleum ether 40-60°) gave compound **8** (341 mg, 92%) as a colourless oil.

R$_f$ 0.68 (25% diethyl ether in petroleum ether 40-60°); **IR** υ max (film, cm^{-1}): 3395, 2977, 1744, 1712, 1506, 1488, 1234, 1163; **^{1}H NMR** (400 MHz, $CDCl_3$): δ 7.35-7.31 (m, 2H), 7.11-7.08 (m, 3H), 7.00-6.92 (m, 4H), 5.03 (d, *J* = 7.0 Hz, 1H), 4.58 (d, *J* = 6.4 Hz, 1H), 3.72 (s, 3H), 3.13-2.99 (m, 2H), 1.42 (s, 9H); **^{13}C NMR** (101 MHz, $CDCl_3$) δ 172.4, 157.3, 156.5, 155.2, 130.9, 130.7, 129.9, 123.4, 119.0, 80.0, 54.6, 53.6, 52.4, 37.8, 28.4; **HRMS** (ESI) calculated for $C_{21}H_{26}NO_5$ $[M + H]^+$ requires 372.1811, found 372.1802. 100% *ee* by HPLC (Chiralcel AD column, 95:5 *i*-hexane/*i*-propanol).

These data are in accordance with the literature.[8]

methyl ((*S*)-2-((*S*)-2-((*tert*-butoxycarbonyl)amino)propanamido)-3-(4-phenoxyphenyl)propanoyl)-*L*-alaninate (10)

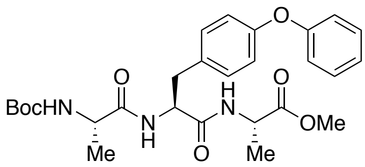

Prepared according to General Procedure A from tripeptide **9** (37 mg, 0.1 mmol), sodium hydrogen carbonate (20 mg, 0.24 mmol) and diphenyliodonium fluoride **2b** (36 mg, 0.12 mmol) in dichloromethane (2 mL) at 40 °C for 2 hours. Purification by flash chromatography (3% methanol in dichloromethane) gave compound **10** (43 mg, 84%) as a colourless oil.

R$_f$ 0.30 (3% methanol in dichloromethane); **IR** υ max (film, cm^{-1}): 3289, 2979, 1645, 1507, 1489, 1453, 1235, 1161, 736, 692; **^{1}H NMR** (400 MHz, $CDCl_3$): δ = 7.31 (t, *J*= 7.6 Hz, 2H,), 7.15 (d, *J*= 8.1 Hz, 2H), 7.10-7.06 (m, 1H), 6.97 (d, *J*= 8.1 Hz, 2H), 6.90 (d, *J*= 8.1 Hz, 2H), 6.84 - 6.71 (br s, 1H), 5.19 - 5.00 (br, 1H), 4.70-4.67 (m, 1H), 4.50-4.46 (m, 1H), 4.14 (br s, 1H, NH), 3.70 (s, 3H), 3.46 (s, 1H), 3.06-3.04 (m, 2H), 1.40 (s, 9H), 1.32 (dd, *J*= 16.7, 7.1 Hz, 6H); **^{13}C NMR** (101 MHz, $CDCl_3$) δ 173.3, 170.7, 157.6, 156.7, 156.0, 131.5, 131.1, 130.2, 123.7, 119.3, 80.8, 54.6, 52.9, 51.0, 48.6, 37.8, 30.2, 28.7, 18.7, 18.5; **HRMS** (ESI) calculated for $C_{27}H_{36}N_3O_7$ [M + H]$^+$ requires 514.2553, found 514.2553.

ethyl 2-cyano-2,2-diphenylacetate (12)

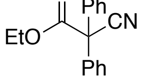

Prepared according to General Procedure A from ethyl 2-cyano-2-phenylacetate **11** (189 mg, 1.0 mmol), sodium hydrogen carbonate (200 mg, 2.4 mmol) and diphenyliodonium fluoride **2b** (360 mg, 1.2 mmol) in dichloromethane (20 mL) at 40 °C for 1.5 hours. Purification by flash chromatography (5% diethyl ether in petroleum ether 40-60°) gave compound **12** (198 mg, 75%) as a colourless oil.

R$_f$ 0.24 (5% diethyl ether in petroleum ether 40-60°); **IR** υ max (film, cm^{-1}): 2254, 1747, 1450, 1231, 1021, 904, 727, 696; **^{1}H NMR** (400 MHz, $CDCl_3$): δ = 7.42-7.38 (m, 5H,), 4.36

(q, *J*= 7.1 Hz, 2H), 1.33 (t, *J*= 7.1 Hz, 3H); **^{13}C NMR** (101 MHz, $CDCl_3$) δ 167.1, 135.8, 128.9, 127.9, 118.7, 63.7, 58.8, 13.9; **HRMS** (ESI) calculated for $C_{17}H_{19}N_2O_2$ $[M + NH_4]^+$ requires 283.1441, found 283.1438.

1,1'-biphenyl (15)

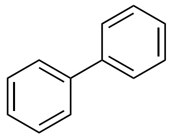

Prepared according to General Procedure A from triphenylborane **14** (242 mg, 1.0 mmol), sodium hydrogen carbonate (200 mg, 2.4 mmol) and diphenyliodonium fluoride **2b** (360 mg, 1.2 mmol) in 1,2-dichloroethane (20 mL) at room temperature for 12 hours. Purification by flash chromatography (100% hexane) gave compound **14** (105 mg, 68%) as a white crystalline solid.

R_f 0.52 (100% hexane); **m.p.** 69-70 °C (lit. 68-71 °C); **IR** υ max (film, cm^{-1}): 3065, 3035, 2935, 2863, 1620, 1507, 1430, 1240, 1229, 1050, 1031; **^{1}H NMR** (400 MHz, $CDCl_3$): δ = 7.63-7.61 (m, 4H), 7.47 (t, *J*= 7.7 Hz, 4H), 7.39-7.35 (m, 2H); **^{13}C NMR** (101 MHz, $CDCl_3$) δ 141.7, 129.3, 127.7 (2 Cs); **HRMS** (ESI) calculated for $C_{12}H_{11}$ $[M + H]^+$ requires 155.0855, found 155.0852.

These data are in accordance with those of the commercial chemical (CAS Number: 92-52-4).

5. Arylation of Phenols

5.1 General Procedure B for the Arylation of Phenols With Diaryliodonium Salts and Tetrabutylammonium Fluoride

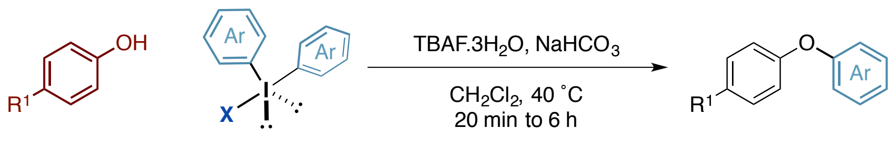

1 **2a,c-j** **3a,s-z,aa,ab**

To a round-bottomed flask was charged the phenol (1.0 mmol, 1 equiv), sodium hydrogen carbonate (2.4 mmol, 2.4 equiv) and diaryliodonium salt (1.2 mmol, 1.2 equiv). This was taken up in dichloromethane (20 mL), followed by tetrabutylammonium fluoride trihydrate (1.2 mmol, 1.2 equiv) and the reaction stirred at 40 °C for the specified length of time. The reaction mixture was diluted with water (20 mL) and the organic layer extracted with dichloromethane (2 x 10 mL), dried over $MgSO_4$ and concentrated *in vacuo*. The crude residue was purified by flash chromatography to yield the corresponding diaryl ether.

Diaryliodonium salts **8a-h** were synthesised from known literature procedures.

Salt	Structure	Literature Procedure for Synthesis
**2c**	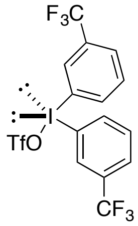	M. Bielawski, D. Aili and B. Olofsson, *J. Org. Chem.* **2008**, *73*, 4602.
**2d**	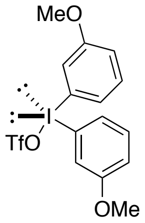	M. Zhu, N. Jalalian and B. Olofsson, *Synlett*, **2008**, *4*, 592.

**2e**	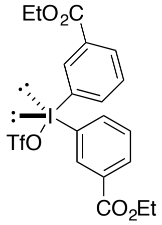	M. Bielawski and B Olofsson, *Chem. Commun.* **2007**, 2521.
**2f**	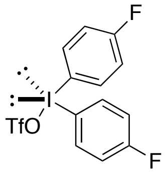	M. Bielawski and B Olofsson, *Chem. Commun.* **2007**, 2521.
**2g**	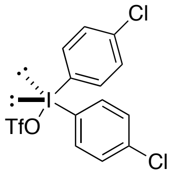	M. Bielawski and B Olofsson, *Chem. Commun.* **2007**, 2521.
**2h**	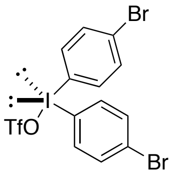	M. Bielawski, M. Zhu and B. Olofsson, *Adv. Synth. Catal.* **2007**, *349*, 2610.
**2i**	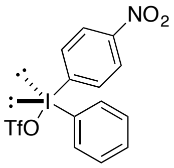	M. Bielawski and B Olofsson, *Chem. Commun.* **2007**, 2521.
**2j**	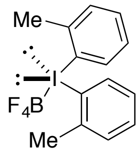	B. S. L. Collins, M. G. Suero and M. J. Gaunt, *Angew. Chem. Int. Ed.* **2013**, *52*, 5799.

Diphenylether (3a)

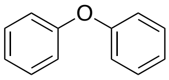

Prepared according to General Procedure B from phenol (94 mg, 1.0 mmol), sodium hydrogen carbonate (200 mg, 2.4 mmol), diphenyliodonium triflate **2a** (516 mg, 1.2 mmol) and tetrabutylammonium fluoride trihydrate (188 mg, 0.7 mmol) in dichloromethane (20 mL) at 40 °C for 2 hours. Purification by flash chromatography (petroleum ether 40-60°) gave compound **3a** (136 mg, 80%) as a colourless oil.

R$_f$ 0.47 (petroleum ether 40-60°); **IR** υ max (film, cm^{-1}): 3040, 2342, 1583, 1486, 1233; **^{1}H NMR** (400 MHz, $CDCl_3$): δ 7.37-7.33 (m, 4H), 7.11 (tt, J = 7.1, 1.1 Hz, 2H), 7.05-7.02 (m, 4H); **^{13}C NMR** (101 MHz, $CDCl_3$): δ 157.7, 130.2, 123.7, 119.3; **HRMS** (ESI) calculated for $C_{12}H_{11}O$ [M + H]$^+$ m/z requires 171.0810, found 171.0803.

These data are in accordance with those of the commercial chemical (CAS Number: 101-84-8).

1-phenoxy-3-(trifluoromethyl)benzene (3s)

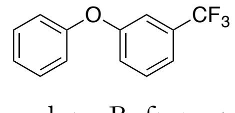

Prepared according to General Procedure B from phenol (56 mg, 0.6 mmol), sodium hydrogen carbonate (117 mg, 1.4 mmol), diaryliodonium salt **2c** (409 mg, 0.7 mmol) and tetrabutylammonium fluoride trihydrate (188 mg, 0.7 mmol) in dichloromethane (15 mL) at 40 °C for 4 hours. Purification by flash chromatography (petroleum ether 40-60°) gave compound **3s** (110 mg, 78%) as a colourless oil.

R$_f$ 0.55 (petroleum ether 40-60°); **IR** υ max (film, cm^{-1}): 3068, 2326, 1587, 1489, 1448, 1326, 1125; **^{1}H NMR** (400 MHz, $CDCl_3$): δ 7.46-7.34 (m, 4H), 7.26 (s, 1H), 7.21-7.16 (m, 2H), 7.06-7.03 (m, 2H); **^{13}C NMR** (101 MHz, $CDCl_3$): δ 158.1, 156.3, 132.5 (q, $^2J_{F\text{-}C}$ = 32.6 Hz), 130.5, 130.2, 124.4, 123.9 (q, $^1J_{F\text{-}C}$ = 272.7 Hz), 121.7, 119.7 (q, $^3J_{F\text{-}C}$ = 3.9 Hz), 119.6, 115.47 (q, $^3J_{F\text{-}C}$ = 3.9 Hz); **^{19}F NMR** (376 MHz, $CDCl_3$): δ -78.5; **HRMS** (ESI) calculated for $C_{13}H_{10}F_3O$ [M + H]$^+$ m/z requires 239.0684, found 239.0673.

These data are in accordance with the literature.[8]

1-methoxy-3-phenoxybenzene (3t)

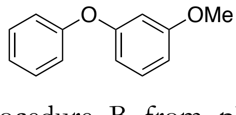

Prepared according to General Procedure B from phenol (94 mg, 1.0 mmol), sodium hydrogen carbonate (200 mg, 2.4 mmol), diaryliodonium salt **2d** (587 mg, 1.2 mmol) and tetrabutylammonium fluoride trihydrate (314 mg, 1.2 mmol) in dichloromethane (20 mL) at 40 °C for 4 hours. Purification by flash chromatography (petroleum ether 40-60°) gave compound **3t** (153 mg, 77%) as a colourless oil.

R_f 0.09 (petroleum ether 40-60°); **IR** υ max (film, cm^{-1}): 3002, 2951, 2835, 1589, 1504, 1488, 1221; **^{1}H NMR** (400 MHz, $CDCl_3$): δ 7.33-7.28 (m, 2H), 7.07-7.04 (m, 1H), 7.03-6.94 (m, 4H), 6.92-6.88(m, 2H), 3.81 (s, 3H); **^{13}C NMR** (101 MHz, $CDCl_3$): δ 158.7, 156.1, 150.3, 129.8, 129.7, 122.6, 121.0, 117.7, 116.6, 115.0, 55.8; **HRMS** (ESI) calculated for $C_{13}H_{13}O_2$ $[M + H]^+$ m/z requires 201.0916, found 201.0907.

These data are in accordance with the literature.[8]

ethyl 3-phenoxybenzoate (3u)

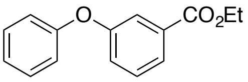

Prepared according to General Procedure B from phenol (94 mg, 1.0 mmol), sodium hydrogen carbonate (200 mg, 2.4 mmol), diaryliodonium salt **2e** (689 mg, 1.2 mmol) and tetrabutylammonium fluoride trihydrate (314 mg, 1.2 mmol) in dichloromethane (20 mL) at 40 °C for 4 hours. Purification by flash chromatography (2.5% diethyl ether in petroleum ether 40-60°) gave compound **3u** (199 mg, 82%) as a colourless oil.

R_f 0.22 (5% diethyl ether in petroleum ether 40-60°); **IR** υ max (film, cm^{-1}): 2989, 1712, 1587, 1489, 1273, 1238; **^{1}H NMR** (400 MHz, $CDCl_3$): δ 8.01-7.97 (m, 2H), 7.36-7.34 (m, 2H), 7.18-7.14 (m, 1H), 7.05-7.02 (m, 2H), 6.98-6.95 (m, 2H), 4.34 (q, *J* = 7.1 Hz, 2H), 1.36 (t, *J* = 7.1 Hz, 3H); **^{13}C NMR** (101 MHz, $CDCl_3$): δ 166.2, 161.8, 155.8, 131.7, 130.0, 124.9, 124.5, 120.1, 117.4, 60.9, 14.4; **HRMS** (ESI) calculated for $C_{15}H_{15}O_3$ $[M + H]^+$ m/z requires 243.1021, found 243.1016.

1-fluoro-4-phenoxybenzene (3v)

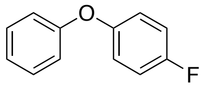

Prepared according to General Procedure B from phenol (94 mg, 1.0 mmol), sodium hydrogen carbonate (200 mg, 2.4 mmol), diaryliodonium salt **2f** (559 mg, 1.2 mmol) and tetrabutylammonium fluoride trihydrate (314 mg, 1.2 mmol) in dichloromethane (20 mL) at 40 °C for 3 hours. Purification by flash chromatography (petroleum ether 40-60°) gave compound **3v** (140 mg, 74%) as a colourless oil.

R_f 0.35 (petroleum ether 40-60°); **IR** υ max (film, cm^{-1}): 3068, 2363, 1589, 1501, 1486, 1209; **^{1}H NMR** (400 MHz, $CDCl_3$): δ 7.36-7.31 (m, 2H), 7.12-7.08 (m, 2H), 7.08-6.97 (m, 5H); **^{13}C**

NMR (101 MHz, $CDCl_3$): δ 160.2, 157.8 (d, $^4J_{F\text{-}C}$ = 6.9 Hz), 153.0 (d, $^3J_{F\text{-}C}$ = 2.5 Hz), 130.0, 123.3, 120.7 (d, $^2J_{F\text{-}C}$ = 8.3 Hz), 118.4, 116.4 (d, $^1J_{F\text{-}C}$ = 23.3 Hz); **^{19}F NMR** (376 MHz, $CDCl_3$): δ 120.4; **HRMS** (ESI) calculated for $C_{12}H_{10}FO$ $[M + H]^+$ m/z requires 189.0716, found 189.0706.

These data are in accordance with the literature.[3]

1-chloro-4-phenoxybenzene (3w)

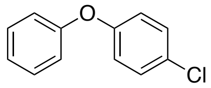

Prepared according to General Procedure B from phenol (94 mg, 1.0 mmol), sodium hydrogen carbonate (200 mg, 2.4 mmol), diaryliodonium salt **2g** (597 mg, 1.2 mmol) and tetrabutylammonium fluoride trihydrate (314 mg, 1.2 mmol) in dichloromethane (20 mL) at 40 °C for 3 hours. Purification by flash chromatography (petroleum ether 40-60°) gave compound **3w** (212 mg, 100%) as a colourless oil.

R_f 0.74 (petroleum ether 40-60°); **IR** υ max (film, cm^{-1}): 2402, 2251, 1584, 1483, 1239; **^{1}H NMR** (400 MHz, $CDCl_3$): δ 7.38-7.33 (m, 2H), 7.31-7.27 (m, 2H), 7.13 (tt, *J* = 7.4, 1.1 Hz, 1H), 7.03-6.99 (m, 2H), 6.97-6.93 (m, 2H); **^{13}C NMR** (101 MHz, $CDCl_3$): δ 157.0, 156.1, 130.0, 129.9, 128.4, 123.8, 120.2, 119.1; **HRMS** (ESI) calculated for $C_{12}H_{10}{}^{35}ClO$ $[M + H]^+$ m/z requires 205.0420, found 205.0411.

These data are in accordance with the literature.[10]

1-bromo-4-phenoxybenzene (3x)

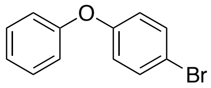

Prepared according to General Procedure B from phenol (47 mg, 0.5 mmol), sodium hydrogen carbonate (100 mg, 1.2 mmol), diaryliodonium salt **2h** (352 mg, 0.6 mmol) and tetrabutylammonium fluoride trihydrate (157 mg, 0.6 mmol) in dichloromethane (10 mL) at 40 °C for 4 hours. Purification by flash chromatography (petroleum ether 40-60°) gave compound **3x** (116 mg, 94%) as a colourless oil.

[10] M. E. Sloan, A. Staubitz, K. Lee and I. Manners, *Eur. J. Org. Chem.* **2011**, *4*, 672.

R$_f$ 0.62 (petroleum ether 40-60°); **IR** υ max (film, cm^{-1}): 3040, 1875, 1578, 1486, 1232; **^{1}H NMR** (400 MHz, $CDCl_3$): δ 7.46-7.42 (m, 2H), 7.38-7.33 (m, 2H), 7.16-7.12 (m, 1H), 7.03-7.00 (m, 2H), 6.92-6.88 (m, 2H); **^{13}C NMR** (101 MHz, $CDCl_3$): δ 156.9, 156.7, 132.8, 130.0, 123.9, 120.6, 119.2, 115.8; **HRMS** (ESI) calculated for $C_{12}H_{10}{}^{79}BrO$ [M + H]$^+$ m/z requires 248.9915, found 248.9907.

These data are in accordance with the literature.[10]

1-nitro-4-phenoxybenzene (3c)

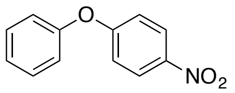

Prepared according to General Procedure B from phenol (94 mg, 1.0 mmol), sodium hydrogen carbonate (200 mg, 2.4 mmol), diaryliodonium salt **2i** (570 mg, 1.2 mmol) and tetrabutylammonium fluoride trihydrate (314 mg, 1.2 mmol) in dichloromethane (20 mL) at 40 °C for 4 hours. Purification by flash chromatography (5% diethyl ether in petroleum ether 40-60°) gave compound **3c** (183 mg, 85%) as a yellow solid.

R$_f$ 0.74 (5% diethyl ether in petroleum ether 40-60°); **m.p.** 56-57 °C; **IR** υ max (film, cm^{-1}): 3020, 2343, 1583, 1515, 1487, 1343, 1247; **^{1}H NMR** (400 MHz, $CDCl_3$): δ 8.21-8.19 (m, 2H), 7.46-7.41 (m, 2H), 7.28-7.24 (m, 1H), 7.11-7.08 (m, 2H), 7.03-6.99 (m, 2H); **^{13}C NMR** (101 MHz, $CDCl_3$): δ 163.2, 154.5, 142.5, 130.2, 125.8, 125.3, 120.4, 117.0; **HRMS** (ESI) calculated for $C_{12}H_{10}NO_3$ [M + H]$^+$ m/z requires 216.0661, found 216.0655.

These data are in accordance with the literature.[3]

1-methyl-2-phenoxybenzene (3y)

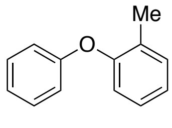

Prepared according to General Procedure B from phenol (94 mg, 1.0 mmol), sodium hydrogen carbonate (200 mg, 2.4 mmol), diaryliodonium salt **2j** (475 mg, 1.2 mmol) and tetrabutylammonium fluoride trihydrate (314 mg, 1.2 mmol) in dichloromethane (20 mL) at 40 °C for 2 hours. Purification by flash chromatography (petroleum ether 40-60°) gave compound **3y** (143 mg, 78%) as a colourless oil.

R_f 0.36 (petroleum ether 40-60°); **IR** υ max (film, cm^{-1}): 2367, 2255, 1488, 1265, 1238, 907, 731; **^{1}H NMR** (400 MHz, $CDCl_3$): δ 7.35-7.26 (m, 3H), 7.19 (td, *J* = 7.98, 1.23, 0.52 Hz, 1H), 7.12-7.05 (m, 2H), 6.95-6.93 (m, 3H), 2.28 (s, 3H); **^{13}C NMR** (101 MHz, $CDCl_3$): δ 157.8, 154.3, 131.3, 129.9, 129.5, 127.0, 123.9, 122.2, 118.6, 117.1, 16.1; **HRMS** (ESI) calculated for $C_{13}H_{13}O$ $[M + H]^+$ m/z requires 185.0966, found 185.0960.

These data are in accordance with the literature.[4]

3-(4-(4-chlorophenoxy)phenyl)thiophene (3z)

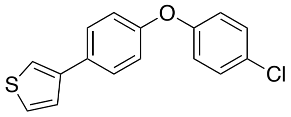

Prepared according to General Procedure B from 4-(thiophen-3-yl)phenol (176 mg, 1.0 mmol), sodium hydrogen carbonate (200 mg, 2.4 mmol), diaryliodonium salt **2g** (597 mg, 1.2 mmol) and tetrabutylammonium fluoride trihydrate (314 mg, 1.2 mmol) in dichloromethane (20 mL) at 40 °C for 2.5 hours. Purification by flash chromatography (2% diethyl ether in petroleum ether 40-60°) gave compound **3z** (224 mg, 78%) as an off-white solid.

R_f 0.43 (petroleum ether 40-60°); **m.p.** 142-143 °C; **IR** υ max (film, cm^{-1}): 2252, 1608, 1591, 1532, 1500, 1486; **^{1}H NMR** (400 MHz, $CDCl_3$): δ 7.57-7.54 (m, 2H), 7.38-7.33 (m, 3H), 7.31-7.27 (m, 2H), 7.02-6.95 (m, 4H); **^{13}C NMR** (101 MHz, $CDCl_3$): δ 156.2, 156.1, 141.7, 131.8, 129.9, 128.5, 128.1, 126.5, 126.4, 120.2, 120.0, 119.4; **HRMS** (ESI) calculated for $C_{16}H_{12}{}^{35}ClOS$ $[M + H]^+$ m/z requires 287.0297, found 287.0294.

ethyl 3-(4-(thiophen-3-yl)phenoxy)benzoate (3aa)

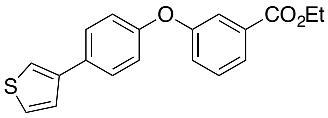

Prepared according to General Procedure B from 4-(thiophen-3-yl)phenol (176 mg, 1.0 mmol), sodium hydrogen carbonate (200 mg, 2.4 mmol), diaryliodonium salt **2e** (689 mg, 1.2 mmol) and tetrabutylammonium fluoride trihydrate (314 mg, 1.2 mmol) in dichloromethane (20 mL) at 40 °C for 2.5 hours. Purification by flash chromatography (5% diethyl ether in petroleum ether 40-60°) gave compound **3aa** (245 mg, 76%) as a white solid.

R_f 0.15 (5% diethyl ether in petroleum ether 40-60°); **m.p.** 108-109 °C; **IR** υ max (film, cm^{-1}): 2252, 1709, 1598, 1497, 1278, 1242; **^{1}H NMR** (400 MHz, $CDCl_3$): δ 8.04-8.00 (m, 2H), 7.62-7.58 (m, 2H), 7.42-7.35 (m, 3H), 7.10-7.00 (m, 4H), 4.39-4.33 (q, *J* = 7.1 Hz, 2H), 1.40-1.36 (t, *J* = 7.1 Hz, 3H); **^{13}C NMR** (101 MHz, $CDCl_3$): δ 166.0, 161.5, 154.7, 141.3, 132.2, 131.5, 127.8, 126.3, 126.1, 124.8, 120.2, 119.9, 117.2, 60.7, 14.2; **HRMS** (ESI) calculated for $C_{19}H_{17}O_3S$ $[M + H]^+$ m/z requires 325.0898, found 325.0893.

3-(4-(4-nitrophenoxy)phenyl)thiophene (3ab)

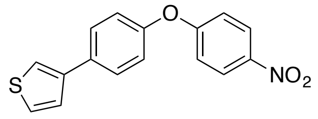

Prepared according to General Procedure B from 4-(thiophen-3-yl)phenol (176 mg, 1.0 mmol), sodium hydrogen carbonate (200 mg, 2.4 mmol), diaryliodonium salt **2i** (570 mg, 1.2 mmol) and tetrabutylammonium fluoride trihydrate (314 mg, 1.2 mmol) in dichloromethane (20 mL) at 40 °C for 2.5 hours. Purification by flash chromatography (10% diethyl ether in petroleum ether 40-60°) gave compound **3ab** (253 mg, 85%) as an off-white solid.

R_f 0.58 (10% diethyl ether in petroleum ether 40-60°); **m.p.** 124-125 °C; **IR** υ max (film, cm^{-1}): 2257, 1588, 1489, 1344, 1246; **^{1}H NMR** (400 MHz, $CDCl_3$): δ 8.24-8.20 (m, 2H), 7.67-7.64 (m, 2H), 7.46-7.38 (m, 3H), 7.14-7.03 (m, 4H); **^{13}C NMR** (101 MHz, $CDCl_3$): δ 163.4, 153.8, 142.7, 141.2, 133.4, 128.3, 126.7, 126.2, 126.0, 120.9, 120.5, 117.2; **HRMS** (ESI) calculated for $C_{16}H_{12}NO_3S$ $[M + H]^+$ m/z requires 298.0538, found 298.0537.

ethyl 2-(4-bromophenyl)-2-cyano-2-phenylacetate (13)

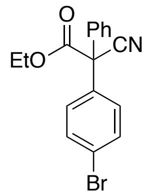

Prepared according to General Procedure B from ethyl 2-cyano-2-phenylacetate **11** (189 mg, 1.0 mmol), sodium hydrogen carbonate (200 mg, 2.4 mmol), diaryliodonium salt **8f** (707 mg, 1.2 mmol) and tetrabutylammonium fluoride trihydrate (314 mg, 1.2 mmol) in dichloromethane (20 mL) at 40 °C for 2 hours. Purification by flash chromatography (5% diethyl ether in petroleum ether 40-60°) gave compound **13** (252 mg, 73%) as a colourless oil.

R_f 0.22 (5% diethyl ether in petroleum ether 40-60°); **IR** υ max (film, cm^{-1}): 3651, 2254, 1747, 1490, 1231, 1012, 905, 729; **^{1}H NMR** (400 MHz, $CDCl_3$): δ = 7.51-7.48 (m, 2H,), 7.39-7.33 (m, 5H,), 7.27-7.22 (m, 2H,), 4.32 (q, *J*= 7.1 Hz, 2H), 1.29 (t, *J*= 7.1 Hz, 3H); **^{13}C NMR** (100 MHz, $CDCl_3$) δ 166.9, 135.5, 135.1, 132.2, 129.9, 129.3 (2 C's), 127.9, 123.5, 118.4, 64.1, 58.4, 14.0; **HRMS** (ESI) calculated for $C_{17}H_{18}{}^{79}BrN_2O_2$ $[M + NH_4]^+$ requires 361.0552, found 361.0550.

4-bromo-1,1'-biphenyl (16)

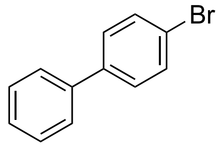

Prepared according to General Procedure B from triphenylborane **14** (242 mg, 1.0 mmol), sodium hydrogen carbonate (200 mg, 2.4 mmol), diaryliodonium salt **8f** (707 mg, 1.2 mmol) and tetrabutylammonium fluoride trihydrate (314 mg, 1.2 mmol) in 1,2-dichloroethane (20 mL) at room temperature for 12 hours. Purification by flash chromatography (100% hexane) gave compound **16** (166 mg, 72%) as a white crystalline solid.

R_f 0.60 (100% hexane); **m.p.** 83-84 °C (lit. 82-86 °C); **IR** υ max (film, cm^{-1}): 3065, 3034, 1587, 1475, 1393, 830, 756; **^{1}H NMR** (400 MHz, $CDCl_3$): δ = 7.59-7.55 (m, 4H,), 7.48-7.44 (m, 4H), 7.40-7.36 (m, 1H); **^{13}C NMR** (100 MHz, $CDCl_3$) δ 140.6, 140.5, 132.4, 129.4, 128.1, 127.4, 122.0; **HRMS** (ESI) calculated for $C_{12}H_{10}{}^{79}Br$ $[M + H]^+$ requires 234.9940, found 234.9936.

These data are in accordance with those of the commercial chemical (CAS Number: 92-66-0).
